# Supplementary material for: Conformational changes of surfactant protein B due to the alveolar air/liquid interface using molecular dynamics
Source: J Mol Model. 2025 Dec 4;32(1):4. doi: 10.1007/s00894-025-06585-4 (PMC12678512; doi:10.1007/s00894-025-06585-4)
Supplement: Supplementary file 1 — The following information is available free of charge in the supporting information: per residue RMSF for all simulation systems (SI page S2) and the SP-B closed (SI pages S3-S40) and open (SI pages S41-S78) conformation PDBs (DOCX 174 KB) [file 894_2025_6585_MOESM1_ESM.docx]

**Supplemental Information**

Table of Contents

Page Item

S1 Table of Contents

S2 Figure S1: Per residue RMSF in all simulation systems

S3-S40 SP-B Closed PDB

S41-S78 SP-B Open PDB

| 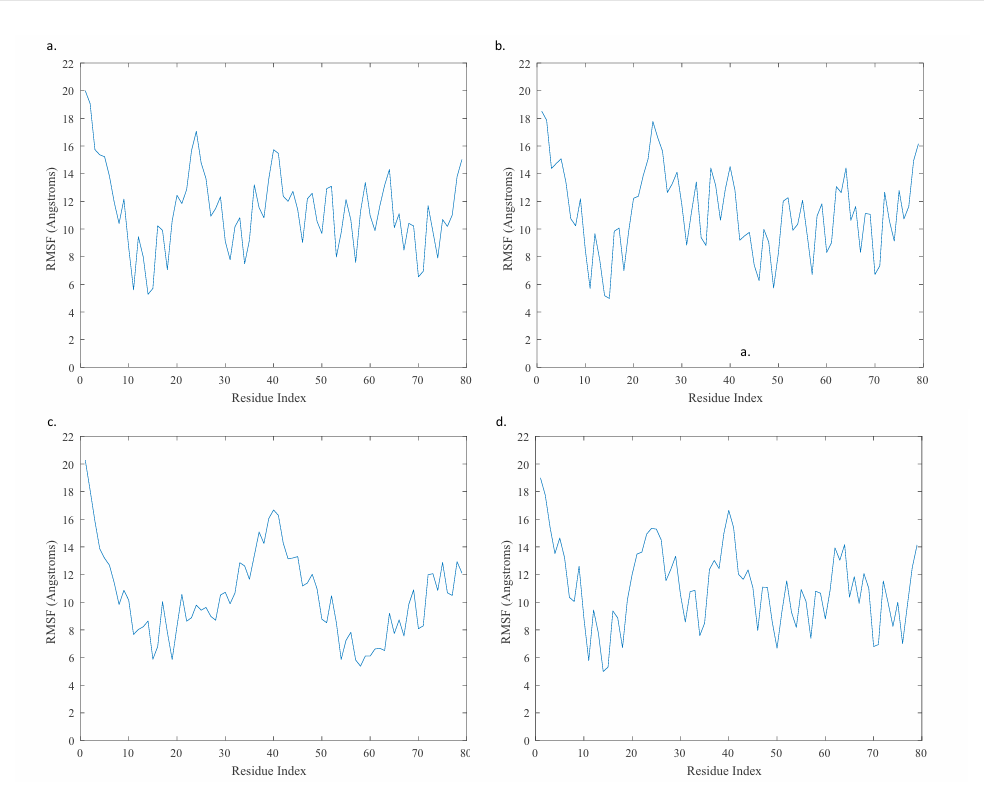  **Figure S1** Per residue RMSF in each simulation system. **a,** Open conformation SP-B in water **b,** Closed conformation SP-B in water **c,** Open conformation SP-B in chloroform **d,** Closed conformation SP-B in chloroform. |
| --- |

SP-B Closed PDB

ATOM 1 N PHE 1 22.457 -1.776 38.724 0.1737 1.5500 N

ATOM 2 H1 PHE 1 22.062 -2.458 39.354 0.1921 1.3000 H

ATOM 3 H2 PHE 1 21.724 -1.190 38.350 0.1921 1.3000 H

ATOM 4 H3 PHE 1 22.919 -2.248 37.959 0.1921 1.3000 H

ATOM 5 CA PHE 1 23.429 -0.951 39.436 0.0733 1.7000 C

ATOM 6 HA PHE 1 22.926 -0.435 40.253 0.1041 1.2000 H

ATOM 7 CB PHE 1 24.516 -1.876 40.011 0.0330 1.7000 C

ATOM 8 HB2 PHE 1 25.222 -1.282 40.592 0.0104 1.2000 H

ATOM 9 HB3 PHE 1 24.047 -2.578 40.702 0.0104 1.2000 H

ATOM 10 CG PHE 1 25.282 -2.662 38.955 0.0031 1.7000 C

ATOM 11 CD1 PHE 1 26.409 -2.132 38.390 -0.1392 1.7000 C

ATOM 12 HD1 PHE 1 26.789 -1.207 38.775 0.1374 1.2000 H

ATOM 13 CE1 PHE 1 27.122 -2.805 37.421 -0.1602 1.7000 C

ATOM 14 HE1 PHE 1 28.020 -2.369 37.007 0.1433 1.2000 H

ATOM 15 CZ PHE 1 26.708 -4.041 37.002 -0.1208 1.7000 C

ATOM 16 HZ PHE 1 27.269 -4.579 36.252 0.1329 1.2000 H

ATOM 17 CE2 PHE 1 25.583 -4.601 37.558 -0.1603 1.7000 C

ATOM 18 HE2 PHE 1 25.268 -5.583 37.248 0.1433 1.2000 H

ATOM 19 CD2 PHE 1 24.866 -3.916 38.529 -0.1391 1.7000 C

ATOM 20 HD2 PHE 1 23.985 -4.370 38.954 0.1374 1.2000 H

ATOM 21 C PHE 1 24.032 0.085 38.467 0.6123 1.7000 C

ATOM 22 O PHE 1 23.907 -0.092 37.258 -0.5713 1.5000 O

ATOM 23 N PRO 2 24.624 1.208 38.942 -0.2548 1.5500 N

ATOM 24 CD PRO 2 24.570 1.561 40.383 0.0192 1.7000 C

ATOM 25 HD2 PRO 2 25.327 1.002 40.933 0.0391 1.2000 H

ATOM 26 HD3 PRO 2 23.578 1.420 40.813 0.0391 1.2000 H

ATOM 27 CG PRO 2 24.931 3.034 40.351 0.0189 1.7000 C

ATOM 28 HG2 PRO 2 25.418 3.344 41.277 0.0213 1.2000 H

ATOM 29 HG3 PRO 2 24.028 3.625 40.187 0.0213 1.2000 H

ATOM 30 CB PRO 2 25.853 3.184 39.161 -0.0070 1.7000 C

ATOM 31 HB2 PRO 2 26.863 2.901 39.462 0.0253 1.2000 H

ATOM 32 HB3 PRO 2 25.852 4.207 38.784 0.0253 1.2000 H

ATOM 33 CA PRO 2 25.302 2.205 38.117 -0.0266 1.7000 C

ATOM 34 HA PRO 2 24.578 2.704 37.471 0.0641 1.2000 H

ATOM 35 C PRO 2 26.431 1.587 37.309 0.5896 1.7000 C

ATOM 36 O PRO 2 27.252 0.860 37.862 -0.5748 1.5000 O

ATOM 37 N ILE 3 26.540 1.952 36.040 -0.4157 1.5500 N

ATOM 38 H ILE 3 25.865 2.583 35.634 0.2719 1.3000 H

ATOM 39 CA ILE 3 27.603 1.424 35.189 -0.0597 1.7000 C

ATOM 40 HA ILE 3 28.191 0.692 35.737 0.0869 1.2000 H

ATOM 41 CB ILE 3 26.994 0.617 34.040 0.1303 1.7000 C

ATOM 42 HB ILE 3 26.328 1.264 33.463 0.0187 1.2000 H

ATOM 43 CG2 ILE 3 28.065 0.130 33.151 -0.3204 1.7000 C

ATOM 44 HG21 ILE 3 27.602 -0.418 32.339 0.0882 1.2000 H

ATOM 45 HG22 ILE 3 28.625 0.939 32.686 0.0882 1.2000 H

ATOM 46 HG23 ILE 3 28.749 -0.528 33.689 0.0882 1.2000 H

ATOM 47 CG1 ILE 3 26.159 -0.559 34.620 -0.0430 1.7000 C

ATOM 48 HG12 ILE 3 26.828 -1.247 35.137 0.0236 1.2000 H

ATOM 49 HG13 ILE 3 25.444 -0.178 35.339 0.0236 1.2000 H

ATOM 50 CD1 ILE 3 25.352 -1.341 33.623 -0.0660 1.7000 C

ATOM 51 HD11 ILE 3 24.789 -2.114 34.145 0.0186 1.2000 H

ATOM 52 HD12 ILE 3 24.647 -0.677 33.125 0.0186 1.2000 H

ATOM 53 HD13 ILE 3 26.001 -1.819 32.894 0.0186 1.2000 H

ATOM 54 C ILE 3 28.525 2.525 34.635 0.5973 1.7000 C

ATOM 55 O ILE 3 28.111 3.265 33.742 -0.5679 1.5000 O

ATOM 56 N PRO 4 29.759 2.685 35.156 -0.2548 1.5500 N

ATOM 57 CD PRO 4 30.215 1.861 36.295 0.0192 1.7000 C

ATOM 58 HD2 PRO 4 30.589 0.901 35.937 0.0391 1.2000 H

ATOM 59 HD3 PRO 4 29.454 1.738 37.063 0.0391 1.2000 H

ATOM 60 CG PRO 4 31.342 2.695 36.852 0.0189 1.7000 C

ATOM 61 HG2 PRO 4 32.092 2.073 37.343 0.0213 1.2000 H

ATOM 62 HG3 PRO 4 30.938 3.423 37.557 0.0213 1.2000 H

ATOM 63 CB PRO 4 31.917 3.418 35.662 -0.0070 1.7000 C

ATOM 64 HB2 PRO 4 32.625 2.776 35.164 0.0253 1.2000 H

ATOM 65 HB3 PRO 4 32.409 4.345 35.960 0.0253 1.2000 H

ATOM 66 CA PRO 4 30.725 3.692 34.746 -0.0266 1.7000 C

ATOM 67 HA PRO 4 30.318 4.684 34.949 0.0641 1.2000 H

ATOM 68 C PRO 4 31.091 3.547 33.271 0.5896 1.7000 C

ATOM 69 O PRO 4 31.234 2.431 32.752 -0.5748 1.5000 O

ATOM 70 N LEU 5 31.359 4.669 32.620 -0.4157 1.5500 N

ATOM 71 H LEU 5 31.264 5.565 33.076 0.2719 1.3000 H

ATOM 72 CA LEU 5 31.694 4.627 31.208 -0.0518 1.7000 C

ATOM 73 HA LEU 5 30.849 4.159 30.708 0.0922 1.2000 H

ATOM 74 CB LEU 5 31.849 6.049 30.673 -0.1102 1.7000 C

ATOM 75 HB2 LEU 5 32.527 6.605 31.318 0.0457 1.2000 H

ATOM 76 HB3 LEU 5 32.317 5.995 29.694 0.0457 1.2000 H

ATOM 77 CG LEU 5 30.561 6.821 30.553 0.3531 1.7000 C

ATOM 78 HG LEU 5 30.013 6.788 31.496 -0.0361 1.2000 H

ATOM 79 CD1 LEU 5 30.859 8.271 30.224 -0.4121 1.7000 C

ATOM 80 HD11 LEU 5 29.927 8.832 30.157 0.1000 1.2000 H

ATOM 81 HD12 LEU 5 31.474 8.715 31.008 0.1000 1.2000 H

ATOM 82 HD13 LEU 5 31.383 8.346 29.273 0.1000 1.2000 H

ATOM 83 CD2 LEU 5 29.728 6.181 29.462 -0.4121 1.7000 C

ATOM 84 HD21 LEU 5 28.872 6.819 29.239 0.1000 1.2000 H

ATOM 85 HD22 LEU 5 30.309 6.056 28.547 0.1000 1.2000 H

ATOM 86 HD23 LEU 5 29.331 5.219 29.781 0.1000 1.2000 H

ATOM 87 C LEU 5 32.904 3.763 30.819 0.5973 1.7000 C

ATOM 88 O LEU 5 32.787 3.054 29.825 -0.5679 1.5000 O

ATOM 89 N PRO 6 34.047 3.738 31.547 -0.2548 1.5500 N

ATOM 90 CD PRO 6 34.276 4.637 32.696 0.0192 1.7000 C

ATOM 91 HD2 PRO 6 33.810 4.228 33.591 0.0391 1.2000 H

ATOM 92 HD3 PRO 6 33.962 5.661 32.512 0.0391 1.2000 H

ATOM 93 CG PRO 6 35.775 4.586 32.842 0.0189 1.7000 C

ATOM 94 HG2 PRO 6 36.081 4.767 33.873 0.0213 1.2000 H

ATOM 95 HG3 PRO 6 36.228 5.325 32.178 0.0213 1.2000 H

ATOM 96 CB PRO 6 36.158 3.203 32.386 -0.0070 1.7000 C

ATOM 97 HB2 PRO 6 36.007 2.502 33.209 0.0253 1.2000 H

ATOM 98 HB3 PRO 6 37.196 3.166 32.052 0.0253 1.2000 H

ATOM 99 CA PRO 6 35.194 2.893 31.239 -0.0266 1.7000 C

ATOM 100 HA PRO 6 35.631 3.204 30.292 0.0641 1.2000 H

ATOM 101 C PRO 6 34.870 1.405 31.199 0.5896 1.7000 C

ATOM 102 O PRO 6 35.537 0.643 30.501 -0.5748 1.5000 O

ATOM 103 N TYR 7 33.811 0.985 31.894 -0.4157 1.5500 N

ATOM 104 H TYR 7 33.248 1.640 32.417 0.2719 1.3000 H

ATOM 105 CA TYR 7 33.452 -0.421 31.927 -0.0014 1.7000 C

ATOM 106 HA TYR 7 34.355 -1.031 31.985 0.0876 1.2000 H

ATOM 107 CB TYR 7 32.627 -0.673 33.181 -0.0152 1.7000 C

ATOM 108 HB2 TYR 7 31.792 0.022 33.245 0.0295 1.2000 H

ATOM 109 HB3 TYR 7 32.213 -1.681 33.128 0.0295 1.2000 H

ATOM 110 CG TYR 7 33.501 -0.555 34.420 -0.0011 1.7000 C

ATOM 111 CD1 TYR 7 34.061 0.659 34.710 -0.1906 1.7000 C

ATOM 112 HD1 TYR 7 33.876 1.537 34.128 0.1699 1.2000 H

ATOM 113 CE1 TYR 7 34.840 0.829 35.829 -0.2341 1.7000 C

ATOM 114 HE1 TYR 7 35.261 1.797 36.059 0.1656 1.2000 H

ATOM 115 CZ TYR 7 35.052 -0.241 36.681 0.3226 1.7000 C

ATOM 116 OH TYR 7 35.825 -0.081 37.811 -0.5579 1.5000 O

ATOM 117 HH TYR 7 35.881 -0.878 38.344 0.3992 1.2000 H

ATOM 118 CE2 TYR 7 34.480 -1.461 36.407 -0.2341 1.7000 C

ATOM 119 HE2 TYR 7 34.626 -2.290 37.083 0.1656 1.2000 H

ATOM 120 CD2 TYR 7 33.701 -1.621 35.280 -0.1906 1.7000 C

ATOM 121 HD2 TYR 7 33.228 -2.572 35.095 0.1699 1.2000 H

ATOM 122 C TYR 7 32.737 -0.744 30.636 0.5973 1.7000 C

ATOM 123 O TYR 7 32.978 -1.785 30.018 -0.5679 1.5000 O

ATOM 124 N CYX 8 31.891 0.184 30.193 -0.4157 1.5500 N

ATOM 125 H CYX 8 31.734 1.027 30.727 0.2719 1.3000 H

ATOM 126 CA CYX 8 31.186 0.001 28.934 0.0429 1.7000 C

ATOM 127 HA CYX 8 30.750 -0.998 28.924 0.0766 1.2000 H

ATOM 128 CB CYX 8 30.068 1.023 28.758 -0.0790 1.7000 C

ATOM 129 HB2 CYX 8 29.395 0.950 29.611 0.0910 1.2000 H

ATOM 130 HB3 CYX 8 30.491 2.028 28.751 0.0910 1.2000 H

ATOM 131 SG CYX 8 29.102 0.781 27.226 -0.1081 1.8000 S

ATOM 132 C CYX 8 32.135 0.133 27.754 0.5973 1.7000 C

ATOM 133 O CYX 8 32.066 -0.641 26.791 -0.5679 1.5000 O

ATOM 134 N TRP 9 33.016 1.127 27.816 -0.4157 1.5500 N

ATOM 135 H TRP 9 33.041 1.737 28.618 0.2719 1.3000 H

ATOM 136 CA TRP 9 33.945 1.362 26.730 -0.0275 1.7000 C

ATOM 137 HA TRP 9 33.396 1.549 25.806 0.1123 1.2000 H

ATOM 138 CB TRP 9 34.833 2.558 27.058 -0.0050 1.7000 C

ATOM 139 HB2 TRP 9 35.262 2.437 28.055 0.0339 1.2000 H

ATOM 140 HB3 TRP 9 35.653 2.593 26.341 0.0339 1.2000 H

ATOM 141 CG TRP 9 34.107 3.873 26.983 -0.1415 1.7000 C

ATOM 142 CD1 TRP 9 32.975 4.132 26.272 -0.1638 1.7000 C

ATOM 143 HD1 TRP 9 32.442 3.425 25.655 0.2062 1.2000 H

ATOM 144 NE1 TRP 9 32.604 5.442 26.433 -0.3418 1.5500 N

ATOM 145 HE1 TRP 9 31.807 5.868 25.983 0.3412 1.3000 H

ATOM 146 CE2 TRP 9 33.497 6.057 27.267 0.1380 1.7000 C

ATOM 147 CZ2 TRP 9 33.538 7.360 27.720 -0.2601 1.7000 C

ATOM 148 HZ2 TRP 9 32.795 8.081 27.419 0.1572 1.2000 H

ATOM 149 CH2 TRP 9 34.565 7.707 28.593 -0.1134 1.7000 C

ATOM 150 HH2 TRP 9 34.620 8.720 28.966 0.1417 1.2000 H

ATOM 151 CZ3 TRP 9 35.509 6.798 28.984 -0.1972 1.7000 C

ATOM 152 HZ3 TRP 9 36.302 7.104 29.651 0.1447 1.2000 H

ATOM 153 CE3 TRP 9 35.478 5.495 28.518 -0.2387 1.7000 C

ATOM 154 HE3 TRP 9 36.236 4.788 28.816 0.1700 1.2000 H

ATOM 155 CD2 TRP 9 34.451 5.114 27.648 0.1243 1.7000 C

ATOM 156 C TRP 9 34.787 0.121 26.519 0.5973 1.7000 C

ATOM 157 O TRP 9 34.943 -0.341 25.381 -0.5679 1.5000 O

ATOM 158 N LEU 10 35.279 -0.462 27.617 -0.4157 1.5500 N

ATOM 159 H LEU 10 35.125 -0.056 28.531 0.2719 1.3000 H

ATOM 160 CA LEU 10 36.009 -1.701 27.511 -0.0518 1.7000 C

ATOM 161 HA LEU 10 36.741 -1.583 26.716 0.0922 1.2000 H

ATOM 162 CB LEU 10 36.726 -2.014 28.804 -0.1102 1.7000 C

ATOM 163 HB2 LEU 10 36.008 -1.902 29.618 0.0457 1.2000 H

ATOM 164 HB3 LEU 10 37.040 -3.059 28.798 0.0457 1.2000 H

ATOM 165 CG LEU 10 37.906 -1.143 29.060 0.3531 1.7000 C

ATOM 166 HG LEU 10 37.648 -0.091 28.934 -0.0361 1.2000 H

ATOM 167 CD1 LEU 10 38.368 -1.356 30.437 -0.4121 1.7000 C

ATOM 168 HD11 LEU 10 39.245 -0.745 30.634 0.1000 1.2000 H

ATOM 169 HD12 LEU 10 37.586 -1.046 31.129 0.1000 1.2000 H

ATOM 170 HD13 LEU 10 38.607 -2.405 30.600 0.1000 1.2000 H

ATOM 171 CD2 LEU 10 38.993 -1.526 28.046 -0.4121 1.7000 C

ATOM 172 HD21 LEU 10 39.926 -1.031 28.294 0.1000 1.2000 H

ATOM 173 HD22 LEU 10 39.172 -2.602 28.055 0.1000 1.2000 H

ATOM 174 HD23 LEU 10 38.730 -1.210 27.038 0.1000 1.2000 H

ATOM 175 C LEU 10 35.137 -2.849 27.085 0.5973 1.7000 C

ATOM 176 O LEU 10 35.572 -3.639 26.270 -0.5679 1.5000 O

ATOM 177 N CYX 11 33.894 -2.940 27.553 -0.4157 1.5500 N

ATOM 178 H CYX 11 33.536 -2.273 28.222 0.2719 1.3000 H

ATOM 179 CA CYX 11 33.056 -4.049 27.110 0.0429 1.7000 C

ATOM 180 HA CYX 11 33.491 -4.984 27.467 0.0766 1.2000 H

ATOM 181 CB CYX 11 31.643 -3.927 27.663 -0.0790 1.7000 C

ATOM 182 HB2 CYX 11 31.721 -3.923 28.746 0.0910 1.2000 H

ATOM 183 HB3 CYX 11 31.195 -2.982 27.355 0.0910 1.2000 H

ATOM 184 SG CYX 11 30.543 -5.288 27.169 -0.1081 1.8000 S

ATOM 185 C CYX 11 32.968 -4.091 25.601 0.5973 1.7000 C

ATOM 186 O CYX 11 33.165 -5.133 24.976 -0.5679 1.5000 O

ATOM 187 N ARG 12 32.655 -2.956 25.002 -0.3479 1.5500 N

ATOM 188 H ARG 12 32.511 -2.109 25.535 0.2747 1.3000 H

ATOM 189 CA ARG 12 32.493 -2.941 23.570 -0.2637 1.7000 C

ATOM 190 HA ARG 12 31.848 -3.774 23.281 0.1560 1.2000 H

ATOM 191 CB ARG 12 31.790 -1.658 23.189 -0.0007 1.7000 C

ATOM 192 HB2 ARG 12 32.323 -0.807 23.619 0.0327 1.2000 H

ATOM 193 HB3 ARG 12 31.802 -1.555 22.103 0.0327 1.2000 H

ATOM 194 CG ARG 12 30.335 -1.648 23.661 0.0390 1.7000 C

ATOM 195 HG2 ARG 12 29.801 -2.460 23.163 0.0285 1.2000 H

ATOM 196 HG3 ARG 12 30.305 -1.830 24.736 0.0285 1.2000 H

ATOM 197 CD ARG 12 29.628 -0.381 23.404 0.0486 1.7000 C

ATOM 198 HD2 ARG 12 30.142 0.421 23.937 0.0687 1.2000 H

ATOM 199 HD3 ARG 12 29.644 -0.167 22.335 0.0687 1.2000 H

ATOM 200 NE ARG 12 28.246 -0.461 23.859 -0.5295 1.5500 N

ATOM 201 HE ARG 12 27.919 -1.367 24.160 0.3456 1.3000 H

ATOM 202 CZ ARG 12 27.399 0.579 23.962 0.8076 1.7000 C

ATOM 203 NH1 ARG 12 27.791 1.789 23.629 -0.8627 1.5500 N

ATOM 204 HH11 ARG 12 28.745 1.957 23.347 0.4478 1.3000 H

ATOM 205 HH12 ARG 12 27.150 2.568 23.682 0.4478 1.3000 H

ATOM 206 NH2 ARG 12 26.172 0.380 24.409 -0.8627 1.5500 N

ATOM 207 HH21 ARG 12 25.885 -0.535 24.722 0.4478 1.3000 H

ATOM 208 HH22 ARG 12 25.529 1.154 24.483 0.4478 1.3000 H

ATOM 209 C ARG 12 33.824 -3.123 22.826 0.7341 1.7000 C

ATOM 210 O ARG 12 33.884 -3.865 21.840 -0.5894 1.5000 O

ATOM 211 N ALA 13 34.910 -2.490 23.300 -0.4157 1.5500 N

ATOM 212 H ALA 13 34.847 -1.901 24.118 0.2719 1.3000 H

ATOM 213 CA ALA 13 36.206 -2.660 22.637 0.0337 1.7000 C

ATOM 214 HA ALA 13 36.107 -2.389 21.584 0.0823 1.2000 H

ATOM 215 CB ALA 13 37.251 -1.755 23.273 -0.1825 1.7000 C

ATOM 216 HB1 ALA 13 38.208 -1.885 22.767 0.0603 1.2000 H

ATOM 217 HB2 ALA 13 36.940 -0.713 23.179 0.0603 1.2000 H

ATOM 218 HB3 ALA 13 37.368 -1.995 24.331 0.0603 1.2000 H

ATOM 219 C ALA 13 36.687 -4.100 22.729 0.5973 1.7000 C

ATOM 220 O ALA 13 37.189 -4.674 21.757 -0.5679 1.5000 O

ATOM 221 N LEU 14 36.496 -4.682 23.910 -0.4157 1.5500 N

ATOM 222 H LEU 14 36.064 -4.142 24.642 0.2719 1.3000 H

ATOM 223 CA LEU 14 36.908 -6.028 24.243 -0.0518 1.7000 C

ATOM 224 HA LEU 14 37.963 -6.140 24.019 0.0922 1.2000 H

ATOM 225 CB LEU 14 36.682 -6.222 25.768 -0.1102 1.7000 C

ATOM 226 HB2 LEU 14 37.277 -5.458 26.273 0.0457 1.2000 H

ATOM 227 HB3 LEU 14 35.629 -6.019 25.956 0.0457 1.2000 H

ATOM 228 CG LEU 14 36.954 -7.540 26.478 0.3531 1.7000 C

ATOM 229 HG LEU 14 36.300 -8.316 26.084 -0.0361 1.2000 H

ATOM 230 CD1 LEU 14 38.372 -7.915 26.303 -0.4121 1.7000 C

ATOM 231 HD11 LEU 14 38.609 -8.789 26.905 0.1000 1.2000 H

ATOM 232 HD12 LEU 14 38.567 -8.143 25.257 0.1000 1.2000 H

ATOM 233 HD13 LEU 14 39.018 -7.095 26.599 0.1000 1.2000 H

ATOM 234 CD2 LEU 14 36.655 -7.345 27.984 -0.4121 1.7000 C

ATOM 235 HD21 LEU 14 36.838 -8.275 28.520 0.1000 1.2000 H

ATOM 236 HD22 LEU 14 37.292 -6.567 28.405 0.1000 1.2000 H

ATOM 237 HD23 LEU 14 35.612 -7.062 28.123 0.1000 1.2000 H

ATOM 238 C LEU 14 36.154 -7.005 23.395 0.5973 1.7000 C

ATOM 239 O LEU 14 36.763 -7.778 22.666 -0.5679 1.5000 O

ATOM 240 N ILE 15 34.839 -6.903 23.363 -0.4157 1.5500 N

ATOM 241 H ILE 15 34.357 -6.221 23.932 0.2719 1.3000 H

ATOM 242 CA ILE 15 34.078 -7.849 22.578 -0.0597 1.7000 C

ATOM 243 HA ILE 15 34.320 -8.855 22.929 0.0869 1.2000 H

ATOM 244 CB ILE 15 32.590 -7.633 22.741 0.1303 1.7000 C

ATOM 245 HB ILE 15 32.368 -6.583 22.539 0.0187 1.2000 H

ATOM 246 CG2 ILE 15 31.906 -8.449 21.776 -0.3204 1.7000 C

ATOM 247 HG21 ILE 15 30.832 -8.448 21.920 0.0882 1.2000 H

ATOM 248 HG22 ILE 15 32.017 -8.072 20.761 0.0882 1.2000 H

ATOM 249 HG23 ILE 15 32.256 -9.478 21.830 0.0882 1.2000 H

ATOM 250 CG1 ILE 15 32.143 -7.984 24.108 -0.0430 1.7000 C

ATOM 251 HG12 ILE 15 32.221 -9.056 24.268 0.0236 1.2000 H

ATOM 252 HG13 ILE 15 32.776 -7.516 24.858 0.0236 1.2000 H

ATOM 253 CD1 ILE 15 30.751 -7.546 24.334 -0.0660 1.7000 C

ATOM 254 HD11 ILE 15 30.526 -7.598 25.397 0.0186 1.2000 H

ATOM 255 HD12 ILE 15 30.598 -6.523 23.991 0.0186 1.2000 H

ATOM 256 HD13 ILE 15 30.062 -8.208 23.826 0.0186 1.2000 H

ATOM 257 C ILE 15 34.382 -7.796 21.089 0.5973 1.7000 C

ATOM 258 O ILE 15 34.512 -8.841 20.443 -0.5679 1.5000 O

ATOM 259 N LYS 16 34.476 -6.598 20.510 -0.3479 1.5500 N

ATOM 260 H LYS 16 34.366 -5.748 21.046 0.2747 1.3000 H

ATOM 261 CA LYS 16 34.745 -6.525 19.081 -0.2400 1.7000 C

ATOM 262 HA LYS 16 33.995 -7.111 18.545 0.1426 1.2000 H

ATOM 263 CB LYS 16 34.646 -5.076 18.617 -0.0094 1.7000 C

ATOM 264 HB2 LYS 16 35.277 -4.454 19.256 0.0362 1.2000 H

ATOM 265 HB3 LYS 16 35.036 -5.013 17.599 0.0362 1.2000 H

ATOM 266 CG LYS 16 33.233 -4.522 18.609 0.0187 1.7000 C

ATOM 267 HG2 LYS 16 32.633 -5.102 17.906 0.0103 1.2000 H

ATOM 268 HG3 LYS 16 32.789 -4.614 19.600 0.0103 1.2000 H

ATOM 269 CD LYS 16 33.216 -3.067 18.186 -0.0479 1.7000 C

ATOM 270 HD2 LYS 16 33.832 -2.486 18.878 0.0621 1.2000 H

ATOM 271 HD3 LYS 16 33.634 -2.980 17.181 0.0621 1.2000 H

ATOM 272 CE LYS 16 31.805 -2.501 18.188 -0.0143 1.7000 C

ATOM 273 HE2 LYS 16 31.195 -3.076 17.489 0.1135 1.2000 H

ATOM 274 HE3 LYS 16 31.382 -2.605 19.188 0.1135 1.2000 H

ATOM 275 NZ LYS 16 31.788 -1.063 17.789 -0.3854 1.5500 N

ATOM 276 HZ1 LYS 16 32.192 -0.961 16.868 0.3400 1.3000 H

ATOM 277 HZ2 LYS 16 30.837 -0.723 17.769 0.3400 1.3000 H

ATOM 278 HZ3 LYS 16 32.328 -0.516 18.445 0.3400 1.3000 H

ATOM 279 C LYS 16 36.119 -7.091 18.721 0.7341 1.7000 C

ATOM 280 O LYS 16 36.260 -7.830 17.735 -0.5894 1.5000 O

ATOM 281 N ARG 17 37.133 -6.785 19.539 -0.3479 1.5500 N

ATOM 282 H ARG 17 36.977 -6.201 20.348 0.2747 1.3000 H

ATOM 283 CA ARG 17 38.473 -7.283 19.276 -0.2637 1.7000 C

ATOM 284 HA ARG 17 38.744 -7.083 18.238 0.1560 1.2000 H

ATOM 285 CB ARG 17 39.456 -6.577 20.186 -0.0007 1.7000 C

ATOM 286 HB2 ARG 17 39.095 -6.654 21.211 0.0327 1.2000 H

ATOM 287 HB3 ARG 17 40.420 -7.082 20.124 0.0327 1.2000 H

ATOM 288 CG ARG 17 39.669 -5.109 19.851 0.0390 1.7000 C

ATOM 289 HG2 ARG 17 40.142 -5.048 18.870 0.0285 1.2000 H

ATOM 290 HG3 ARG 17 38.710 -4.595 19.784 0.0285 1.2000 H

ATOM 291 CD ARG 17 40.535 -4.400 20.844 0.0486 1.7000 C

ATOM 292 HD2 ARG 17 40.078 -4.472 21.832 0.0687 1.2000 H

ATOM 293 HD3 ARG 17 41.516 -4.877 20.870 0.0687 1.2000 H

ATOM 294 NE ARG 17 40.686 -2.992 20.485 -0.5295 1.5500 N

ATOM 295 HE ARG 17 40.193 -2.688 19.659 0.3456 1.3000 H

ATOM 296 CZ ARG 17 41.418 -2.074 21.151 0.8076 1.7000 C

ATOM 297 NH1 ARG 17 42.097 -2.390 22.224 -0.8627 1.5500 N

ATOM 298 HH11 ARG 17 42.675 -1.699 22.677 0.4478 1.3000 H

ATOM 299 HH12 ARG 17 42.063 -3.333 22.579 0.4478 1.3000 H

ATOM 300 NH2 ARG 17 41.457 -0.832 20.707 -0.8627 1.5500 N

ATOM 301 HH21 ARG 17 41.001 -0.590 19.842 0.4478 1.3000 H

ATOM 302 HH22 ARG 17 41.956 -0.130 21.229 0.4478 1.3000 H

ATOM 303 C ARG 17 38.504 -8.787 19.489 0.7341 1.7000 C

ATOM 304 O ARG 17 39.096 -9.535 18.707 -0.5894 1.5000 O

ATOM 305 N ILE 18 37.808 -9.244 20.514 -0.4157 1.5500 N

ATOM 306 H ILE 18 37.311 -8.609 21.117 0.2719 1.3000 H

ATOM 307 CA ILE 18 37.754 -10.654 20.783 -0.0597 1.7000 C

ATOM 308 HA ILE 18 38.779 -11.014 20.875 0.0869 1.2000 H

ATOM 309 CB ILE 18 37.038 -10.929 22.092 0.1303 1.7000 C

ATOM 310 HB ILE 18 36.101 -10.369 22.076 0.0187 1.2000 H

ATOM 311 CG2 ILE 18 36.709 -12.281 22.208 -0.3204 1.7000 C

ATOM 312 HG21 ILE 18 36.052 -12.444 23.054 0.0882 1.2000 H

ATOM 313 HG22 ILE 18 36.110 -12.668 21.404 0.0882 1.2000 H

ATOM 314 HG23 ILE 18 37.601 -12.890 22.303 0.0882 1.2000 H

ATOM 315 CG1 ILE 18 37.837 -10.502 23.256 -0.0430 1.7000 C

ATOM 316 HG12 ILE 18 38.678 -11.180 23.401 0.0236 1.2000 H

ATOM 317 HG13 ILE 18 38.279 -9.522 23.111 0.0236 1.2000 H

ATOM 318 CD1 ILE 18 37.029 -10.502 24.460 -0.0660 1.7000 C

ATOM 319 HD11 ILE 18 37.645 -10.168 25.284 0.0186 1.2000 H

ATOM 320 HD12 ILE 18 36.168 -9.844 24.351 0.0186 1.2000 H

ATOM 321 HD13 ILE 18 36.696 -11.483 24.749 0.0186 1.2000 H

ATOM 322 C ILE 18 37.091 -11.455 19.692 0.5973 1.7000 C

ATOM 323 O ILE 18 37.658 -12.448 19.258 -0.5679 1.5000 O

ATOM 324 N GLN 19 35.931 -11.031 19.192 -0.4157 1.5500 N

ATOM 325 H GLN 19 35.477 -10.203 19.539 0.2719 1.3000 H

ATOM 326 CA GLN 19 35.301 -11.832 18.149 -0.0031 1.7000 C

ATOM 327 HA GLN 19 35.137 -12.838 18.531 0.0850 1.2000 H

ATOM 328 CB GLN 19 33.949 -11.254 17.737 -0.0036 1.7000 C

ATOM 329 HB2 GLN 19 33.352 -11.126 18.636 0.0171 1.2000 H

ATOM 330 HB3 GLN 19 34.104 -10.268 17.295 0.0171 1.2000 H

ATOM 331 CG GLN 19 33.140 -12.137 16.737 -0.0645 1.7000 C

ATOM 332 HG2 GLN 19 33.699 -12.240 15.806 0.0352 1.2000 H

ATOM 333 HG3 GLN 19 33.015 -13.134 17.159 0.0352 1.2000 H

ATOM 334 CD GLN 19 31.754 -11.544 16.402 0.6951 1.7000 C

ATOM 335 OE1 GLN 19 31.691 -10.366 16.035 -0.6086 1.5000 O

ATOM 336 NE2 GLN 19 30.655 -12.321 16.519 -0.9407 1.5500 N

ATOM 337 HE21 GLN 19 30.743 -13.286 16.807 0.4251 1.3000 H

ATOM 338 HE22 GLN 19 29.753 -11.943 16.271 0.4251 1.3000 H

ATOM 339 C GLN 19 36.239 -11.943 16.961 0.5973 1.7000 C

ATOM 340 O GLN 19 36.403 -13.024 16.396 -0.5679 1.5000 O

ATOM 341 N ALA 20 36.918 -10.844 16.615 -0.4157 1.5500 N

ATOM 342 H ALA 20 36.778 -9.979 17.117 0.2719 1.3000 H

ATOM 343 CA ALA 20 37.860 -10.860 15.503 0.0337 1.7000 C

ATOM 344 HA ALA 20 37.321 -11.125 14.592 0.0823 1.2000 H

ATOM 345 CB ALA 20 38.470 -9.482 15.319 -0.1825 1.7000 C

ATOM 346 HB1 ALA 20 39.136 -9.489 14.458 0.0603 1.2000 H

ATOM 347 HB2 ALA 20 37.684 -8.750 15.145 0.0603 1.2000 H

ATOM 348 HB3 ALA 20 39.042 -9.190 16.195 0.0603 1.2000 H

ATOM 349 C ALA 20 38.971 -11.894 15.729 0.5973 1.7000 C

ATOM 350 O ALA 20 39.444 -12.531 14.785 -0.5679 1.5000 O

ATOM 351 N MET 21 39.371 -12.072 16.989 -0.4157 1.5500 N

ATOM 352 H MET 21 38.937 -11.533 17.723 0.2719 1.3000 H

ATOM 353 CA MET 21 40.428 -13.002 17.367 -0.0237 1.7000 C

ATOM 354 HA MET 21 41.138 -13.060 16.541 0.0880 1.2000 H

ATOM 355 CB MET 21 41.190 -12.446 18.569 0.0342 1.7000 C

ATOM 356 HB2 MET 21 40.486 -12.239 19.375 0.0241 1.2000 H

ATOM 357 HB3 MET 21 41.901 -13.193 18.925 0.0241 1.2000 H

ATOM 358 CG MET 21 41.993 -11.179 18.250 0.0018 1.7000 C

ATOM 359 HG2 MET 21 42.639 -11.392 17.398 0.0440 1.2000 H

ATOM 360 HG3 MET 21 41.322 -10.369 17.968 0.0440 1.2000 H

ATOM 361 SD MET 21 43.039 -10.638 19.604 -0.2737 1.8000 S

ATOM 362 CE MET 21 41.854 -10.020 20.756 -0.0536 1.7000 C

ATOM 363 HE1 MET 21 42.378 -9.601 21.614 0.0684 1.2000 H

ATOM 364 HE2 MET 21 41.260 -9.241 20.282 0.0684 1.2000 H

ATOM 365 HE3 MET 21 41.203 -10.827 21.089 0.0684 1.2000 H

ATOM 366 C MET 21 39.973 -14.448 17.639 0.5973 1.7000 C

ATOM 367 O MET 21 40.808 -15.279 18.011 -0.5679 1.5000 O

ATOM 368 N ILE 22 38.681 -14.771 17.449 -0.4157 1.5500 N

ATOM 369 H ILE 22 38.018 -14.071 17.147 0.2719 1.3000 H

ATOM 370 CA ILE 22 38.209 -16.145 17.679 -0.0597 1.7000 C

ATOM 371 HA ILE 22 39.046 -16.793 17.943 0.0869 1.2000 H

ATOM 372 CB ILE 22 37.219 -16.215 18.845 0.1303 1.7000 C

ATOM 373 HB ILE 22 36.343 -15.616 18.589 0.0187 1.2000 H

ATOM 374 CG2 ILE 22 36.780 -17.673 19.086 -0.3204 1.7000 C

ATOM 375 HG21 ILE 22 35.896 -17.687 19.721 0.0882 1.2000 H

ATOM 376 HG22 ILE 22 36.544 -18.310 18.244 0.0882 1.2000 H

ATOM 377 HG23 ILE 22 37.585 -18.193 19.600 0.0882 1.2000 H

ATOM 378 CG1 ILE 22 37.805 -15.619 20.064 -0.0430 1.7000 C

ATOM 379 HG12 ILE 22 38.666 -16.205 20.377 0.0236 1.2000 H

ATOM 380 HG13 ILE 22 38.183 -14.624 19.892 0.0236 1.2000 H

ATOM 381 CD1 ILE 22 36.830 -15.535 21.152 -0.0660 1.7000 C

ATOM 382 HD11 ILE 22 37.312 -15.077 22.009 0.0186 1.2000 H

ATOM 383 HD12 ILE 22 35.985 -14.927 20.845 0.0186 1.2000 H

ATOM 384 HD13 ILE 22 36.469 -16.511 21.461 0.0186 1.2000 H

ATOM 385 C ILE 22 37.481 -16.801 16.508 0.5973 1.7000 C

ATOM 386 O ILE 22 36.256 -16.899 16.543 -0.5679 1.5000 O

ATOM 387 N PRO 23 38.183 -17.298 15.479 -0.2548 1.5500 N

ATOM 388 CD PRO 23 39.637 -17.089 15.383 0.0192 1.7000 C

ATOM 389 HD2 PRO 23 40.164 -17.804 16.016 0.0391 1.2000 H

ATOM 390 HD3 PRO 23 39.934 -16.067 15.600 0.0391 1.2000 H

ATOM 391 CG PRO 23 39.904 -17.368 13.928 0.0189 1.7000 C

ATOM 392 HG2 PRO 23 40.914 -17.749 13.774 0.0213 1.2000 H

ATOM 393 HG3 PRO 23 39.758 -16.452 13.352 0.0213 1.2000 H

ATOM 394 CB PRO 23 38.869 -18.374 13.532 -0.0070 1.7000 C

ATOM 395 HB2 PRO 23 39.223 -19.367 13.816 0.0253 1.2000 H

ATOM 396 HB3 PRO 23 38.671 -18.343 12.460 0.0253 1.2000 H

ATOM 397 CA PRO 23 37.617 -18.002 14.343 -0.0266 1.7000 C

ATOM 398 HA PRO 23 36.991 -17.316 13.771 0.0641 1.2000 H

ATOM 399 C PRO 23 36.838 -19.282 14.671 0.5896 1.7000 C

ATOM 400 O PRO 23 36.009 -19.702 13.861 -0.5748 1.5000 O

ATOM 401 N LYS 24 37.116 -19.930 15.821 -0.3479 1.5500 N

ATOM 402 H LYS 24 37.776 -19.556 16.485 0.2747 1.3000 H

ATOM 403 CA LYS 24 36.434 -21.202 16.084 -0.2400 1.7000 C

ATOM 404 HA LYS 24 35.622 -21.349 15.372 0.1426 1.2000 H

ATOM 405 CB LYS 24 37.423 -22.351 15.849 -0.0094 1.7000 C

ATOM 406 HB2 LYS 24 38.306 -22.195 16.472 0.0362 1.2000 H

ATOM 407 HB3 LYS 24 36.951 -23.282 16.170 0.0362 1.2000 H

ATOM 408 CG LYS 24 37.867 -22.540 14.393 0.0187 1.7000 C

ATOM 409 HG2 LYS 24 36.986 -22.715 13.772 0.0103 1.2000 H

ATOM 410 HG3 LYS 24 38.368 -21.637 14.044 0.0103 1.2000 H

ATOM 411 CD LYS 24 38.824 -23.713 14.244 -0.0479 1.7000 C

ATOM 412 HD2 LYS 24 39.705 -23.538 14.865 0.0621 1.2000 H

ATOM 413 HD3 LYS 24 38.330 -24.627 14.580 0.0621 1.2000 H

ATOM 414 CE LYS 24 39.262 -23.886 12.793 -0.0143 1.7000 C

ATOM 415 HE2 LYS 24 38.380 -24.083 12.179 0.1135 1.2000 H

ATOM 416 HE3 LYS 24 39.725 -22.957 12.451 0.1135 1.2000 H

ATOM 417 NZ LYS 24 40.233 -25.004 12.634 -0.3854 1.5500 N

ATOM 418 HZ1 LYS 24 41.056 -24.827 13.193 0.3400 1.3000 H

ATOM 419 HZ2 LYS 24 39.812 -25.870 12.938 0.3400 1.3000 H

ATOM 420 HZ3 LYS 24 40.501 -25.089 11.664 0.3400 1.3000 H

ATOM 421 C LYS 24 35.771 -21.454 17.447 0.7341 1.7000 C

ATOM 422 O LYS 24 34.585 -21.778 17.486 -0.5894 1.5000 O

ATOM 423 N GLY 25 36.513 -21.386 18.564 -0.4157 1.5500 N

ATOM 424 H GLY 25 37.498 -21.173 18.544 0.2719 1.3000 H

ATOM 425 CA GLY 25 35.858 -21.826 19.803 -0.0252 1.7000 C

ATOM 426 HA2 GLY 25 34.875 -21.360 19.844 0.0698 1.2000 H

ATOM 427 HA3 GLY 25 35.703 -22.904 19.737 0.0698 1.2000 H

ATOM 428 C GLY 25 36.507 -21.530 21.166 0.5973 1.7000 C

ATOM 429 O GLY 25 36.252 -22.267 22.117 -0.5679 1.5000 O

ATOM 430 N ALA 26 37.351 -20.513 21.293 -0.4157 1.5500 N

ATOM 431 H ALA 26 37.546 -19.888 20.525 0.2719 1.3000 H

ATOM 432 CA ALA 26 37.976 -20.270 22.605 0.0337 1.7000 C

ATOM 433 HA ALA 26 37.366 -20.691 23.406 0.0823 1.2000 H

ATOM 434 CB ALA 26 39.341 -20.926 22.664 -0.1825 1.7000 C

ATOM 435 HB1 ALA 26 39.792 -20.756 23.642 0.0603 1.2000 H

ATOM 436 HB2 ALA 26 39.240 -22.002 22.507 0.0603 1.2000 H

ATOM 437 HB3 ALA 26 39.990 -20.508 21.892 0.0603 1.2000 H

ATOM 438 C ALA 26 38.107 -18.789 22.882 0.5973 1.7000 C

ATOM 439 O ALA 26 38.419 -18.024 21.984 -0.5679 1.5000 O

ATOM 440 N LEU 27 37.928 -18.407 24.149 -0.4157 1.5500 N

ATOM 441 H LEU 27 37.695 -19.100 24.845 0.2719 1.3000 H

ATOM 442 CA LEU 27 37.925 -17.002 24.557 -0.0518 1.7000 C

ATOM 443 HA LEU 27 37.961 -16.411 23.645 0.0922 1.2000 H

ATOM 444 CB LEU 27 36.575 -16.719 25.205 -0.1102 1.7000 C

ATOM 445 HB2 LEU 27 35.809 -16.923 24.470 0.0457 1.2000 H

ATOM 446 HB3 LEU 27 36.436 -17.434 26.019 0.0457 1.2000 H

ATOM 447 CG LEU 27 36.379 -15.381 25.754 0.3531 1.7000 C

ATOM 448 HG LEU 27 37.013 -15.210 26.628 -0.0361 1.2000 H

ATOM 449 CD1 LEU 27 36.623 -14.433 24.752 -0.4121 1.7000 C

ATOM 450 HD11 LEU 27 36.427 -13.459 25.183 0.1000 1.2000 H

ATOM 451 HD12 LEU 27 37.608 -14.382 24.328 0.1000 1.2000 H

ATOM 452 HD13 LEU 27 35.935 -14.576 23.915 0.1000 1.2000 H

ATOM 453 CD2 LEU 27 34.948 -15.206 26.134 -0.4121 1.7000 C

ATOM 454 HD21 LEU 27 34.812 -14.253 26.645 0.1000 1.2000 H

ATOM 455 HD22 LEU 27 34.288 -15.226 25.266 0.1000 1.2000 H

ATOM 456 HD23 LEU 27 34.653 -16.004 26.815 0.1000 1.2000 H

ATOM 457 C LEU 27 39.038 -16.432 25.439 0.5973 1.7000 C

ATOM 458 O LEU 27 39.426 -15.276 25.258 -0.5679 1.5000 O

ATOM 459 N ALA 28 39.531 -17.182 26.416 -0.4157 1.5500 N

ATOM 460 H ALA 28 39.197 -18.122 26.564 0.2719 1.3000 H

ATOM 461 CA ALA 28 40.446 -16.581 27.395 0.0337 1.7000 C

ATOM 462 HA ALA 28 39.897 -15.820 27.953 0.0823 1.2000 H

ATOM 463 CB ALA 28 40.911 -17.639 28.374 -0.1825 1.7000 C

ATOM 464 HB1 ALA 28 41.554 -17.191 29.130 0.0603 1.2000 H

ATOM 465 HB2 ALA 28 40.053 -18.088 28.873 0.0603 1.2000 H

ATOM 466 HB3 ALA 28 41.469 -18.420 27.858 0.0603 1.2000 H

ATOM 467 C ALA 28 41.676 -15.918 26.768 0.5973 1.7000 C

ATOM 468 O ALA 28 42.123 -14.871 27.245 -0.5679 1.5000 O

ATOM 469 N VAL 29 42.216 -16.497 25.698 -0.4157 1.5500 N

ATOM 470 H VAL 29 41.830 -17.349 25.320 0.2719 1.3000 H

ATOM 471 CA VAL 29 43.388 -15.901 25.075 -0.0875 1.7000 C

ATOM 472 HA VAL 29 44.152 -15.739 25.837 0.0969 1.2000 H

ATOM 473 CB VAL 29 43.962 -16.813 23.991 0.2985 1.7000 C

ATOM 474 HB VAL 29 43.180 -17.081 23.278 -0.0297 1.2000 H

ATOM 475 CG1 VAL 29 45.066 -16.072 23.246 -0.3192 1.7000 C

ATOM 476 HG11 VAL 29 45.603 -16.770 22.601 0.0791 1.2000 H

ATOM 477 HG12 VAL 29 44.651 -15.300 22.599 0.0791 1.2000 H

ATOM 478 HG13 VAL 29 45.774 -15.629 23.948 0.0791 1.2000 H

ATOM 479 CG2 VAL 29 44.485 -18.083 24.635 -0.3192 1.7000 C

ATOM 480 HG21 VAL 29 44.929 -18.726 23.876 0.0791 1.2000 H

ATOM 481 HG22 VAL 29 45.243 -17.848 25.383 0.0791 1.2000 H

ATOM 482 HG23 VAL 29 43.677 -18.638 25.109 0.0791 1.2000 H

ATOM 483 C VAL 29 43.045 -14.563 24.456 0.5973 1.7000 C

ATOM 484 O VAL 29 43.773 -13.587 24.631 -0.5679 1.5000 O

ATOM 485 N ALA 30 41.928 -14.517 23.731 -0.4157 1.5500 N

ATOM 486 H ALA 30 41.357 -15.342 23.626 0.2719 1.3000 H

ATOM 487 CA ALA 30 41.501 -13.297 23.069 0.0337 1.7000 C

ATOM 488 HA ALA 30 42.280 -12.980 22.376 0.0823 1.2000 H

ATOM 489 CB ALA 30 40.246 -13.570 22.280 -0.1825 1.7000 C

ATOM 490 HB1 ALA 30 39.919 -12.658 21.788 0.0603 1.2000 H

ATOM 491 HB2 ALA 30 40.448 -14.323 21.517 0.0603 1.2000 H

ATOM 492 HB3 ALA 30 39.464 -13.936 22.942 0.0603 1.2000 H

ATOM 493 C ALA 30 41.287 -12.191 24.096 0.5973 1.7000 C

ATOM 494 O ALA 30 41.656 -11.035 23.863 -0.5679 1.5000 O

ATOM 495 N VAL 31 40.752 -12.556 25.265 -0.4157 1.5500 N

ATOM 496 H VAL 31 40.471 -13.513 25.429 0.2719 1.3000 H

ATOM 497 CA VAL 31 40.568 -11.566 26.313 -0.0875 1.7000 C

ATOM 498 HA VAL 31 39.944 -10.751 25.943 0.0969 1.2000 H

ATOM 499 CB VAL 31 39.936 -12.170 27.565 0.2985 1.7000 C

ATOM 500 HB VAL 31 40.493 -13.051 27.878 -0.0297 1.2000 H

ATOM 501 CG1 VAL 31 40.000 -11.146 28.685 -0.3192 1.7000 C

ATOM 502 HG11 VAL 31 39.526 -11.583 29.556 0.0791 1.2000 H

ATOM 503 HG12 VAL 31 41.017 -10.936 29.021 0.0791 1.2000 H

ATOM 504 HG13 VAL 31 39.493 -10.222 28.413 0.0791 1.2000 H

ATOM 505 CG2 VAL 31 38.516 -12.594 27.286 -0.3192 1.7000 C

ATOM 506 HG21 VAL 31 38.481 -13.253 26.427 0.0791 1.2000 H

ATOM 507 HG22 VAL 31 38.116 -13.126 28.150 0.0791 1.2000 H

ATOM 508 HG23 VAL 31 37.895 -11.719 27.093 0.0791 1.2000 H

ATOM 509 C VAL 31 41.889 -11.012 26.758 0.5973 1.7000 C

ATOM 510 O VAL 31 42.059 -9.794 26.831 -0.5679 1.5000 O

ATOM 511 N ALA 32 42.841 -11.910 27.029 -0.4157 1.5500 N

ATOM 512 H ALA 32 42.650 -12.898 26.947 0.2719 1.3000 H

ATOM 513 CA ALA 32 44.142 -11.485 27.497 0.0337 1.7000 C

ATOM 514 HA ALA 32 44.017 -10.945 28.438 0.0823 1.2000 H

ATOM 515 CB ALA 32 45.029 -12.690 27.744 -0.1825 1.7000 C

ATOM 516 HB1 ALA 32 45.990 -12.359 28.138 0.0603 1.2000 H

ATOM 517 HB2 ALA 32 44.557 -13.348 28.475 0.0603 1.2000 H

ATOM 518 HB3 ALA 32 45.194 -13.243 26.820 0.0603 1.2000 H

ATOM 519 C ALA 32 44.797 -10.570 26.492 0.5973 1.7000 C

ATOM 520 O ALA 32 45.382 -9.554 26.863 -0.5679 1.5000 O

ATOM 521 N GLN 33 44.644 -10.876 25.208 -0.4157 1.5500 N

ATOM 522 H GLN 33 44.122 -11.693 24.927 0.2719 1.3000 H

ATOM 523 CA GLN 33 45.260 -10.040 24.201 -0.0031 1.7000 C

ATOM 524 HA GLN 33 46.331 -9.972 24.408 0.0850 1.2000 H

ATOM 525 CB GLN 33 45.086 -10.695 22.829 -0.0036 1.7000 C

ATOM 526 HB2 GLN 33 44.038 -10.955 22.687 0.0171 1.2000 H

ATOM 527 HB3 GLN 33 45.365 -9.977 22.055 0.0171 1.2000 H

ATOM 528 CG GLN 33 45.952 -11.955 22.650 -0.0645 1.7000 C

ATOM 529 HG2 GLN 33 46.998 -11.645 22.654 0.0352 1.2000 H

ATOM 530 HG3 GLN 33 45.819 -12.626 23.496 0.0352 1.2000 H

ATOM 531 CD GLN 33 45.662 -12.747 21.375 0.6951 1.7000 C

ATOM 532 OE1 GLN 33 44.520 -13.078 21.049 -0.6086 1.5000 O

ATOM 533 NE2 GLN 33 46.724 -13.056 20.631 -0.9407 1.5500 N

ATOM 534 HE21 GLN 33 47.646 -12.768 20.916 0.4251 1.3000 H

ATOM 535 HE22 GLN 33 46.584 -13.555 19.766 0.4251 1.3000 H

ATOM 536 C GLN 33 44.687 -8.627 24.254 0.5973 1.7000 C

ATOM 537 O GLN 33 45.444 -7.653 24.253 -0.5679 1.5000 O

ATOM 538 N VAL 34 43.377 -8.490 24.429 -0.4157 1.5500 N

ATOM 539 H VAL 34 42.763 -9.292 24.479 0.2719 1.3000 H

ATOM 540 CA VAL 34 42.830 -7.144 24.503 -0.0875 1.7000 C

ATOM 541 HA VAL 34 43.157 -6.581 23.627 0.0969 1.2000 H

ATOM 542 CB VAL 34 41.313 -7.120 24.563 0.2985 1.7000 C

ATOM 543 HB VAL 34 40.971 -7.744 25.387 -0.0297 1.2000 H

ATOM 544 CG1 VAL 34 40.868 -5.653 24.826 -0.3192 1.7000 C

ATOM 545 HG11 VAL 34 39.810 -5.543 24.624 0.0791 1.2000 H

ATOM 546 HG12 VAL 34 41.005 -5.368 25.869 0.0791 1.2000 H

ATOM 547 HG13 VAL 34 41.405 -4.965 24.172 0.0791 1.2000 H

ATOM 548 CG2 VAL 34 40.748 -7.665 23.295 -0.3192 1.7000 C

ATOM 549 HG21 VAL 34 39.670 -7.521 23.280 0.0791 1.2000 H

ATOM 550 HG22 VAL 34 41.191 -7.157 22.439 0.0791 1.2000 H

ATOM 551 HG23 VAL 34 40.943 -8.730 23.221 0.0791 1.2000 H

ATOM 552 C VAL 34 43.280 -6.410 25.736 0.5973 1.7000 C

ATOM 553 O VAL 34 43.641 -5.231 25.671 -0.5679 1.5000 O

ATOM 554 N CYX 35 43.232 -7.103 26.867 -0.4157 1.5500 N

ATOM 555 H CYX 35 42.921 -8.065 26.863 0.2719 1.3000 H

ATOM 556 CA CYX 35 43.534 -6.477 28.142 0.0429 1.7000 C

ATOM 557 HA CYX 35 42.929 -5.579 28.243 0.0766 1.2000 H

ATOM 558 CB CYX 35 43.155 -7.424 29.275 -0.0790 1.7000 C

ATOM 559 HB2 CYX 35 43.723 -8.352 29.190 0.0910 1.2000 H

ATOM 560 HB3 CYX 35 43.422 -6.949 30.220 0.0910 1.2000 H

ATOM 561 SG CYX 35 41.362 -7.820 29.331 -0.1081 1.8000 S

ATOM 562 C CYX 35 45.003 -6.071 28.238 0.5973 1.7000 C

ATOM 563 O CYX 35 45.348 -5.142 28.967 -0.5679 1.5000 O

ATOM 564 N ARG 36 45.868 -6.731 27.461 -0.3479 1.5500 N

ATOM 565 H ARG 36 45.536 -7.495 26.888 0.2747 1.3000 H

ATOM 566 CA ARG 36 47.278 -6.364 27.368 -0.2637 1.7000 C

ATOM 567 HA ARG 36 47.650 -6.121 28.365 0.1560 1.2000 H

ATOM 568 CB ARG 36 48.080 -7.537 26.835 -0.0007 1.7000 C

ATOM 569 HB2 ARG 36 47.611 -7.901 25.920 0.0327 1.2000 H

ATOM 570 HB3 ARG 36 49.084 -7.192 26.581 0.0327 1.2000 H

ATOM 571 CG ARG 36 48.214 -8.690 27.834 0.0390 1.7000 C

ATOM 572 HG2 ARG 36 48.863 -8.360 28.646 0.0285 1.2000 H

ATOM 573 HG3 ARG 36 47.250 -8.932 28.276 0.0285 1.2000 H

ATOM 574 CD ARG 36 48.799 -9.904 27.227 0.0486 1.7000 C

ATOM 575 HD2 ARG 36 48.163 -10.216 26.397 0.0687 1.2000 H

ATOM 576 HD3 ARG 36 49.793 -9.670 26.843 0.0687 1.2000 H

ATOM 577 NE ARG 36 48.899 -10.991 28.194 -0.5295 1.5500 N

ATOM 578 HE ARG 36 48.743 -10.757 29.163 0.3456 1.3000 H

ATOM 579 CZ ARG 36 49.168 -12.272 27.882 0.8076 1.7000 C

ATOM 580 NH1 ARG 36 49.356 -12.618 26.629 -0.8627 1.5500 N

ATOM 581 HH11 ARG 36 49.325 -11.914 25.909 0.4478 1.3000 H

ATOM 582 HH12 ARG 36 49.554 -13.578 26.393 0.4478 1.3000 H

ATOM 583 NH2 ARG 36 49.240 -13.183 28.836 -0.8627 1.5500 N

ATOM 584 HH21 ARG 36 49.157 -12.907 29.802 0.4478 1.3000 H

ATOM 585 HH22 ARG 36 49.368 -14.154 28.601 0.4478 1.3000 H

ATOM 586 C ARG 36 47.473 -5.115 26.492 0.7341 1.7000 C

ATOM 587 O ARG 36 48.378 -4.315 26.737 -0.5894 1.5000 O

ATOM 588 N VAL 37 46.626 -4.955 25.466 -0.4157 1.5500 N

ATOM 589 H VAL 37 45.910 -5.650 25.309 0.2719 1.3000 H

ATOM 590 CA VAL 37 46.676 -3.804 24.559 -0.0875 1.7000 C

ATOM 591 HA VAL 37 47.718 -3.639 24.277 0.0969 1.2000 H

ATOM 592 CB VAL 37 45.877 -4.097 23.275 0.2985 1.7000 C

ATOM 593 HB VAL 37 44.877 -4.451 23.523 -0.0297 1.2000 H

ATOM 594 CG1 VAL 37 45.758 -2.844 22.425 -0.3192 1.7000 C

ATOM 595 HG11 VAL 37 45.366 -3.103 21.441 0.0791 1.2000 H

ATOM 596 HG12 VAL 37 45.065 -2.133 22.873 0.0791 1.2000 H

ATOM 597 HG13 VAL 37 46.734 -2.374 22.294 0.0791 1.2000 H

ATOM 598 CG2 VAL 37 46.601 -5.165 22.480 -0.3192 1.7000 C

ATOM 599 HG21 VAL 37 45.968 -5.505 21.656 0.0791 1.2000 H

ATOM 600 HG22 VAL 37 47.528 -4.762 22.071 0.0791 1.2000 H

ATOM 601 HG23 VAL 37 46.854 -6.023 23.095 0.0791 1.2000 H

ATOM 602 C VAL 37 46.168 -2.499 25.177 0.5973 1.7000 C

ATOM 603 O VAL 37 46.753 -1.433 24.967 -0.5679 1.5000 O

ATOM 604 N VAL 38 45.056 -2.568 25.895 -0.4157 1.5500 N

ATOM 605 H VAL 38 44.611 -3.461 26.057 0.2719 1.3000 H

ATOM 606 CA VAL 38 44.487 -1.370 26.505 -0.0875 1.7000 C

ATOM 607 HA VAL 38 44.381 -0.618 25.726 0.0969 1.2000 H

ATOM 608 CB VAL 38 43.090 -1.693 27.075 0.2985 1.7000 C

ATOM 609 HB VAL 38 42.657 -0.801 27.522 -0.0297 1.2000 H

ATOM 610 CG1 VAL 38 42.166 -2.157 25.969 -0.3192 1.7000 C

ATOM 611 HG11 VAL 38 41.191 -2.411 26.381 0.0791 1.2000 H

ATOM 612 HG12 VAL 38 42.032 -1.351 25.249 0.0791 1.2000 H

ATOM 613 HG13 VAL 38 42.551 -3.027 25.446 0.0791 1.2000 H

ATOM 614 CG2 VAL 38 43.241 -2.720 28.094 -0.3192 1.7000 C

ATOM 615 HG21 VAL 38 42.263 -2.952 28.516 0.0791 1.2000 H

ATOM 616 HG22 VAL 38 43.653 -3.633 27.683 0.0791 1.2000 H

ATOM 617 HG23 VAL 38 43.871 -2.376 28.909 0.0791 1.2000 H

ATOM 618 C VAL 38 45.446 -0.864 27.610 0.5973 1.7000 C

ATOM 619 O VAL 38 46.231 -1.650 28.134 -0.5679 1.5000 O

ATOM 620 N PRO 39 45.413 0.427 27.999 -0.2548 1.5500 N

ATOM 621 CD PRO 39 44.509 1.405 27.336 0.0192 1.7000 C

ATOM 622 HD2 PRO 39 43.482 1.265 27.670 0.0391 1.2000 H

ATOM 623 HD3 PRO 39 44.586 1.377 26.249 0.0391 1.2000 H

ATOM 624 CG PRO 39 45.045 2.723 27.849 0.0189 1.7000 C

ATOM 625 HG2 PRO 39 44.255 3.471 27.924 0.0213 1.2000 H

ATOM 626 HG3 PRO 39 45.833 3.075 27.181 0.0213 1.2000 H

ATOM 627 CB PRO 39 45.643 2.409 29.205 -0.0070 1.7000 C

ATOM 628 HB2 PRO 39 44.844 2.383 29.949 0.0253 1.2000 H

ATOM 629 HB3 PRO 39 46.395 3.145 29.490 0.0253 1.2000 H

ATOM 630 CA PRO 39 46.261 1.019 29.032 -0.0266 1.7000 C

ATOM 631 HA PRO 39 47.288 1.092 28.673 0.0641 1.2000 H

ATOM 632 C PRO 39 46.174 0.212 30.311 0.5896 1.7000 C

ATOM 633 O PRO 39 45.098 -0.234 30.676 -0.5748 1.5000 O

ATOM 634 N LEU 40 47.274 0.094 31.047 -0.4157 1.5500 N

ATOM 635 H LEU 40 48.140 0.497 30.721 0.2719 1.3000 H

ATOM 636 CA LEU 40 47.307 -0.739 32.259 -0.0518 1.7000 C

ATOM 637 HA LEU 40 47.423 -1.774 31.934 0.0922 1.2000 H

ATOM 638 CB LEU 40 48.515 -0.344 33.113 -0.1102 1.7000 C

ATOM 639 HB2 LEU 40 49.419 -0.479 32.513 0.0457 1.2000 H

ATOM 640 HB3 LEU 40 48.434 0.720 33.343 0.0457 1.2000 H

ATOM 641 CG LEU 40 48.671 -1.106 34.426 0.3531 1.7000 C

ATOM 642 HG LEU 40 47.780 -0.996 35.044 -0.0361 1.2000 H

ATOM 643 CD1 LEU 40 48.890 -2.577 34.127 -0.4121 1.7000 C

ATOM 644 HD11 LEU 40 49.095 -3.110 35.056 0.1000 1.2000 H

ATOM 645 HD12 LEU 40 48.000 -3.018 33.679 0.1000 1.2000 H

ATOM 646 HD13 LEU 40 49.738 -2.708 33.454 0.1000 1.2000 H

ATOM 647 CD2 LEU 40 49.833 -0.518 35.205 -0.4121 1.7000 C

ATOM 648 HD21 LEU 40 49.950 -1.053 36.148 0.1000 1.2000 H

ATOM 649 HD22 LEU 40 50.757 -0.603 34.632 0.1000 1.2000 H

ATOM 650 HD23 LEU 40 49.640 0.533 35.424 0.1000 1.2000 H

ATOM 651 C LEU 40 46.071 -0.656 33.155 0.5973 1.7000 C

ATOM 652 O LEU 40 45.542 -1.691 33.573 -0.5679 1.5000 O

ATOM 653 N VAL 41 45.597 0.548 33.451 -0.4157 1.5500 N

ATOM 654 H VAL 41 46.049 1.381 33.104 0.2719 1.3000 H

ATOM 655 CA VAL 41 44.454 0.676 34.345 -0.0875 1.7000 C

ATOM 656 HA VAL 41 44.636 0.084 35.245 0.0969 1.2000 H

ATOM 657 CB VAL 41 44.314 2.150 34.758 0.2985 1.7000 C

ATOM 658 HB VAL 41 43.457 2.257 35.424 -0.0297 1.2000 H

ATOM 659 CG1 VAL 41 45.581 2.575 35.493 -0.3192 1.7000 C

ATOM 660 HG11 VAL 41 45.298 3.025 36.446 0.0791 1.2000 H

ATOM 661 HG12 VAL 41 46.237 1.730 35.712 0.0791 1.2000 H

ATOM 662 HG13 VAL 41 46.143 3.314 34.919 0.0791 1.2000 H

ATOM 663 CG2 VAL 41 44.109 3.033 33.511 -0.3192 1.7000 C

ATOM 664 HG21 VAL 41 44.067 4.078 33.821 0.0791 1.2000 H

ATOM 665 HG22 VAL 41 44.922 2.929 32.793 0.0791 1.2000 H

ATOM 666 HG23 VAL 41 43.160 2.802 33.027 0.0791 1.2000 H

ATOM 667 C VAL 41 43.172 0.153 33.691 0.5973 1.7000 C

ATOM 668 O VAL 41 42.260 -0.347 34.367 -0.5679 1.5000 O

ATOM 669 N ALA 42 43.132 0.231 32.363 -0.4157 1.5500 N

ATOM 670 H ALA 42 43.915 0.629 31.868 0.2719 1.3000 H

ATOM 671 CA ALA 42 42.028 -0.259 31.580 0.0337 1.7000 C

ATOM 672 HA ALA 42 41.093 0.016 32.070 0.0823 1.2000 H

ATOM 673 CB ALA 42 42.040 0.348 30.194 -0.1825 1.7000 C

ATOM 674 HB1 ALA 42 41.121 0.101 29.674 0.0603 1.2000 H

ATOM 675 HB2 ALA 42 42.109 1.433 30.269 0.0603 1.2000 H

ATOM 676 HB3 ALA 42 42.882 -0.023 29.619 0.0603 1.2000 H

ATOM 677 C ALA 42 42.139 -1.764 31.538 0.5973 1.7000 C

ATOM 678 O ALA 42 41.135 -2.458 31.557 -0.5679 1.5000 O

ATOM 679 N GLY 43 43.382 -2.259 31.511 -0.4157 1.5500 N

ATOM 680 H GLY 43 44.165 -1.622 31.510 0.2719 1.3000 H

ATOM 681 CA GLY 43 43.685 -3.689 31.498 -0.0252 1.7000 C

ATOM 682 HA2 GLY 43 43.239 -4.160 30.622 0.0698 1.2000 H

ATOM 683 HA3 GLY 43 44.767 -3.821 31.469 0.0698 1.2000 H

ATOM 684 C GLY 43 43.137 -4.324 32.766 0.5973 1.7000 C

ATOM 685 O GLY 43 42.559 -5.413 32.741 -0.5679 1.5000 O

ATOM 686 N GLY 44 43.268 -3.599 33.879 -0.4157 1.5500 N

ATOM 687 H GLY 44 43.759 -2.717 33.841 0.2719 1.3000 H

ATOM 688 CA GLY 44 42.730 -4.028 35.164 -0.0252 1.7000 C

ATOM 689 HA2 GLY 44 43.206 -4.960 35.470 0.0698 1.2000 H

ATOM 690 HA3 GLY 44 42.932 -3.258 35.910 0.0698 1.2000 H

ATOM 691 C GLY 44 41.222 -4.233 35.046 0.5973 1.7000 C

ATOM 692 O GLY 44 40.701 -5.318 35.337 -0.5679 1.5000 O

ATOM 693 N ILE 45 40.520 -3.189 34.589 -0.4157 1.5500 N

ATOM 694 H ILE 45 40.986 -2.323 34.358 0.2719 1.3000 H

ATOM 695 CA ILE 45 39.068 -3.275 34.451 -0.0597 1.7000 C

ATOM 696 HA ILE 45 38.635 -3.533 35.420 0.0869 1.2000 H

ATOM 697 CB ILE 45 38.460 -1.944 33.973 0.1303 1.7000 C

ATOM 698 HB ILE 45 39.003 -1.624 33.085 0.0187 1.2000 H

ATOM 699 CG2 ILE 45 36.992 -2.149 33.612 -0.3204 1.7000 C

ATOM 700 HG21 ILE 45 36.490 -1.199 33.436 0.0882 1.2000 H

ATOM 701 HG22 ILE 45 36.896 -2.724 32.693 0.0882 1.2000 H

ATOM 702 HG23 ILE 45 36.473 -2.663 34.421 0.0882 1.2000 H

ATOM 703 CG1 ILE 45 38.587 -0.875 35.014 -0.0430 1.7000 C

ATOM 704 HG12 ILE 45 37.937 -1.099 35.862 0.0236 1.2000 H

ATOM 705 HG13 ILE 45 39.613 -0.840 35.382 0.0236 1.2000 H

ATOM 706 CD1 ILE 45 38.252 0.488 34.458 -0.0660 1.7000 C

ATOM 707 HD11 ILE 45 38.061 0.465 33.384 0.0186 1.2000 H

ATOM 708 HD12 ILE 45 37.379 0.889 34.967 0.0186 1.2000 H

ATOM 709 HD13 ILE 45 39.089 1.164 34.636 0.0186 1.2000 H

ATOM 710 C ILE 45 38.684 -4.355 33.449 0.5973 1.7000 C

ATOM 711 O ILE 45 37.807 -5.168 33.721 -0.5679 1.5000 O

ATOM 712 N CYX 46 39.371 -4.376 32.317 -0.4157 1.5500 N

ATOM 713 H CYX 46 40.116 -3.709 32.210 0.2719 1.3000 H

ATOM 714 CA CYX 46 39.147 -5.294 31.216 0.0429 1.7000 C

ATOM 715 HA CYX 46 38.185 -5.078 30.754 0.0766 1.2000 H

ATOM 716 CB CYX 46 40.258 -5.066 30.187 -0.0790 1.7000 C

ATOM 717 HB2 CYX 46 40.169 -4.042 29.827 0.0910 1.2000 H

ATOM 718 HB3 CYX 46 41.227 -5.159 30.670 0.0910 1.2000 H

ATOM 719 SG CYX 46 40.270 -6.095 28.776 -0.1081 1.8000 S

ATOM 720 C CYX 46 39.168 -6.735 31.667 0.5973 1.7000 C

ATOM 721 O CYX 46 38.242 -7.505 31.378 -0.5679 1.5000 O

ATOM 722 N GLN 47 40.217 -7.104 32.393 -0.4157 1.5500 N

ATOM 723 H GLN 47 40.947 -6.441 32.614 0.2719 1.3000 H

ATOM 724 CA GLN 47 40.356 -8.466 32.847 -0.0031 1.7000 C

ATOM 725 HA GLN 47 40.263 -9.138 31.991 0.0850 1.2000 H

ATOM 726 CB GLN 47 41.753 -8.630 33.449 -0.0036 1.7000 C

ATOM 727 HB2 GLN 47 42.483 -8.271 32.721 0.0171 1.2000 H

ATOM 728 HB3 GLN 47 41.830 -7.981 34.324 0.0171 1.2000 H

ATOM 729 CG GLN 47 42.179 -10.035 33.873 -0.0645 1.7000 C

ATOM 730 HG2 GLN 47 43.161 -9.978 34.344 0.0352 1.2000 H

ATOM 731 HG3 GLN 47 41.476 -10.392 34.627 0.0352 1.2000 H

ATOM 732 CD GLN 47 42.260 -11.039 32.723 0.6951 1.7000 C

ATOM 733 OE1 GLN 47 42.805 -10.758 31.648 -0.6086 1.5000 O

ATOM 734 NE2 GLN 47 41.768 -12.246 32.980 -0.9407 1.5500 N

ATOM 735 HE21 GLN 47 41.396 -12.448 33.901 0.4251 1.3000 H

ATOM 736 HE22 GLN 47 41.790 -12.961 32.272 0.4251 1.3000 H

ATOM 737 C GLN 47 39.259 -8.784 33.846 0.5973 1.7000 C

ATOM 738 O GLN 47 38.655 -9.853 33.784 -0.5679 1.5000 O

ATOM 739 N CYS 48 38.915 -7.825 34.710 -0.4157 1.5500 N

ATOM 740 H CYS 48 39.413 -6.945 34.727 0.2719 1.3000 H

ATOM 741 CA CYS 48 37.867 -8.071 35.692 0.0213 1.7000 C

ATOM 742 HA CYS 48 38.106 -8.975 36.257 0.1124 1.2000 H

ATOM 743 CB CYS 48 37.778 -6.895 36.659 -0.1231 1.7000 C

ATOM 744 HB2 CYS 48 37.636 -5.968 36.105 0.1112 1.2000 H

ATOM 745 HB3 CYS 48 36.909 -7.043 37.301 0.1112 1.2000 H

ATOM 746 SG CYS 48 39.226 -6.723 37.724 -0.3119 1.8000 S

ATOM 747 HG CYS 48 40.095 -6.473 36.752 0.1933 1.2000 H

ATOM 748 C CYS 48 36.511 -8.265 35.007 0.5973 1.7000 C

ATOM 749 O CYS 48 35.714 -9.133 35.393 -0.5679 1.5000 O

ATOM 750 N LEU 49 36.259 -7.483 33.951 -0.4157 1.5500 N

ATOM 751 H LEU 49 36.949 -6.815 33.638 0.2719 1.3000 H

ATOM 752 CA LEU 49 35.001 -7.592 33.235 -0.0518 1.7000 C

ATOM 753 HA LEU 49 34.165 -7.435 33.918 0.0922 1.2000 H

ATOM 754 CB LEU 49 34.950 -6.568 32.080 -0.1102 1.7000 C

ATOM 755 HB2 LEU 49 35.838 -6.711 31.466 0.0457 1.2000 H

ATOM 756 HB3 LEU 49 34.104 -6.821 31.446 0.0457 1.2000 H

ATOM 757 CG LEU 49 34.856 -5.061 32.417 0.3531 1.7000 C

ATOM 758 HG LEU 49 35.627 -4.773 33.111 -0.0361 1.2000 H

ATOM 759 CD1 LEU 49 35.024 -4.261 31.123 -0.4121 1.7000 C

ATOM 760 HD11 LEU 49 34.950 -3.196 31.341 0.1000 1.2000 H

ATOM 761 HD12 LEU 49 36.001 -4.461 30.686 0.1000 1.2000 H

ATOM 762 HD13 LEU 49 34.251 -4.528 30.402 0.1000 1.2000 H

ATOM 763 CD2 LEU 49 33.573 -4.733 33.054 -0.4121 1.7000 C

ATOM 764 HD21 LEU 49 33.546 -3.666 33.254 0.1000 1.2000 H

ATOM 765 HD22 LEU 49 32.755 -4.966 32.384 0.1000 1.2000 H

ATOM 766 HD23 LEU 49 33.453 -5.274 33.992 0.1000 1.2000 H

ATOM 767 C LEU 49 34.897 -8.978 32.625 0.5973 1.7000 C

ATOM 768 O LEU 49 33.852 -9.627 32.699 -0.5679 1.5000 O

ATOM 769 N ALA 50 35.998 -9.442 32.039 -0.4157 1.5500 N

ATOM 770 H ALA 50 36.840 -8.882 32.031 0.2719 1.3000 H

ATOM 771 CA ALA 50 36.030 -10.744 31.408 0.0337 1.7000 C

ATOM 772 HA ALA 50 35.209 -10.805 30.692 0.0823 1.2000 H

ATOM 773 CB ALA 50 37.315 -10.886 30.656 -0.1825 1.7000 C

ATOM 774 HB1 ALA 50 37.323 -11.849 30.147 0.0603 1.2000 H

ATOM 775 HB2 ALA 50 37.402 -10.093 29.914 0.0603 1.2000 H

ATOM 776 HB3 ALA 50 38.164 -10.833 31.339 0.0603 1.2000 H

ATOM 777 C ALA 50 35.891 -11.897 32.397 0.5973 1.7000 C

ATOM 778 O ALA 50 35.126 -12.841 32.167 -0.5679 1.5000 O

ATOM 779 N GLU 51 36.549 -11.805 33.544 -0.5163 1.5500 N

ATOM 780 H GLU 51 37.161 -11.026 33.740 0.2936 1.3000 H

ATOM 781 CA GLU 51 36.457 -12.900 34.496 0.0397 1.7000 C

ATOM 782 HA GLU 51 36.757 -13.819 33.990 0.1105 1.2000 H

ATOM 783 CB GLU 51 37.420 -12.687 35.660 0.0560 1.7000 C

ATOM 784 HB2 GLU 51 37.276 -11.686 36.070 -0.0173 1.2000 H

ATOM 785 HB3 GLU 51 37.203 -13.418 36.440 -0.0173 1.2000 H

ATOM 786 CG GLU 51 38.874 -12.861 35.227 0.0136 1.7000 C

ATOM 787 HG2 GLU 51 38.998 -13.888 34.879 -0.0425 1.2000 H

ATOM 788 HG3 GLU 51 39.086 -12.215 34.379 -0.0425 1.2000 H

ATOM 789 CD GLU 51 39.897 -12.609 36.278 0.8054 1.7000 C

ATOM 790 OE1 GLU 51 39.560 -12.315 37.405 -0.8188 1.5000 O

ATOM 791 OE2 GLU 51 41.052 -12.688 35.922 -0.8188 1.5000 O

ATOM 792 C GLU 51 35.021 -13.099 34.958 0.5366 1.7000 C

ATOM 793 O GLU 51 34.588 -14.230 35.188 -0.5819 1.5000 O

ATOM 794 N ARG 52 34.270 -12.008 35.074 -0.3479 1.5500 N

ATOM 795 H ARG 52 34.666 -11.096 34.891 0.2747 1.3000 H

ATOM 796 CA ARG 52 32.880 -12.112 35.476 -0.2637 1.7000 C

ATOM 797 HA ARG 52 32.778 -12.896 36.229 0.1560 1.2000 H

ATOM 798 CB ARG 52 32.430 -10.807 36.103 -0.0007 1.7000 C

ATOM 799 HB2 ARG 52 32.655 -9.993 35.412 0.0327 1.2000 H

ATOM 800 HB3 ARG 52 31.346 -10.854 36.221 0.0327 1.2000 H

ATOM 801 CG ARG 52 33.034 -10.483 37.451 0.0390 1.7000 C

ATOM 802 HG2 ARG 52 32.766 -11.262 38.166 0.0285 1.2000 H

ATOM 803 HG3 ARG 52 34.121 -10.460 37.362 0.0285 1.2000 H

ATOM 804 CD ARG 52 32.577 -9.147 37.961 0.0486 1.7000 C

ATOM 805 HD2 ARG 52 33.112 -8.915 38.883 0.0687 1.2000 H

ATOM 806 HD3 ARG 52 32.831 -8.380 37.226 0.0687 1.2000 H

ATOM 807 NE ARG 52 31.130 -9.113 38.204 -0.5295 1.5500 N

ATOM 808 HE ARG 52 30.558 -8.744 37.459 0.3456 1.3000 H

ATOM 809 CZ ARG 52 30.513 -9.545 39.302 0.8076 1.7000 C

ATOM 810 NH1 ARG 52 31.180 -10.057 40.317 -0.8627 1.5500 N

ATOM 811 HH11 ARG 52 32.183 -10.151 40.260 0.4478 1.3000 H

ATOM 812 HH12 ARG 52 30.692 -10.359 41.145 0.4478 1.3000 H

ATOM 813 NH2 ARG 52 29.210 -9.440 39.320 -0.8627 1.5500 N

ATOM 814 HH21 ARG 52 28.737 -8.975 38.562 0.4478 1.3000 H

ATOM 815 HH22 ARG 52 28.684 -9.824 40.089 0.4478 1.3000 H

ATOM 816 C ARG 52 31.908 -12.448 34.326 0.7341 1.7000 C

ATOM 817 O ARG 52 30.919 -13.154 34.547 -0.5894 1.5000 O

ATOM 818 N TYR 53 32.165 -11.934 33.109 -0.4157 1.5500 N

ATOM 819 H TYR 53 32.985 -11.363 32.967 0.2719 1.3000 H

ATOM 820 CA TYR 53 31.188 -12.065 32.023 -0.0014 1.7000 C

ATOM 821 HA TYR 53 30.317 -12.613 32.379 0.0876 1.2000 H

ATOM 822 CB TYR 53 30.717 -10.664 31.717 -0.0152 1.7000 C

ATOM 823 HB2 TYR 53 31.545 -10.076 31.318 0.0295 1.2000 H

ATOM 824 HB3 TYR 53 29.938 -10.702 30.962 0.0295 1.2000 H

ATOM 825 CG TYR 53 30.154 -10.014 32.977 -0.0011 1.7000 C

ATOM 826 CD1 TYR 53 30.776 -8.913 33.510 -0.1906 1.7000 C

ATOM 827 HD1 TYR 53 31.648 -8.507 33.051 0.1699 1.2000 H

ATOM 828 CE1 TYR 53 30.308 -8.329 34.645 -0.2341 1.7000 C

ATOM 829 HE1 TYR 53 30.807 -7.463 35.054 0.1656 1.2000 H

ATOM 830 CZ TYR 53 29.196 -8.850 35.276 0.3226 1.7000 C

ATOM 831 OH TYR 53 28.764 -8.256 36.433 -0.5579 1.5000 O

ATOM 832 HH TYR 53 27.836 -8.440 36.603 0.3992 1.2000 H

ATOM 833 CE2 TYR 53 28.557 -9.958 34.761 -0.2341 1.7000 C

ATOM 834 HE2 TYR 53 27.696 -10.375 35.262 0.1656 1.2000 H

ATOM 835 CD2 TYR 53 29.034 -10.546 33.614 -0.1906 1.7000 C

ATOM 836 HD2 TYR 53 28.535 -11.416 33.211 0.1699 1.2000 H

ATOM 837 C TYR 53 31.602 -12.810 30.732 0.5973 1.7000 C

ATOM 838 O TYR 53 30.773 -12.990 29.824 -0.5679 1.5000 O

ATOM 839 N SER 54 32.838 -13.315 30.659 -0.4157 1.5500 N

ATOM 840 H SER 54 33.467 -13.196 31.438 0.2719 1.3000 H

ATOM 841 CA SER 54 33.317 -14.068 29.491 -0.0249 1.7000 C

ATOM 842 HA SER 54 33.208 -13.439 28.608 0.0843 1.2000 H

ATOM 843 CB SER 54 34.797 -14.376 29.669 0.2117 1.7000 C

ATOM 844 HB2 SER 54 35.156 -14.925 28.802 0.0352 1.2000 H

ATOM 845 HB3 SER 54 35.366 -13.449 29.741 0.0352 1.2000 H

ATOM 846 OG SER 54 35.019 -15.167 30.799 -0.6546 1.5000 O

ATOM 847 HG SER 54 35.021 -14.593 31.578 0.4275 1.2000 H

ATOM 848 C SER 54 32.499 -15.351 29.272 0.5973 1.7000 C

ATOM 849 O SER 54 32.415 -15.886 28.158 -0.5679 1.5000 O

ATOM 850 N VAL 55 31.841 -15.785 30.347 -0.4157 1.5500 N

ATOM 851 H VAL 55 31.974 -15.285 31.214 0.2719 1.3000 H

ATOM 852 CA VAL 55 30.975 -16.949 30.409 -0.0875 1.7000 C

ATOM 853 HA VAL 55 31.508 -17.803 29.988 0.0969 1.2000 H

ATOM 854 CB VAL 55 30.659 -17.243 31.884 0.2985 1.7000 C

ATOM 855 HB VAL 55 30.024 -18.128 31.951 -0.0297 1.2000 H

ATOM 856 CG1 VAL 55 31.955 -17.510 32.620 -0.3192 1.7000 C

ATOM 857 HG11 VAL 55 31.732 -17.840 33.635 0.0791 1.2000 H

ATOM 858 HG12 VAL 55 32.512 -18.304 32.121 0.0791 1.2000 H

ATOM 859 HG13 VAL 55 32.579 -16.618 32.686 0.0791 1.2000 H

ATOM 860 CG2 VAL 55 29.944 -16.059 32.509 -0.3192 1.7000 C

ATOM 861 HG21 VAL 55 29.787 -16.258 33.569 0.0791 1.2000 H

ATOM 862 HG22 VAL 55 30.516 -15.136 32.422 0.0791 1.2000 H

ATOM 863 HG23 VAL 55 28.961 -15.934 32.059 0.0791 1.2000 H

ATOM 864 C VAL 55 29.684 -16.758 29.599 0.5973 1.7000 C

ATOM 865 O VAL 55 29.014 -17.731 29.258 -0.5679 1.5000 O

ATOM 866 N ILE 56 29.331 -15.501 29.313 -0.4157 1.5500 N

ATOM 867 H ILE 56 29.908 -14.744 29.647 0.2719 1.3000 H

ATOM 868 CA ILE 56 28.174 -15.168 28.489 -0.0597 1.7000 C

ATOM 869 HA ILE 56 27.488 -16.014 28.434 0.0869 1.2000 H

ATOM 870 CB ILE 56 27.418 -13.932 29.019 0.1303 1.7000 C

ATOM 871 HB ILE 56 28.099 -13.080 29.031 0.0187 1.2000 H

ATOM 872 CG2 ILE 56 26.229 -13.567 28.100 -0.3204 1.7000 C

ATOM 873 HG21 ILE 56 25.616 -12.800 28.573 0.0882 1.2000 H

ATOM 874 HG22 ILE 56 26.591 -13.169 27.152 0.0882 1.2000 H

ATOM 875 HG23 ILE 56 25.612 -14.443 27.899 0.0882 1.2000 H

ATOM 876 CG1 ILE 56 26.975 -14.186 30.460 -0.0430 1.7000 C

ATOM 877 HG12 ILE 56 27.843 -14.237 31.114 0.0236 1.2000 H

ATOM 878 HG13 ILE 56 26.374 -13.344 30.798 0.0236 1.2000 H

ATOM 879 CD1 ILE 56 26.140 -15.421 30.628 -0.0660 1.7000 C

ATOM 880 HD11 ILE 56 25.765 -15.455 31.650 0.0186 1.2000 H

ATOM 881 HD12 ILE 56 25.284 -15.416 29.955 0.0186 1.2000 H

ATOM 882 HD13 ILE 56 26.731 -16.322 30.468 0.0186 1.2000 H

ATOM 883 C ILE 56 28.649 -14.887 27.071 0.5973 1.7000 C

ATOM 884 O ILE 56 28.029 -15.311 26.076 -0.5679 1.5000 O

ATOM 885 N LEU 57 29.761 -14.153 27.000 -0.4157 1.5500 N

ATOM 886 H LEU 57 30.219 -13.856 27.849 0.2719 1.3000 H

ATOM 887 CA LEU 57 30.304 -13.719 25.732 -0.0518 1.7000 C

ATOM 888 HA LEU 57 29.536 -13.094 25.274 0.0922 1.2000 H

ATOM 889 CB LEU 57 31.560 -12.878 25.936 -0.1102 1.7000 C

ATOM 890 HB2 LEU 57 31.297 -11.976 26.493 0.0457 1.2000 H

ATOM 891 HB3 LEU 57 32.264 -13.449 26.541 0.0457 1.2000 H

ATOM 892 CG LEU 57 32.255 -12.501 24.635 0.3531 1.7000 C

ATOM 893 HG LEU 57 32.597 -13.380 24.086 -0.0361 1.2000 H

ATOM 894 CD1 LEU 57 31.301 -11.749 23.814 -0.4121 1.7000 C

ATOM 895 HD11 LEU 57 31.813 -11.389 22.925 0.1000 1.2000 H

ATOM 896 HD12 LEU 57 30.466 -12.345 23.466 0.1000 1.2000 H

ATOM 897 HD13 LEU 57 30.917 -10.890 24.359 0.1000 1.2000 H

ATOM 898 CD2 LEU 57 33.494 -11.663 24.913 -0.4121 1.7000 C

ATOM 899 HD21 LEU 57 33.964 -11.372 23.973 0.1000 1.2000 H

ATOM 900 HD22 LEU 57 33.231 -10.768 25.475 0.1000 1.2000 H

ATOM 901 HD23 LEU 57 34.208 -12.244 25.494 0.1000 1.2000 H

ATOM 902 C LEU 57 30.660 -14.808 24.748 0.5973 1.7000 C

ATOM 903 O LEU 57 30.210 -14.731 23.611 -0.5679 1.5000 O

ATOM 904 N LEU 58 31.424 -15.832 25.135 -0.4157 1.5500 N

ATOM 905 H LEU 58 31.766 -15.930 26.081 0.2719 1.3000 H

ATOM 906 CA LEU 58 31.831 -16.772 24.079 -0.0518 1.7000 C

ATOM 907 HA LEU 58 32.345 -16.178 23.326 0.0922 1.2000 H

ATOM 908 CB LEU 58 32.795 -17.853 24.574 -0.1102 1.7000 C

ATOM 909 HB2 LEU 58 33.650 -17.387 25.053 0.0457 1.2000 H

ATOM 910 HB3 LEU 58 32.282 -18.448 25.332 0.0457 1.2000 H

ATOM 911 CG LEU 58 33.289 -18.796 23.453 0.3531 1.7000 C

ATOM 912 HG LEU 58 32.443 -19.331 23.026 -0.0361 1.2000 H

ATOM 913 CD1 LEU 58 33.996 -17.963 22.363 -0.4121 1.7000 C

ATOM 914 HD11 LEU 58 34.491 -18.629 21.656 0.1000 1.2000 H

ATOM 915 HD12 LEU 58 33.268 -17.376 21.815 0.1000 1.2000 H

ATOM 916 HD13 LEU 58 34.730 -17.288 22.778 0.1000 1.2000 H

ATOM 917 CD2 LEU 58 34.250 -19.820 24.019 -0.4121 1.7000 C

ATOM 918 HD21 LEU 58 35.269 -19.436 24.010 0.1000 1.2000 H

ATOM 919 HD22 LEU 58 33.973 -20.080 25.043 0.1000 1.2000 H

ATOM 920 HD23 LEU 58 34.199 -20.724 23.408 0.1000 1.2000 H

ATOM 921 C LEU 58 30.631 -17.456 23.417 0.5973 1.7000 C

ATOM 922 O LEU 58 30.584 -17.601 22.194 -0.5679 1.5000 O

ATOM 923 N ASP 59 29.654 -17.888 24.209 -0.5163 1.5500 N

ATOM 924 H ASP 59 29.710 -17.793 25.212 0.2936 1.3000 H

ATOM 925 CA ASP 59 28.504 -18.555 23.618 0.0381 1.7000 C

ATOM 926 HA ASP 59 28.856 -19.398 23.021 0.0880 1.2000 H

ATOM 927 CB ASP 59 27.566 -19.091 24.699 -0.0303 1.7000 C

ATOM 928 HB2 ASP 59 27.347 -18.298 25.419 -0.0122 1.2000 H

ATOM 929 HB3 ASP 59 26.626 -19.391 24.233 -0.0122 1.2000 H

ATOM 930 CG ASP 59 28.127 -20.304 25.429 0.7994 1.7000 C

ATOM 931 OD1 ASP 59 29.100 -20.859 24.968 -0.8014 1.5000 O

ATOM 932 OD2 ASP 59 27.558 -20.690 26.420 -0.8014 1.5000 O

ATOM 933 C ASP 59 27.719 -17.639 22.680 0.5366 1.7000 C

ATOM 934 O ASP 59 27.238 -18.075 21.632 -0.5819 1.5000 O

ATOM 935 N THR 60 27.604 -16.355 23.031 -0.4157 1.5500 N

ATOM 936 H THR 60 28.021 -16.020 23.888 0.2719 1.3000 H

ATOM 937 CA THR 60 26.853 -15.438 22.184 -0.0389 1.7000 C

ATOM 938 HA THR 60 25.914 -15.913 21.894 0.1007 1.2000 H

ATOM 939 CB THR 60 26.524 -14.141 22.963 0.3654 1.7000 C

ATOM 940 HB THR 60 27.435 -13.595 23.212 0.0043 1.2000 H

ATOM 941 CG2 THR 60 25.573 -13.256 22.132 -0.2438 1.7000 C

ATOM 942 HG21 THR 60 25.060 -12.549 22.785 0.0642 1.2000 H

ATOM 943 HG22 THR 60 26.135 -12.686 21.406 0.0642 1.2000 H

ATOM 944 HG23 THR 60 24.826 -13.865 21.622 0.0642 1.2000 H

ATOM 945 OG1 THR 60 25.822 -14.483 24.170 -0.6761 1.5000 O

ATOM 946 HG1 THR 60 26.407 -15.005 24.726 0.4102 1.2000 H

ATOM 947 C THR 60 27.678 -15.150 20.904 0.5973 1.7000 C

ATOM 948 O THR 60 27.149 -15.143 19.776 -0.5679 1.5000 O

ATOM 949 N LEU 61 28.990 -14.939 21.105 -0.4157 1.5500 N

ATOM 950 H LEU 61 29.317 -15.025 22.055 0.2719 1.3000 H

ATOM 951 CA LEU 61 30.027 -14.618 20.110 -0.0518 1.7000 C

ATOM 952 HA LEU 61 29.829 -13.625 19.717 0.0922 1.2000 H

ATOM 953 CB LEU 61 31.383 -14.590 20.814 -0.1102 1.7000 C

ATOM 954 HB2 LEU 61 31.240 -13.867 21.615 0.0457 1.2000 H

ATOM 955 HB3 LEU 61 31.584 -15.553 21.273 0.0457 1.2000 H

ATOM 956 CG LEU 61 32.573 -14.157 20.057 0.3531 1.7000 C

ATOM 957 HG LEU 61 32.297 -13.370 19.358 -0.0361 1.2000 H

ATOM 958 CD1 LEU 61 33.528 -13.604 21.040 -0.4121 1.7000 C

ATOM 959 HD11 LEU 61 34.401 -13.290 20.484 0.1000 1.2000 H

ATOM 960 HD12 LEU 61 33.121 -12.724 21.534 0.1000 1.2000 H

ATOM 961 HD13 LEU 61 33.807 -14.353 21.784 0.1000 1.2000 H

ATOM 962 CD2 LEU 61 33.201 -15.324 19.301 -0.4121 1.7000 C

ATOM 963 HD21 LEU 61 33.435 -16.148 19.971 0.1000 1.2000 H

ATOM 964 HD22 LEU 61 32.562 -15.677 18.499 0.1000 1.2000 H

ATOM 965 HD23 LEU 61 34.128 -14.990 18.836 0.1000 1.2000 H

ATOM 966 C LEU 61 30.066 -15.583 18.939 0.5973 1.7000 C

ATOM 967 O LEU 61 30.170 -15.182 17.772 -0.5679 1.5000 O

ATOM 968 N LEU 62 29.997 -16.874 19.264 -0.4157 1.5500 N

ATOM 969 H LEU 62 29.935 -17.129 20.241 0.2719 1.3000 H

ATOM 970 CA LEU 62 30.047 -17.960 18.292 -0.0518 1.7000 C

ATOM 971 HA LEU 62 30.720 -17.699 17.475 0.0922 1.2000 H

ATOM 972 CB LEU 62 30.546 -19.223 18.993 -0.1102 1.7000 C

ATOM 973 HB2 LEU 62 29.841 -19.451 19.796 0.0457 1.2000 H

ATOM 974 HB3 LEU 62 30.505 -20.053 18.286 0.0457 1.2000 H

ATOM 975 CG LEU 62 31.953 -19.180 19.584 0.3531 1.7000 C

ATOM 976 HG LEU 62 32.037 -18.338 20.262 -0.0361 1.2000 H

ATOM 977 CD1 LEU 62 32.168 -20.457 20.358 -0.4121 1.7000 C

ATOM 978 HD11 LEU 62 33.138 -20.432 20.851 0.1000 1.2000 H

ATOM 979 HD12 LEU 62 31.398 -20.566 21.124 0.1000 1.2000 H

ATOM 980 HD13 LEU 62 32.132 -21.319 19.689 0.1000 1.2000 H

ATOM 981 CD2 LEU 62 33.005 -19.026 18.487 -0.4121 1.7000 C

ATOM 982 HD21 LEU 62 34.002 -19.111 18.918 0.1000 1.2000 H

ATOM 983 HD22 LEU 62 32.873 -19.798 17.729 0.1000 1.2000 H

ATOM 984 HD23 LEU 62 32.914 -18.050 18.013 0.1000 1.2000 H

ATOM 985 C LEU 62 28.663 -18.268 17.707 0.5973 1.7000 C

ATOM 986 O LEU 62 28.515 -19.169 16.877 -0.5679 1.5000 O

ATOM 987 N GLY 63 27.650 -17.550 18.187 -0.4157 1.5500 N

ATOM 988 H GLY 63 27.852 -16.847 18.882 0.2719 1.3000 H

ATOM 989 CA GLY 63 26.258 -17.737 17.834 -0.0252 1.7000 C

ATOM 990 HA2 GLY 63 26.125 -18.682 17.307 0.0698 1.2000 H

ATOM 991 HA3 GLY 63 25.682 -17.795 18.758 0.0698 1.2000 H

ATOM 992 C GLY 63 25.696 -16.611 16.970 0.5973 1.7000 C

ATOM 993 O GLY 63 25.677 -16.709 15.738 -0.5679 1.5000 O

ATOM 994 N ARG 64 25.133 -15.592 17.635 -0.3479 1.5500 N

ATOM 995 H ARG 64 25.183 -15.573 18.644 0.2747 1.3000 H

ATOM 996 CA ARG 64 24.447 -14.493 16.947 -0.2637 1.7000 C

ATOM 997 HA ARG 64 24.738 -14.483 15.897 0.1560 1.2000 H

ATOM 998 CB ARG 64 22.935 -14.667 17.045 -0.0007 1.7000 C

ATOM 999 HB2 ARG 64 22.661 -14.733 18.099 0.0327 1.2000 H

ATOM 1000 HB3 ARG 64 22.422 -13.799 16.625 0.0327 1.2000 H

ATOM 1001 CG ARG 64 22.357 -15.889 16.318 0.0390 1.7000 C

ATOM 1002 HG2 ARG 64 22.850 -16.797 16.665 0.0285 1.2000 H

ATOM 1003 HG3 ARG 64 21.297 -15.965 16.565 0.0285 1.2000 H

ATOM 1004 CD ARG 64 22.494 -15.769 14.838 0.0486 1.7000 C

ATOM 1005 HD2 ARG 64 21.991 -14.858 14.511 0.0687 1.2000 H

ATOM 1006 HD3 ARG 64 23.545 -15.686 14.562 0.0687 1.2000 H

ATOM 1007 NE ARG 64 21.895 -16.896 14.140 -0.5295 1.5500 N

ATOM 1008 HE ARG 64 20.933 -16.790 13.857 0.3456 1.3000 H

ATOM 1009 CZ ARG 64 22.520 -18.057 13.832 0.8076 1.7000 C

ATOM 1010 NH1 ARG 64 23.783 -18.270 14.156 -0.8627 1.5500 N

ATOM 1011 HH11 ARG 64 24.254 -19.107 13.853 0.4478 1.3000 H

ATOM 1012 HH12 ARG 64 24.295 -17.576 14.683 0.4478 1.3000 H

ATOM 1013 NH2 ARG 64 21.848 -19.000 13.189 -0.8627 1.5500 N

ATOM 1014 HH21 ARG 64 22.318 -19.844 12.905 0.4478 1.3000 H

ATOM 1015 HH22 ARG 64 20.868 -18.874 12.986 0.4478 1.3000 H

ATOM 1016 C ARG 64 24.807 -13.103 17.480 0.7341 1.7000 C

ATOM 1017 O ARG 64 24.030 -12.158 17.329 -0.5894 1.5000 O

ATOM 1018 N MET 65 25.978 -12.972 18.085 -0.4157 1.5500 N

ATOM 1019 H MET 65 26.582 -13.776 18.182 0.2719 1.3000 H

ATOM 1020 CA MET 65 26.417 -11.703 18.660 -0.0237 1.7000 C

ATOM 1021 HA MET 65 25.694 -11.463 19.442 0.0880 1.2000 H

ATOM 1022 CB MET 65 27.768 -11.837 19.331 0.0342 1.7000 C

ATOM 1023 HB2 MET 65 27.743 -12.667 20.022 0.0241 1.2000 H

ATOM 1024 HB3 MET 65 28.509 -12.066 18.564 0.0241 1.2000 H

ATOM 1025 CG MET 65 28.195 -10.622 20.087 0.0018 1.7000 C

ATOM 1026 HG2 MET 65 28.394 -9.800 19.400 0.0440 1.2000 H

ATOM 1027 HG3 MET 65 27.394 -10.335 20.766 0.0440 1.2000 H

ATOM 1028 SD MET 65 29.615 -10.874 21.007 -0.2737 1.8000 S

ATOM 1029 CE MET 65 30.834 -10.844 19.771 -0.0536 1.7000 C

ATOM 1030 HE1 MET 65 31.811 -10.987 20.232 0.0684 1.2000 H

ATOM 1031 HE2 MET 65 30.663 -11.652 19.065 0.0684 1.2000 H

ATOM 1032 HE3 MET 65 30.812 -9.889 19.245 0.0684 1.2000 H

ATOM 1033 C MET 65 26.493 -10.522 17.716 0.5973 1.7000 C

ATOM 1034 O MET 65 26.881 -10.629 16.552 -0.5679 1.5000 O

ATOM 1035 N LEU 66 26.133 -9.387 18.290 -0.4157 1.5500 N

ATOM 1036 H LEU 66 25.848 -9.723 19.198 0.2719 1.3000 H

ATOM 1037 CA LEU 66 25.880 -7.979 18.486 -0.0518 1.7000 C

ATOM 1038 HA LEU 66 26.332 -7.441 17.657 0.0922 1.2000 H

ATOM 1039 CB LEU 66 24.389 -7.661 18.528 -0.1102 1.7000 C

ATOM 1040 HB2 LEU 66 23.947 -7.951 17.573 0.0457 1.2000 H

ATOM 1041 HB3 LEU 66 23.911 -8.266 19.299 0.0457 1.2000 H

ATOM 1042 CG LEU 66 24.094 -6.190 18.788 0.3531 1.7000 C

ATOM 1043 HG LEU 66 24.555 -5.897 19.724 -0.0361 1.2000 H

ATOM 1044 CD1 LEU 66 24.677 -5.345 17.677 -0.4121 1.7000 C

ATOM 1045 HD11 LEU 66 24.352 -4.310 17.795 0.1000 1.2000 H

ATOM 1046 HD12 LEU 66 25.764 -5.351 17.711 0.1000 1.2000 H

ATOM 1047 HD13 LEU 66 24.337 -5.706 16.705 0.1000 1.2000 H

ATOM 1048 CD2 LEU 66 22.602 -5.968 18.922 -0.4121 1.7000 C

ATOM 1049 HD21 LEU 66 22.399 -4.917 19.133 0.1000 1.2000 H

ATOM 1050 HD22 LEU 66 22.097 -6.249 17.997 0.1000 1.2000 H

ATOM 1051 HD23 LEU 66 22.203 -6.565 19.740 0.1000 1.2000 H

ATOM 1052 C LEU 66 26.501 -7.614 19.833 0.5973 1.7000 C

ATOM 1053 O LEU 66 25.903 -7.917 20.864 -0.5679 1.5000 O

ATOM 1054 N PRO 67 27.707 -7.038 19.893 -0.2548 1.5500 N

ATOM 1055 CD PRO 67 28.475 -6.753 18.670 0.0192 1.7000 C

ATOM 1056 HD2 PRO 67 28.057 -5.888 18.153 0.0391 1.2000 H

ATOM 1057 HD3 PRO 67 28.544 -7.621 18.013 0.0391 1.2000 H

ATOM 1058 CG PRO 67 29.840 -6.417 19.235 0.0189 1.7000 C

ATOM 1059 HG2 PRO 67 30.364 -5.702 18.602 0.0213 1.2000 H

ATOM 1060 HG3 PRO 67 30.421 -7.333 19.329 0.0213 1.2000 H

ATOM 1061 CB PRO 67 29.556 -5.839 20.609 -0.0070 1.7000 C

ATOM 1062 HB2 PRO 67 29.253 -4.795 20.504 0.0253 1.2000 H

ATOM 1063 HB3 PRO 67 30.424 -5.910 21.263 0.0253 1.2000 H

ATOM 1064 CA PRO 67 28.390 -6.685 21.127 -0.0266 1.7000 C

ATOM 1065 HA PRO 67 28.755 -7.590 21.613 0.0641 1.2000 H

ATOM 1066 C PRO 67 27.491 -5.894 22.072 0.5896 1.7000 C

ATOM 1067 O PRO 67 27.576 -6.051 23.289 -0.5748 1.5000 O

ATOM 1068 N GLN 68 26.580 -5.083 21.515 -0.4157 1.5500 N

ATOM 1069 H GLN 68 26.516 -5.010 20.510 0.2719 1.3000 H

ATOM 1070 CA GLN 68 25.666 -4.292 22.333 -0.0031 1.7000 C

ATOM 1071 HA GLN 68 26.239 -3.674 23.025 0.0850 1.2000 H

ATOM 1072 CB GLN 68 24.792 -3.390 21.458 -0.0036 1.7000 C

ATOM 1073 HB2 GLN 68 24.269 -4.008 20.732 0.0171 1.2000 H

ATOM 1074 HB3 GLN 68 24.034 -2.930 22.096 0.0171 1.2000 H

ATOM 1075 CG GLN 68 25.523 -2.281 20.745 -0.0645 1.7000 C

ATOM 1076 HG2 GLN 68 25.947 -1.600 21.484 0.0352 1.2000 H

ATOM 1077 HG3 GLN 68 26.338 -2.696 20.150 0.0352 1.2000 H

ATOM 1078 CD GLN 68 24.593 -1.510 19.815 0.6951 1.7000 C

ATOM 1079 OE1 GLN 68 23.521 -2.001 19.444 -0.6086 1.5000 O

ATOM 1080 NE2 GLN 68 24.997 -0.305 19.432 -0.9407 1.5500 N

ATOM 1081 HE21 GLN 68 24.443 0.197 18.754 0.4251 1.3000 H

ATOM 1082 HE22 GLN 68 25.841 0.095 19.805 0.4251 1.3000 H

ATOM 1083 C GLN 68 24.757 -5.217 23.137 0.5973 1.7000 C

ATOM 1084 O GLN 68 24.399 -4.916 24.277 -0.5679 1.5000 O

ATOM 1085 N LEU 69 24.361 -6.340 22.532 -0.4157 1.5500 N

ATOM 1086 H LEU 69 24.668 -6.550 21.594 0.2719 1.3000 H

ATOM 1087 CA LEU 69 23.499 -7.288 23.202 -0.0518 1.7000 C

ATOM 1088 HA LEU 69 22.599 -6.771 23.539 0.0922 1.2000 H

ATOM 1089 CB LEU 69 23.119 -8.458 22.291 -0.1102 1.7000 C

ATOM 1090 HB2 LEU 69 22.553 -8.070 21.444 0.0457 1.2000 H

ATOM 1091 HB3 LEU 69 24.021 -8.923 21.903 0.0457 1.2000 H

ATOM 1092 CG LEU 69 22.322 -9.553 22.957 0.3531 1.7000 C

ATOM 1093 HG LEU 69 22.877 -9.951 23.803 -0.0361 1.2000 H

ATOM 1094 CD1 LEU 69 21.004 -9.001 23.455 -0.4121 1.7000 C

ATOM 1095 HD11 LEU 69 20.392 -9.817 23.840 0.1000 1.2000 H

ATOM 1096 HD12 LEU 69 21.161 -8.295 24.269 0.1000 1.2000 H

ATOM 1097 HD13 LEU 69 20.464 -8.510 22.645 0.1000 1.2000 H

ATOM 1098 CD2 LEU 69 22.134 -10.695 21.978 -0.4121 1.7000 C

ATOM 1099 HD21 LEU 69 23.102 -11.090 21.669 0.1000 1.2000 H

ATOM 1100 HD22 LEU 69 21.572 -11.498 22.456 0.1000 1.2000 H

ATOM 1101 HD23 LEU 69 21.587 -10.355 21.097 0.1000 1.2000 H

ATOM 1102 C LEU 69 24.229 -7.845 24.373 0.5973 1.7000 C

ATOM 1103 O LEU 69 23.728 -7.832 25.498 -0.5679 1.5000 O

ATOM 1104 N VAL 70 25.449 -8.297 24.117 -0.4157 1.5500 N

ATOM 1105 H VAL 70 25.830 -8.282 23.182 0.2719 1.3000 H

ATOM 1106 CA VAL 70 26.177 -8.931 25.186 -0.0875 1.7000 C

ATOM 1107 HA VAL 70 25.576 -9.757 25.568 0.0969 1.2000 H

ATOM 1108 CB VAL 70 27.521 -9.485 24.746 0.2985 1.7000 C

ATOM 1109 HB VAL 70 28.073 -8.705 24.231 -0.0297 1.2000 H

ATOM 1110 CG1 VAL 70 28.304 -9.955 25.989 -0.3192 1.7000 C

ATOM 1111 HG11 VAL 70 29.178 -10.523 25.682 0.0791 1.2000 H

ATOM 1112 HG12 VAL 70 28.659 -9.110 26.577 0.0791 1.2000 H

ATOM 1113 HG13 VAL 70 27.681 -10.604 26.609 0.0791 1.2000 H

ATOM 1114 CG2 VAL 70 27.303 -10.606 23.824 -0.3192 1.7000 C

ATOM 1115 HG21 VAL 70 28.259 -11.042 23.539 0.0791 1.2000 H

ATOM 1116 HG22 VAL 70 26.704 -11.380 24.307 0.0791 1.2000 H

ATOM 1117 HG23 VAL 70 26.790 -10.268 22.924 0.0791 1.2000 H

ATOM 1118 C VAL 70 26.412 -7.980 26.317 0.5973 1.7000 C

ATOM 1119 O VAL 70 26.161 -8.353 27.452 -0.5679 1.5000 O

ATOM 1120 N CYX 71 26.817 -6.740 26.028 -0.4157 1.5500 N

ATOM 1121 H CYX 71 26.981 -6.465 25.071 0.2719 1.3000 H

ATOM 1122 CA CYX 71 27.093 -5.791 27.099 0.0429 1.7000 C

ATOM 1123 HA CYX 71 27.837 -6.226 27.767 0.0766 1.2000 H

ATOM 1124 CB CYX 71 27.642 -4.486 26.557 -0.0790 1.7000 C

ATOM 1125 HB2 CYX 71 26.925 -4.064 25.852 0.0910 1.2000 H

ATOM 1126 HB3 CYX 71 27.736 -3.803 27.399 0.0910 1.2000 H

ATOM 1127 SG CYX 71 29.266 -4.579 25.752 -0.1081 1.8000 S

ATOM 1128 C CYX 71 25.845 -5.475 27.922 0.5973 1.7000 C

ATOM 1129 O CYX 71 25.942 -5.109 29.105 -0.5679 1.5000 O

ATOM 1130 N ARG 72 24.656 -5.606 27.322 -0.3479 1.5500 N

ATOM 1131 H ARG 72 24.583 -5.910 26.360 0.2747 1.3000 H

ATOM 1132 CA ARG 72 23.445 -5.388 28.095 -0.2637 1.7000 C

ATOM 1133 HA ARG 72 23.592 -4.566 28.797 0.1560 1.2000 H

ATOM 1134 CB ARG 72 22.287 -5.012 27.190 -0.0007 1.7000 C

ATOM 1135 HB2 ARG 72 22.207 -5.748 26.388 0.0327 1.2000 H

ATOM 1136 HB3 ARG 72 21.369 -5.064 27.778 0.0327 1.2000 H

ATOM 1137 CG ARG 72 22.392 -3.619 26.584 0.0390 1.7000 C

ATOM 1138 HG2 ARG 72 22.431 -2.880 27.387 0.0285 1.2000 H

ATOM 1139 HG3 ARG 72 23.317 -3.530 26.020 0.0285 1.2000 H

ATOM 1140 CD ARG 72 21.251 -3.314 25.683 0.0486 1.7000 C

ATOM 1141 HD2 ARG 72 21.452 -2.385 25.146 0.0687 1.2000 H

ATOM 1142 HD3 ARG 72 21.159 -4.111 24.943 0.0687 1.2000 H

ATOM 1143 NE ARG 72 19.997 -3.206 26.423 -0.5295 1.5500 N

ATOM 1144 HE ARG 72 19.418 -4.029 26.489 0.3456 1.3000 H

ATOM 1145 CZ ARG 72 19.594 -2.076 27.041 0.8076 1.7000 C

ATOM 1146 NH1 ARG 72 20.313 -0.999 26.991 -0.8627 1.5500 N

ATOM 1147 HH11 ARG 72 21.157 -0.971 26.448 0.4478 1.3000 H

ATOM 1148 HH12 ARG 72 20.022 -0.184 27.519 0.4478 1.3000 H

ATOM 1149 NH2 ARG 72 18.478 -2.007 27.722 -0.8627 1.5500 N

ATOM 1150 HH21 ARG 72 17.847 -2.790 27.756 0.4478 1.3000 H

ATOM 1151 HH22 ARG 72 18.254 -1.146 28.205 0.4478 1.3000 H

ATOM 1152 C ARG 72 23.094 -6.639 28.913 0.7341 1.7000 C

ATOM 1153 O ARG 72 22.742 -6.535 30.089 -0.5894 1.5000 O

ATOM 1154 N LEU 73 23.310 -7.831 28.343 -0.4157 1.5500 N

ATOM 1155 H LEU 73 23.659 -7.874 27.397 0.2719 1.3000 H

ATOM 1156 CA LEU 73 22.971 -9.093 29.014 -0.0518 1.7000 C

ATOM 1157 HA LEU 73 21.910 -9.093 29.262 0.0922 1.2000 H

ATOM 1158 CB LEU 73 23.301 -10.306 28.115 -0.1102 1.7000 C

ATOM 1159 HB2 LEU 73 24.344 -10.224 27.808 0.0457 1.2000 H

ATOM 1160 HB3 LEU 73 23.227 -11.210 28.724 0.0457 1.2000 H

ATOM 1161 CG LEU 73 22.445 -10.520 26.854 0.3531 1.7000 C

ATOM 1162 HG LEU 73 22.425 -9.627 26.247 -0.0361 1.2000 H

ATOM 1163 CD1 LEU 73 23.055 -11.670 26.029 -0.4121 1.7000 C

ATOM 1164 HD11 LEU 73 22.435 -11.868 25.154 0.1000 1.2000 H

ATOM 1165 HD12 LEU 73 24.058 -11.405 25.696 0.1000 1.2000 H

ATOM 1166 HD13 LEU 73 23.107 -12.576 26.634 0.1000 1.2000 H

ATOM 1167 CD2 LEU 73 21.028 -10.823 27.251 -0.4121 1.7000 C

ATOM 1168 HD21 LEU 73 20.442 -11.050 26.360 0.1000 1.2000 H

ATOM 1169 HD22 LEU 73 20.995 -11.683 27.921 0.1000 1.2000 H

ATOM 1170 HD23 LEU 73 20.576 -9.962 27.742 0.1000 1.2000 H

ATOM 1171 C LEU 73 23.753 -9.273 30.304 0.5973 1.7000 C

ATOM 1172 O LEU 73 23.256 -9.834 31.283 -0.5679 1.5000 O

ATOM 1173 N VAL 74 24.979 -8.782 30.295 -0.4157 1.5500 N

ATOM 1174 H VAL 74 25.320 -8.348 29.450 0.2719 1.3000 H

ATOM 1175 CA VAL 74 25.900 -8.915 31.409 -0.0875 1.7000 C

ATOM 1176 HA VAL 74 25.610 -9.751 32.050 0.0969 1.2000 H

ATOM 1177 CB VAL 74 27.266 -9.185 30.832 0.2985 1.7000 C

ATOM 1178 HB VAL 74 27.974 -9.351 31.632 -0.0297 1.2000 H

ATOM 1179 CG1 VAL 74 27.235 -10.407 29.975 -0.3192 1.7000 C

ATOM 1180 HG11 VAL 74 28.232 -10.627 29.596 0.0791 1.2000 H

ATOM 1181 HG12 VAL 74 26.900 -11.246 30.579 0.0791 1.2000 H

ATOM 1182 HG13 VAL 74 26.579 -10.304 29.112 0.0791 1.2000 H

ATOM 1183 CG2 VAL 74 27.659 -8.034 30.123 -0.3192 1.7000 C

ATOM 1184 HG21 VAL 74 28.534 -8.261 29.518 0.0791 1.2000 H

ATOM 1185 HG22 VAL 74 26.910 -7.610 29.478 0.0791 1.2000 H

ATOM 1186 HG23 VAL 74 27.914 -7.231 30.790 0.0791 1.2000 H

ATOM 1187 C VAL 74 26.009 -7.654 32.267 0.5973 1.7000 C

ATOM 1188 O VAL 74 26.833 -7.594 33.176 -0.5679 1.5000 O

ATOM 1189 N LEU 75 25.226 -6.625 31.943 -0.4157 1.5500 N

ATOM 1190 H LEU 75 24.556 -6.728 31.193 0.2719 1.3000 H

ATOM 1191 CA LEU 75 25.266 -5.344 32.645 -0.0518 1.7000 C

ATOM 1192 HA LEU 75 24.598 -4.663 32.115 0.0922 1.2000 H

ATOM 1193 CB LEU 75 24.773 -5.521 34.085 -0.1102 1.7000 C

ATOM 1194 HB2 LEU 75 25.463 -6.149 34.647 0.0457 1.2000 H

ATOM 1195 HB3 LEU 75 24.762 -4.544 34.571 0.0457 1.2000 H

ATOM 1196 CG LEU 75 23.371 -6.103 34.196 0.3531 1.7000 C

ATOM 1197 HG LEU 75 23.340 -7.098 33.749 -0.0361 1.2000 H

ATOM 1198 CD1 LEU 75 22.983 -6.241 35.648 -0.4121 1.7000 C

ATOM 1199 HD11 LEU 75 21.979 -6.661 35.718 0.1000 1.2000 H

ATOM 1200 HD12 LEU 75 23.673 -6.919 36.152 0.1000 1.2000 H

ATOM 1201 HD13 LEU 75 22.996 -5.267 36.138 0.1000 1.2000 H

ATOM 1202 CD2 LEU 75 22.434 -5.210 33.452 -0.4121 1.7000 C

ATOM 1203 HD21 LEU 75 21.408 -5.521 33.646 0.1000 1.2000 H

ATOM 1204 HD22 LEU 75 22.549 -4.173 33.772 0.1000 1.2000 H

ATOM 1205 HD23 LEU 75 22.595 -5.289 32.381 0.1000 1.2000 H

ATOM 1206 C LEU 75 26.639 -4.648 32.652 0.5973 1.7000 C

ATOM 1207 O LEU 75 27.051 -4.095 33.672 -0.5679 1.5000 O

ATOM 1208 N ARG 76 27.365 -4.692 31.526 -0.3479 1.5500 N

ATOM 1209 H ARG 76 27.004 -5.175 30.716 0.2747 1.3000 H

ATOM 1210 CA ARG 76 28.639 -3.979 31.420 -0.2637 1.7000 C

ATOM 1211 HA ARG 76 29.034 -3.802 32.421 0.1560 1.2000 H

ATOM 1212 CB ARG 76 29.715 -4.755 30.683 -0.0007 1.7000 C

ATOM 1213 HB2 ARG 76 29.306 -5.081 29.727 0.0327 1.2000 H

ATOM 1214 HB3 ARG 76 30.545 -4.070 30.496 0.0327 1.2000 H

ATOM 1215 CG ARG 76 30.296 -5.981 31.431 0.0390 1.7000 C

ATOM 1216 HG2 ARG 76 30.703 -5.611 32.369 0.0285 1.2000 H

ATOM 1217 HG3 ARG 76 29.547 -6.696 31.738 0.0285 1.2000 H

ATOM 1218 CD ARG 76 31.439 -6.698 30.678 0.0486 1.7000 C

ATOM 1219 HD2 ARG 76 32.172 -5.951 30.373 0.0687 1.2000 H

ATOM 1220 HD3 ARG 76 31.928 -7.381 31.363 0.0687 1.2000 H

ATOM 1221 NE ARG 76 31.041 -7.433 29.519 -0.5295 1.5500 N

ATOM 1222 HE ARG 76 30.082 -7.334 29.224 0.3456 1.3000 H

ATOM 1223 CZ ARG 76 31.857 -8.232 28.791 0.8076 1.7000 C

ATOM 1224 NH1 ARG 76 33.107 -8.399 29.128 -0.8627 1.5500 N

ATOM 1225 HH11 ARG 76 33.705 -8.994 28.575 0.4478 1.3000 H

ATOM 1226 HH12 ARG 76 33.463 -7.940 29.953 0.4478 1.3000 H

ATOM 1227 NH2 ARG 76 31.406 -8.858 27.738 -0.8627 1.5500 N

ATOM 1228 HH21 ARG 76 30.443 -8.742 27.461 0.4478 1.3000 H

ATOM 1229 HH22 ARG 76 32.019 -9.456 27.206 0.4478 1.3000 H

ATOM 1230 C ARG 76 28.428 -2.595 30.811 0.7341 1.7000 C

ATOM 1231 O ARG 76 29.233 -1.690 31.018 -0.5894 1.5000 O

ATOM 1232 N CYX 77 27.331 -2.432 30.070 -0.4157 1.5500 N

ATOM 1233 H CYX 77 26.692 -3.206 29.953 0.2719 1.3000 H

ATOM 1234 CA CYX 77 26.939 -1.126 29.529 0.0429 1.7000 C

ATOM 1235 HA CYX 77 27.685 -0.388 29.818 0.0766 1.2000 H

ATOM 1236 CB CYX 77 26.873 -1.083 28.010 -0.0790 1.7000 C

ATOM 1237 HB2 CYX 77 26.260 -1.918 27.669 0.0910 1.2000 H

ATOM 1238 HB3 CYX 77 26.392 -0.159 27.687 0.0910 1.2000 H

ATOM 1239 SG CYX 77 28.475 -1.178 27.225 -0.1081 1.8000 S

ATOM 1240 C CYX 77 25.634 -0.636 30.121 0.5973 1.7000 C

ATOM 1241 O CYX 77 24.827 -1.421 30.612 -0.5679 1.5000 O

ATOM 1242 N SER 78 25.432 0.677 30.075 -0.4157 1.5500 N

ATOM 1243 H SER 78 26.132 1.282 29.675 0.2719 1.3000 H

ATOM 1244 CA SER 78 24.222 1.282 30.613 -0.0249 1.7000 C

ATOM 1245 HA SER 78 24.199 1.108 31.690 0.0843 1.2000 H

ATOM 1246 CB SER 78 24.231 2.776 30.379 0.2117 1.7000 C

ATOM 1247 HB2 SER 78 25.076 3.219 30.907 0.0352 1.2000 H

ATOM 1248 HB3 SER 78 24.332 2.980 29.312 0.0352 1.2000 H

ATOM 1249 OG SER 78 23.044 3.359 30.844 -0.6546 1.5000 O

ATOM 1250 HG SER 78 22.301 3.056 30.307 0.4275 1.2000 H

ATOM 1251 C SER 78 22.985 0.671 29.980 0.5973 1.7000 C

ATOM 1252 O SER 78 22.967 0.399 28.775 -0.5679 1.5000 O

ATOM 1253 N MET 79 21.978 0.445 30.815 -0.3821 1.5500 N

ATOM 1254 H MET 79 22.066 0.721 31.781 0.2681 1.3000 H

ATOM 1255 CA MET 79 20.727 -0.153 30.399 -0.2597 1.7000 C

ATOM 1256 HA MET 79 20.908 -0.791 29.537 0.1277 1.2000 H

ATOM 1257 CB MET 79 20.202 -1.021 31.529 -0.0236 1.7000 C

ATOM 1258 HB2 MET 79 19.999 -0.393 32.398 0.0480 1.2000 H

ATOM 1259 HB3 MET 79 19.262 -1.480 31.225 0.0480 1.2000 H

ATOM 1260 CG MET 79 21.195 -2.091 31.927 0.0492 1.7000 C

ATOM 1261 HG2 MET 79 22.115 -1.633 32.291 0.0317 1.2000 H

ATOM 1262 HG3 MET 79 20.771 -2.681 32.740 0.0317 1.2000 H

ATOM 1263 SD MET 79 21.592 -3.187 30.561 -0.2692 1.8000 S

ATOM 1264 CE MET 79 20.048 -4.060 30.405 -0.0376 1.7000 C

ATOM 1265 HE1 MET 79 19.268 -3.386 30.057 0.0625 1.2000 H

ATOM 1266 HE2 MET 79 20.162 -4.871 29.689 0.0625 1.2000 H

ATOM 1267 HE3 MET 79 19.767 -4.482 31.370 0.0625 1.2000 H

ATOM 1268 C MET 79 19.710 0.901 29.972 0.8013 1.7000 C

ATOM 1269 O MET 79 19.022 0.739 28.957 -0.8105 1.5000 O

ATOM 1270 OXT MET 79 19.753 2.005 30.511 -0.8105 1.5000 O

TER 1271 MET 79

END

SP-B Open PDB

ATOM 1 N PHE 1 34.501 -20.742 -14.102 0.1737 1.5500 N

ATOM 2 H1 PHE 1 35.098 -20.743 -13.287 0.1921 1.3000 H

ATOM 3 H2 PHE 1 33.628 -20.287 -13.872 0.1921 1.3000 H

ATOM 4 H3 PHE 1 34.323 -21.693 -14.393 0.1921 1.3000 H

ATOM 5 CA PHE 1 35.151 -20.011 -15.186 0.0733 1.7000 C

ATOM 6 HA PHE 1 36.131 -20.454 -15.351 0.1041 1.2000 H

ATOM 7 CB PHE 1 34.345 -20.133 -16.485 0.0330 1.7000 C

ATOM 8 HB2 PHE 1 33.304 -19.872 -16.288 0.0104 1.2000 H

ATOM 9 HB3 PHE 1 34.724 -19.406 -17.205 0.0104 1.2000 H

ATOM 10 CG PHE 1 34.413 -21.495 -17.130 0.0031 1.7000 C

ATOM 11 CD1 PHE 1 33.401 -22.430 -16.951 -0.1392 1.7000 C

ATOM 12 HD1 PHE 1 32.539 -22.186 -16.347 0.1374 1.2000 H

ATOM 13 CE1 PHE 1 33.474 -23.677 -17.555 -0.1602 1.7000 C

ATOM 14 HE1 PHE 1 32.681 -24.396 -17.408 0.1433 1.2000 H

ATOM 15 CZ PHE 1 34.558 -24.001 -18.346 -0.1208 1.7000 C

ATOM 16 HZ PHE 1 34.612 -24.970 -18.822 0.1329 1.2000 H

ATOM 17 CE2 PHE 1 35.570 -23.080 -18.534 -0.1603 1.7000 C

ATOM 18 HE2 PHE 1 36.419 -23.331 -19.153 0.1433 1.2000 H

ATOM 19 CD2 PHE 1 35.496 -21.838 -17.931 -0.1391 1.7000 C

ATOM 20 HD2 PHE 1 36.291 -21.125 -18.095 0.1374 1.2000 H

ATOM 21 C PHE 1 35.325 -18.527 -14.831 0.6123 1.7000 C

ATOM 22 O PHE 1 34.554 -18.004 -14.027 -0.5713 1.5000 O

ATOM 23 N PRO 2 36.352 -17.833 -15.366 -0.2548 1.5500 N

ATOM 24 CD PRO 2 37.418 -18.514 -16.157 0.0192 1.7000 C

ATOM 25 HD2 PRO 2 37.067 -18.705 -17.172 0.0391 1.2000 H

ATOM 26 HD3 PRO 2 37.775 -19.427 -15.679 0.0391 1.2000 H

ATOM 27 CG PRO 2 38.516 -17.470 -16.169 0.0189 1.7000 C

ATOM 28 HG2 PRO 2 39.125 -17.541 -17.071 0.0213 1.2000 H

ATOM 29 HG3 PRO 2 39.142 -17.598 -15.284 0.0213 1.2000 H

ATOM 30 CB PRO 2 37.803 -16.145 -16.084 -0.0070 1.7000 C

ATOM 31 HB2 PRO 2 37.485 -15.852 -17.087 0.0253 1.2000 H

ATOM 32 HB3 PRO 2 38.445 -15.373 -15.658 0.0253 1.2000 H

ATOM 33 CA PRO 2 36.583 -16.407 -15.195 -0.0266 1.7000 C

ATOM 34 HA PRO 2 36.814 -16.188 -14.151 0.0641 1.2000 H

ATOM 35 C PRO 2 35.390 -15.605 -15.681 0.5896 1.7000 C

ATOM 36 O PRO 2 34.727 -15.987 -16.650 -0.5748 1.5000 O

ATOM 37 N ILE 3 35.135 -14.487 -15.026 -0.4157 1.5500 N

ATOM 38 H ILE 3 35.727 -14.207 -14.257 0.2719 1.3000 H

ATOM 39 CA ILE 3 34.043 -13.607 -15.396 -0.0597 1.7000 C

ATOM 40 HA ILE 3 33.273 -14.197 -15.880 0.0869 1.2000 H

ATOM 41 CB ILE 3 33.434 -12.985 -14.142 0.1303 1.7000 C

ATOM 42 HB ILE 3 34.193 -12.397 -13.623 0.0187 1.2000 H

ATOM 43 CG2 ILE 3 32.318 -12.076 -14.524 -0.3204 1.7000 C

ATOM 44 HG21 ILE 3 31.753 -11.818 -13.639 0.0882 1.2000 H

ATOM 45 HG22 ILE 3 32.711 -11.151 -14.940 0.0882 1.2000 H

ATOM 46 HG23 ILE 3 31.647 -12.546 -15.241 0.0882 1.2000 H

ATOM 47 CG1 ILE 3 32.960 -14.094 -13.196 -0.0430 1.7000 C

ATOM 48 HG12 ILE 3 33.801 -14.699 -12.857 0.0236 1.2000 H

ATOM 49 HG13 ILE 3 32.534 -13.637 -12.305 0.0236 1.2000 H

ATOM 50 CD1 ILE 3 31.921 -14.990 -13.794 -0.0660 1.7000 C

ATOM 51 HD11 ILE 3 31.587 -15.689 -13.027 0.0186 1.2000 H

ATOM 52 HD12 ILE 3 31.052 -14.425 -14.128 0.0186 1.2000 H

ATOM 53 HD13 ILE 3 32.325 -15.582 -14.614 0.0186 1.2000 H

ATOM 54 C ILE 3 34.524 -12.495 -16.335 0.5973 1.7000 C

ATOM 55 O ILE 3 35.524 -11.849 -16.033 -0.5679 1.5000 O

ATOM 56 N PRO 4 33.893 -12.257 -17.496 -0.2548 1.5500 N

ATOM 57 CD PRO 4 32.784 -13.106 -17.982 0.0192 1.7000 C

ATOM 58 HD2 PRO 4 31.864 -12.878 -17.444 0.0391 1.2000 H

ATOM 59 HD3 PRO 4 33.023 -14.169 -17.945 0.0391 1.2000 H

ATOM 60 CG PRO 4 32.672 -12.653 -19.431 0.0189 1.7000 C

ATOM 61 HG2 PRO 4 31.649 -12.741 -19.798 0.0213 1.2000 H

ATOM 62 HG3 PRO 4 33.346 -13.249 -20.049 0.0213 1.2000 H

ATOM 63 CB PRO 4 33.141 -11.209 -19.425 -0.0070 1.7000 C

ATOM 64 HB2 PRO 4 32.317 -10.569 -19.104 0.0253 1.2000 H

ATOM 65 HB3 PRO 4 33.493 -10.899 -20.409 0.0253 1.2000 H

ATOM 66 CA PRO 4 34.275 -11.191 -18.396 -0.0266 1.7000 C

ATOM 67 HA PRO 4 35.237 -11.412 -18.862 0.0641 1.2000 H

ATOM 68 C PRO 4 34.300 -9.892 -17.619 0.5896 1.7000 C

ATOM 69 O PRO 4 33.362 -9.598 -16.871 -0.5748 1.5000 O

ATOM 70 N LEU 5 35.325 -9.079 -17.829 -0.4157 1.5500 N

ATOM 71 H LEU 5 36.084 -9.340 -18.441 0.2719 1.3000 H

ATOM 72 CA LEU 5 35.398 -7.834 -17.087 -0.0518 1.7000 C

ATOM 73 HA LEU 5 35.457 -8.136 -16.041 0.0922 1.2000 H

ATOM 74 CB LEU 5 36.690 -7.074 -17.399 -0.1102 1.7000 C

ATOM 75 HB2 LEU 5 37.532 -7.718 -17.136 0.0457 1.2000 H

ATOM 76 HB3 LEU 5 36.762 -6.886 -18.468 0.0457 1.2000 H

ATOM 77 CG LEU 5 36.822 -5.778 -16.683 0.3531 1.7000 C

ATOM 78 HG LEU 5 35.998 -5.118 -16.947 -0.0361 1.2000 H

ATOM 79 CD1 LEU 5 36.811 -6.033 -15.186 -0.4121 1.7000 C

ATOM 80 HD11 LEU 5 37.069 -5.114 -14.661 0.1000 1.2000 H

ATOM 81 HD12 LEU 5 35.828 -6.338 -14.838 0.1000 1.2000 H

ATOM 82 HD13 LEU 5 37.543 -6.798 -14.921 0.1000 1.2000 H

ATOM 83 CD2 LEU 5 38.111 -5.114 -17.103 -0.4121 1.7000 C

ATOM 84 HD21 LEU 5 38.247 -4.185 -16.550 0.1000 1.2000 H

ATOM 85 HD22 LEU 5 38.960 -5.768 -16.897 0.1000 1.2000 H

ATOM 86 HD23 LEU 5 38.083 -4.885 -18.169 0.1000 1.2000 H

ATOM 87 C LEU 5 34.133 -6.961 -17.213 0.5973 1.7000 C

ATOM 88 O LEU 5 33.680 -6.444 -16.195 -0.5679 1.5000 O

ATOM 89 N PRO 6 33.534 -6.745 -18.410 -0.2548 1.5500 N

ATOM 90 CD PRO 6 34.123 -7.188 -19.696 0.0192 1.7000 C

ATOM 91 HD2 PRO 6 33.868 -8.230 -19.888 0.0391 1.2000 H

ATOM 92 HD3 PRO 6 35.192 -7.020 -19.770 0.0391 1.2000 H

ATOM 93 CG PRO 6 33.410 -6.305 -20.681 0.0189 1.7000 C

ATOM 94 HG2 PRO 6 33.348 -6.772 -21.665 0.0213 1.2000 H

ATOM 95 HG3 PRO 6 33.929 -5.347 -20.750 0.0213 1.2000 H

ATOM 96 CB PRO 6 32.048 -6.093 -20.082 -0.0070 1.7000 C

ATOM 97 HB2 PRO 6 31.422 -6.958 -20.308 0.0253 1.2000 H

ATOM 98 HB3 PRO 6 31.580 -5.184 -20.463 0.0253 1.2000 H

ATOM 99 CA PRO 6 32.297 -6.002 -18.573 -0.0266 1.7000 C

ATOM 100 HA PRO 6 32.462 -4.967 -18.284 0.0641 1.2000 H

ATOM 101 C PRO 6 31.122 -6.587 -17.784 0.5896 1.7000 C

ATOM 102 O PRO 6 30.156 -5.877 -17.487 -0.5748 1.5000 O

ATOM 103 N TYR 7 31.165 -7.884 -17.456 -0.4157 1.5500 N

ATOM 104 H TYR 7 31.963 -8.456 -17.694 0.2719 1.3000 H

ATOM 105 CA TYR 7 30.072 -8.467 -16.700 -0.0014 1.7000 C

ATOM 106 HA TYR 7 29.128 -8.082 -17.089 0.0876 1.2000 H

ATOM 107 CB TYR 7 30.026 -9.982 -16.798 -0.0152 1.7000 C

ATOM 108 HB2 TYR 7 30.049 -10.272 -17.850 0.0295 1.2000 H

ATOM 109 HB3 TYR 7 30.900 -10.412 -16.315 0.0295 1.2000 H

ATOM 110 CG TYR 7 28.769 -10.533 -16.174 -0.0011 1.7000 C

ATOM 111 CD1 TYR 7 27.570 -10.416 -16.871 -0.1906 1.7000 C

ATOM 112 HD1 TYR 7 27.552 -9.947 -17.845 0.1699 1.2000 H

ATOM 113 CE1 TYR 7 26.400 -10.897 -16.323 -0.2341 1.7000 C

ATOM 114 HE1 TYR 7 25.470 -10.809 -16.866 0.1656 1.2000 H

ATOM 115 CZ TYR 7 26.430 -11.497 -15.076 0.3226 1.7000 C

ATOM 116 OH TYR 7 25.265 -11.978 -14.520 -0.5579 1.5000 O

ATOM 117 HH TYR 7 24.537 -11.992 -15.144 0.3992 1.2000 H

ATOM 118 CE2 TYR 7 27.616 -11.609 -14.393 -0.2341 1.7000 C

ATOM 119 HE2 TYR 7 27.633 -12.090 -13.431 0.1656 1.2000 H

ATOM 120 CD2 TYR 7 28.782 -11.130 -14.932 -0.1906 1.7000 C

ATOM 121 HD2 TYR 7 29.702 -11.224 -14.383 0.1699 1.2000 H

ATOM 122 C TYR 7 30.239 -7.985 -15.273 0.5973 1.7000 C

ATOM 123 O TYR 7 29.272 -7.583 -14.620 -0.5679 1.5000 O

ATOM 124 N CYX 8 31.491 -8.023 -14.798 -0.4157 1.5500 N

ATOM 125 H CYX 8 32.245 -8.368 -15.376 0.2719 1.3000 H

ATOM 126 CA CYX 8 31.797 -7.511 -13.470 0.0429 1.7000 C

ATOM 127 HA CYX 8 31.226 -8.076 -12.735 0.0766 1.2000 H

ATOM 128 CB CYX 8 33.283 -7.643 -13.137 -0.0790 1.7000 C

ATOM 129 HB2 CYX 8 33.568 -8.694 -13.201 0.0910 1.2000 H

ATOM 130 HB3 CYX 8 33.872 -7.092 -13.864 0.0910 1.2000 H

ATOM 131 SG CYX 8 33.703 -7.016 -11.485 -0.1081 1.8000 S

ATOM 132 C CYX 8 31.399 -6.049 -13.381 0.5973 1.7000 C

ATOM 133 O CYX 8 30.749 -5.615 -12.428 -0.5679 1.5000 O

ATOM 134 N TRP 9 31.770 -5.264 -14.394 -0.4157 1.5500 N

ATOM 135 H TRP 9 32.298 -5.634 -15.172 0.2719 1.3000 H

ATOM 136 CA TRP 9 31.407 -3.859 -14.376 -0.0275 1.7000 C

ATOM 137 HA TRP 9 31.870 -3.373 -13.518 0.1123 1.2000 H

ATOM 138 CB TRP 9 31.850 -3.167 -15.659 -0.0050 1.7000 C

ATOM 139 HB2 TRP 9 31.518 -3.746 -16.520 0.0339 1.2000 H

ATOM 140 HB3 TRP 9 31.365 -2.191 -15.712 0.0339 1.2000 H

ATOM 141 CG TRP 9 33.317 -2.930 -15.764 -0.1415 1.7000 C

ATOM 142 CD1 TRP 9 34.218 -2.903 -14.746 -0.1638 1.7000 C

ATOM 143 HD1 TRP 9 33.991 -3.056 -13.703 0.2062 1.2000 H

ATOM 144 NE1 TRP 9 35.471 -2.630 -15.236 -0.3418 1.5500 N

ATOM 145 HE1 TRP 9 36.305 -2.562 -14.672 0.3412 1.3000 H

ATOM 146 CE2 TRP 9 35.391 -2.481 -16.597 0.1380 1.7000 C

ATOM 147 CZ2 TRP 9 36.378 -2.202 -17.524 -0.2601 1.7000 C

ATOM 148 HZ2 TRP 9 37.402 -2.044 -17.222 0.1572 1.2000 H

ATOM 149 CH2 TRP 9 36.006 -2.124 -18.860 -0.1134 1.7000 C

ATOM 150 HH2 TRP 9 36.759 -1.919 -19.606 0.1417 1.2000 H

ATOM 151 CZ3 TRP 9 34.711 -2.305 -19.251 -0.1972 1.7000 C

ATOM 152 HZ3 TRP 9 34.454 -2.224 -20.297 0.1447 1.2000 H

ATOM 153 CE3 TRP 9 33.721 -2.570 -18.325 -0.2387 1.7000 C

ATOM 154 HE3 TRP 9 32.697 -2.703 -18.641 0.1700 1.2000 H

ATOM 155 CD2 TRP 9 34.067 -2.669 -16.973 0.1243 1.7000 C

ATOM 156 C TRP 9 29.896 -3.702 -14.236 0.5973 1.7000 C

ATOM 157 O TRP 9 29.430 -2.899 -13.420 -0.5679 1.5000 O

ATOM 158 N LEU 10 29.124 -4.497 -14.985 -0.4157 1.5500 N

ATOM 159 H LEU 10 29.535 -5.145 -15.643 0.2719 1.3000 H

ATOM 160 CA LEU 10 27.678 -4.434 -14.869 -0.0518 1.7000 C

ATOM 161 HA LEU 10 27.380 -3.412 -15.106 0.0922 1.2000 H

ATOM 162 CB LEU 10 26.978 -5.376 -15.844 -0.1102 1.7000 C

ATOM 163 HB2 LEU 10 27.203 -5.053 -16.862 0.0457 1.2000 H

ATOM 164 HB3 LEU 10 27.380 -6.380 -15.724 0.0457 1.2000 H

ATOM 165 CG LEU 10 25.446 -5.447 -15.654 0.3531 1.7000 C

ATOM 166 HG LEU 10 25.194 -5.783 -14.649 -0.0361 1.2000 H

ATOM 167 CD1 LEU 10 24.834 -4.066 -15.881 -0.4121 1.7000 C

ATOM 168 HD11 LEU 10 23.748 -4.138 -15.820 0.1000 1.2000 H

ATOM 169 HD12 LEU 10 25.163 -3.354 -15.127 0.1000 1.2000 H

ATOM 170 HD13 LEU 10 25.106 -3.694 -16.870 0.1000 1.2000 H

ATOM 171 CD2 LEU 10 24.870 -6.483 -16.604 -0.4121 1.7000 C

ATOM 172 HD21 LEU 10 25.299 -7.463 -16.388 0.1000 1.2000 H

ATOM 173 HD22 LEU 10 23.788 -6.539 -16.477 0.1000 1.2000 H

ATOM 174 HD23 LEU 10 25.095 -6.213 -17.637 0.1000 1.2000 H

ATOM 175 C LEU 10 27.172 -4.765 -13.485 0.5973 1.7000 C

ATOM 176 O LEU 10 26.362 -4.015 -12.940 -0.5679 1.5000 O

ATOM 177 N CYX 11 27.611 -5.881 -12.894 -0.4157 1.5500 N

ATOM 178 H CYX 11 28.271 -6.503 -13.341 0.2719 1.3000 H

ATOM 179 CA CYX 11 27.032 -6.205 -11.596 0.0429 1.7000 C

ATOM 180 HA CYX 11 25.947 -6.236 -11.690 0.0766 1.2000 H

ATOM 181 CB CYX 11 27.504 -7.575 -11.114 -0.0790 1.7000 C

ATOM 182 HB2 CYX 11 26.907 -7.870 -10.254 0.0910 1.2000 H

ATOM 183 HB3 CYX 11 27.334 -8.300 -11.911 0.0910 1.2000 H

ATOM 184 SG CYX 11 29.237 -7.633 -10.646 -0.1081 1.8000 S

ATOM 185 C CYX 11 27.410 -5.160 -10.557 0.5973 1.7000 C

ATOM 186 O CYX 11 26.617 -4.841 -9.668 -0.5679 1.5000 O

ATOM 187 N ARG 12 28.567 -4.526 -10.732 -0.3479 1.5500 N

ATOM 188 H ARG 12 29.185 -4.781 -11.491 0.2747 1.3000 H

ATOM 189 CA ARG 12 28.971 -3.506 -9.796 -0.2637 1.7000 C

ATOM 190 HA ARG 12 28.802 -3.869 -8.781 0.1560 1.2000 H

ATOM 191 CB ARG 12 30.446 -3.209 -9.947 -0.0007 1.7000 C

ATOM 192 HB2 ARG 12 30.667 -2.994 -10.994 0.0327 1.2000 H

ATOM 193 HB3 ARG 12 30.678 -2.318 -9.362 0.0327 1.2000 H

ATOM 194 CG ARG 12 31.329 -4.354 -9.464 0.0390 1.7000 C

ATOM 195 HG2 ARG 12 31.172 -4.505 -8.397 0.0285 1.2000 H

ATOM 196 HG3 ARG 12 31.044 -5.277 -9.963 0.0285 1.2000 H

ATOM 197 CD ARG 12 32.752 -4.130 -9.716 0.0486 1.7000 C

ATOM 198 HD2 ARG 12 33.293 -5.032 -9.429 0.0687 1.2000 H

ATOM 199 HD3 ARG 12 32.894 -3.966 -10.786 0.0687 1.2000 H

ATOM 200 NE ARG 12 33.299 -3.014 -8.967 -0.5295 1.5500 N

ATOM 201 HE ARG 12 32.721 -2.606 -8.247 0.3456 1.3000 H

ATOM 202 CZ ARG 12 34.533 -2.522 -9.156 0.8076 1.7000 C

ATOM 203 NH1 ARG 12 35.313 -3.062 -10.068 -0.8627 1.5500 N

ATOM 204 HH11 ARG 12 36.242 -2.699 -10.222 0.4478 1.3000 H

ATOM 205 HH12 ARG 12 34.983 -3.845 -10.612 0.4478 1.3000 H

ATOM 206 NH2 ARG 12 34.965 -1.505 -8.431 -0.8627 1.5500 N

ATOM 207 HH21 ARG 12 35.876 -1.111 -8.605 0.4478 1.3000 H

ATOM 208 HH22 ARG 12 34.390 -1.145 -7.685 0.4478 1.3000 H

ATOM 209 C ARG 12 28.104 -2.275 -9.995 0.7341 1.7000 C

ATOM 210 O ARG 12 27.714 -1.625 -9.024 -0.5894 1.5000 O

ATOM 211 N ALA 13 27.775 -1.960 -11.253 -0.4157 1.5500 N

ATOM 212 H ALA 13 28.107 -2.526 -12.021 0.2719 1.3000 H

ATOM 213 CA ALA 13 26.904 -0.833 -11.546 0.0337 1.7000 C

ATOM 214 HA ALA 13 27.345 0.073 -11.127 0.0823 1.2000 H

ATOM 215 CB ALA 13 26.750 -0.648 -13.044 -0.1825 1.7000 C

ATOM 216 HB1 ALA 13 26.137 0.232 -13.240 0.0603 1.2000 H

ATOM 217 HB2 ALA 13 27.730 -0.502 -13.500 0.0603 1.2000 H

ATOM 218 HB3 ALA 13 26.273 -1.516 -13.496 0.0603 1.2000 H

ATOM 219 C ALA 13 25.535 -1.049 -10.920 0.5973 1.7000 C

ATOM 220 O ALA 13 24.947 -0.125 -10.352 -0.5679 1.5000 O

ATOM 221 N LEU 14 25.043 -2.292 -10.984 -0.4157 1.5500 N

ATOM 222 H LEU 14 25.559 -3.026 -11.449 0.2719 1.3000 H

ATOM 223 CA LEU 14 23.734 -2.595 -10.431 -0.0518 1.7000 C

ATOM 224 HA LEU 14 23.000 -1.899 -10.835 0.0922 1.2000 H

ATOM 225 CB LEU 14 23.342 -4.030 -10.791 -0.1102 1.7000 C

ATOM 226 HB2 LEU 14 24.125 -4.706 -10.448 0.0457 1.2000 H

ATOM 227 HB3 LEU 14 22.430 -4.286 -10.249 0.0457 1.2000 H

ATOM 228 CG LEU 14 23.103 -4.265 -12.307 0.3531 1.7000 C

ATOM 229 HG LEU 14 23.970 -3.952 -12.875 -0.0361 1.2000 H

ATOM 230 CD1 LEU 14 22.880 -5.746 -12.572 -0.4121 1.7000 C

ATOM 231 HD11 LEU 14 22.740 -5.911 -13.641 0.1000 1.2000 H

ATOM 232 HD12 LEU 14 23.745 -6.320 -12.244 0.1000 1.2000 H

ATOM 233 HD13 LEU 14 21.994 -6.093 -12.039 0.1000 1.2000 H

ATOM 234 CD2 LEU 14 21.943 -3.433 -12.772 -0.4121 1.7000 C

ATOM 235 HD21 LEU 14 21.702 -3.692 -13.804 0.1000 1.2000 H

ATOM 236 HD22 LEU 14 21.067 -3.619 -12.150 0.1000 1.2000 H

ATOM 237 HD23 LEU 14 22.195 -2.375 -12.749 0.1000 1.2000 H

ATOM 238 C LEU 14 23.779 -2.380 -8.923 0.5973 1.7000 C

ATOM 239 O LEU 14 22.864 -1.788 -8.342 -0.5679 1.5000 O

ATOM 240 N ILE 15 24.888 -2.783 -8.300 -0.4157 1.5500 N

ATOM 241 H ILE 15 25.622 -3.247 -8.817 0.2719 1.3000 H

ATOM 242 CA ILE 15 25.061 -2.563 -6.877 -0.0597 1.7000 C

ATOM 243 HA ILE 15 24.206 -2.998 -6.357 0.0869 1.2000 H

ATOM 244 CB ILE 15 26.324 -3.232 -6.343 0.1303 1.7000 C

ATOM 245 HB ILE 15 27.152 -2.993 -7.005 0.0187 1.2000 H

ATOM 246 CG2 ILE 15 26.641 -2.642 -4.993 -0.3204 1.7000 C

ATOM 247 HG21 ILE 15 27.470 -3.186 -4.554 0.0882 1.2000 H

ATOM 248 HG22 ILE 15 26.987 -1.611 -5.063 0.0882 1.2000 H

ATOM 249 HG23 ILE 15 25.780 -2.713 -4.328 0.0882 1.2000 H

ATOM 250 CG1 ILE 15 26.137 -4.789 -6.362 -0.0430 1.7000 C

ATOM 251 HG12 ILE 15 25.414 -5.062 -5.592 0.0236 1.2000 H

ATOM 252 HG13 ILE 15 25.717 -5.099 -7.316 0.0236 1.2000 H

ATOM 253 CD1 ILE 15 27.407 -5.601 -6.137 -0.0660 1.7000 C

ATOM 254 HD11 ILE 15 27.183 -6.663 -6.239 0.0186 1.2000 H

ATOM 255 HD12 ILE 15 28.151 -5.318 -6.880 0.0186 1.2000 H

ATOM 256 HD13 ILE 15 27.805 -5.421 -5.142 0.0186 1.2000 H

ATOM 257 C ILE 15 25.091 -1.090 -6.542 0.5973 1.7000 C

ATOM 258 O ILE 15 24.454 -0.667 -5.579 -0.5679 1.5000 O

ATOM 259 N LYS 16 25.822 -0.295 -7.319 -0.3479 1.5500 N

ATOM 260 H LYS 16 26.329 -0.670 -8.107 0.2747 1.3000 H

ATOM 261 CA LYS 16 25.889 1.128 -7.030 -0.2400 1.7000 C

ATOM 262 HA LYS 16 26.258 1.266 -6.012 0.1426 1.2000 H

ATOM 263 CB LYS 16 26.846 1.832 -7.989 -0.0094 1.7000 C

ATOM 264 HB2 LYS 16 26.577 1.575 -9.015 0.0362 1.2000 H

ATOM 265 HB3 LYS 16 26.714 2.909 -7.871 0.0362 1.2000 H

ATOM 266 CG LYS 16 28.320 1.517 -7.761 0.0187 1.7000 C

ATOM 267 HG2 LYS 16 28.600 1.836 -6.756 0.0103 1.2000 H

ATOM 268 HG3 LYS 16 28.493 0.446 -7.839 0.0103 1.2000 H

ATOM 269 CD LYS 16 29.190 2.243 -8.776 -0.0479 1.7000 C

ATOM 270 HD2 LYS 16 28.903 1.928 -9.781 0.0621 1.2000 H

ATOM 271 HD3 LYS 16 29.027 3.318 -8.683 0.0621 1.2000 H

ATOM 272 CE LYS 16 30.663 1.945 -8.567 -0.0143 1.7000 C

ATOM 273 HE2 LYS 16 30.954 2.261 -7.563 0.1135 1.2000 H

ATOM 274 HE3 LYS 16 30.815 0.868 -8.653 0.1135 1.2000 H

ATOM 275 NZ LYS 16 31.514 2.644 -9.576 -0.3854 1.5500 N

ATOM 276 HZ1 LYS 16 31.467 3.643 -9.425 0.3400 1.3000 H

ATOM 277 HZ2 LYS 16 32.474 2.344 -9.488 0.3400 1.3000 H

ATOM 278 HZ3 LYS 16 31.183 2.436 -10.508 0.3400 1.3000 H

ATOM 279 C LYS 16 24.511 1.772 -7.115 0.7341 1.7000 C

ATOM 280 O LYS 16 24.170 2.627 -6.293 -0.5894 1.5000 O

ATOM 281 N ARG 17 23.687 1.339 -8.075 -0.3479 1.5500 N

ATOM 282 H ARG 17 23.988 0.623 -8.722 0.2747 1.3000 H

ATOM 283 CA ARG 17 22.345 1.897 -8.198 -0.2637 1.7000 C

ATOM 284 HA ARG 17 22.403 2.984 -8.281 0.1560 1.2000 H

ATOM 285 CB ARG 17 21.662 1.336 -9.434 -0.0007 1.7000 C

ATOM 286 HB2 ARG 17 21.700 0.247 -9.402 0.0327 1.2000 H

ATOM 287 HB3 ARG 17 20.611 1.630 -9.412 0.0327 1.2000 H

ATOM 288 CG ARG 17 22.257 1.820 -10.754 0.0390 1.7000 C

ATOM 289 HG2 ARG 17 22.070 2.890 -10.854 0.0285 1.2000 H

ATOM 290 HG3 ARG 17 23.333 1.671 -10.756 0.0285 1.2000 H

ATOM 291 CD ARG 17 21.664 1.114 -11.917 0.0486 1.7000 C

ATOM 292 HD2 ARG 17 21.759 0.039 -11.754 0.0687 1.2000 H

ATOM 293 HD3 ARG 17 20.605 1.368 -11.988 0.0687 1.2000 H

ATOM 294 NE ARG 17 22.329 1.460 -13.162 -0.5295 1.5500 N

ATOM 295 HE ARG 17 22.997 2.215 -13.133 0.3456 1.3000 H

ATOM 296 CZ ARG 17 22.122 0.832 -14.333 0.8076 1.7000 C

ATOM 297 NH1 ARG 17 21.248 -0.147 -14.403 -0.8627 1.5500 N

ATOM 298 HH11 ARG 17 20.744 -0.431 -13.577 0.4478 1.3000 H

ATOM 299 HH12 ARG 17 21.067 -0.600 -15.286 0.4478 1.3000 H

ATOM 300 NH2 ARG 17 22.793 1.197 -15.411 -0.8627 1.5500 N

ATOM 301 HH21 ARG 17 22.629 0.731 -16.290 0.4478 1.3000 H

ATOM 302 HH22 ARG 17 23.454 1.957 -15.361 0.4478 1.3000 H

ATOM 303 C ARG 17 21.537 1.550 -6.948 0.7341 1.7000 C

ATOM 304 O ARG 17 20.844 2.400 -6.380 -0.5894 1.5000 O

ATOM 305 N ILE 18 21.693 0.318 -6.464 -0.4157 1.5500 N

ATOM 306 H ILE 18 22.292 -0.346 -6.935 0.2719 1.3000 H

ATOM 307 CA ILE 18 20.987 -0.092 -5.265 -0.0597 1.7000 C

ATOM 308 HA ILE 18 19.920 0.086 -5.404 0.0869 1.2000 H

ATOM 309 CB ILE 18 21.197 -1.591 -4.990 0.1303 1.7000 C

ATOM 310 HB ILE 18 22.262 -1.817 -5.034 0.0187 1.2000 H

ATOM 311 CG2 ILE 18 20.706 -1.951 -3.591 -0.3204 1.7000 C

ATOM 312 HG21 ILE 18 20.745 -3.025 -3.426 0.0882 1.2000 H

ATOM 313 HG22 ILE 18 21.347 -1.499 -2.836 0.0882 1.2000 H

ATOM 314 HG23 ILE 18 19.679 -1.610 -3.450 0.0882 1.2000 H

ATOM 315 CG1 ILE 18 20.487 -2.410 -6.081 -0.0430 1.7000 C

ATOM 316 HG12 ILE 18 19.409 -2.335 -5.928 0.0236 1.2000 H

ATOM 317 HG13 ILE 18 20.698 -2.003 -7.068 0.0236 1.2000 H

ATOM 318 CD1 ILE 18 20.869 -3.872 -6.095 -0.0660 1.7000 C

ATOM 319 HD11 ILE 18 20.282 -4.385 -6.858 0.0186 1.2000 H

ATOM 320 HD12 ILE 18 21.926 -3.974 -6.341 0.0186 1.2000 H

ATOM 321 HD13 ILE 18 20.672 -4.348 -5.137 0.0186 1.2000 H

ATOM 322 C ILE 18 21.446 0.726 -4.075 0.5973 1.7000 C

ATOM 323 O ILE 18 20.620 1.268 -3.339 -0.5679 1.5000 O

ATOM 324 N GLN 19 22.756 0.869 -3.911 -0.4157 1.5500 N

ATOM 325 H GLN 19 23.397 0.437 -4.559 0.2719 1.3000 H

ATOM 326 CA GLN 19 23.291 1.608 -2.785 -0.0031 1.7000 C

ATOM 327 HA GLN 19 22.917 1.164 -1.863 0.0850 1.2000 H

ATOM 328 CB GLN 19 24.806 1.533 -2.776 -0.0036 1.7000 C

ATOM 329 HB2 GLN 19 25.171 1.850 -3.754 0.0171 1.2000 H

ATOM 330 HB3 GLN 19 25.184 2.239 -2.034 0.0171 1.2000 H

ATOM 331 CG GLN 19 25.360 0.174 -2.443 -0.0645 1.7000 C

ATOM 332 HG2 GLN 19 25.036 -0.111 -1.442 0.0352 1.2000 H

ATOM 333 HG3 GLN 19 24.975 -0.576 -3.129 0.0352 1.2000 H

ATOM 334 CD GLN 19 26.842 0.178 -2.532 0.6951 1.7000 C

ATOM 335 OE1 GLN 19 27.433 0.674 -3.499 -0.6086 1.5000 O

ATOM 336 NE2 GLN 19 27.465 -0.339 -1.506 -0.9407 1.5500 N

ATOM 337 HE21 GLN 19 26.947 -0.706 -0.721 0.4251 1.3000 H

ATOM 338 HE22 GLN 19 28.474 -0.326 -1.484 0.4251 1.3000 H

ATOM 339 C GLN 19 22.851 3.056 -2.816 0.5973 1.7000 C

ATOM 340 O GLN 19 22.557 3.645 -1.782 -0.5679 1.5000 O

ATOM 341 N ALA 20 22.779 3.640 -4.008 -0.4157 1.5500 N

ATOM 342 H ALA 20 23.025 3.124 -4.840 0.2719 1.3000 H

ATOM 343 CA ALA 20 22.327 5.015 -4.148 0.0337 1.7000 C

ATOM 344 HA ALA 20 22.983 5.662 -3.564 0.0823 1.2000 H

ATOM 345 CB ALA 20 22.397 5.441 -5.603 -0.1825 1.7000 C

ATOM 346 HB1 ALA 20 22.095 6.485 -5.692 0.0603 1.2000 H

ATOM 347 HB2 ALA 20 23.420 5.340 -5.967 0.0603 1.2000 H

ATOM 348 HB3 ALA 20 21.736 4.829 -6.214 0.0603 1.2000 H

ATOM 349 C ALA 20 20.899 5.171 -3.630 0.5973 1.7000 C

ATOM 350 O ALA 20 20.552 6.199 -3.044 -0.5679 1.5000 O

ATOM 351 N MET 21 20.075 4.143 -3.846 -0.4157 1.5500 N

ATOM 352 H MET 21 20.424 3.325 -4.326 0.2719 1.3000 H

ATOM 353 CA MET 21 18.680 4.140 -3.428 -0.0237 1.7000 C

ATOM 354 HA MET 21 18.283 5.152 -3.525 0.0880 1.2000 H

ATOM 355 CB MET 21 17.893 3.244 -4.377 0.0342 1.7000 C

ATOM 356 HB2 MET 21 18.353 2.257 -4.409 0.0241 1.2000 H

ATOM 357 HB3 MET 21 16.884 3.128 -3.979 0.0241 1.2000 H

ATOM 358 CG MET 21 17.765 3.797 -5.797 0.0018 1.7000 C

ATOM 359 HG2 MET 21 17.349 4.803 -5.736 0.0440 1.2000 H

ATOM 360 HG3 MET 21 18.747 3.864 -6.263 0.0440 1.2000 H

ATOM 361 SD MET 21 16.676 2.810 -6.837 -0.2737 1.8000 S

ATOM 362 CE MET 21 17.725 1.388 -7.143 -0.0536 1.7000 C

ATOM 363 HE1 MET 21 17.213 0.703 -7.819 0.0684 1.2000 H

ATOM 364 HE2 MET 21 18.656 1.714 -7.603 0.0684 1.2000 H

ATOM 365 HE3 MET 21 17.935 0.874 -6.206 0.0684 1.2000 H

ATOM 366 C MET 21 18.445 3.698 -1.975 0.5973 1.7000 C

ATOM 367 O MET 21 17.452 4.111 -1.361 -0.5679 1.5000 O

ATOM 368 N ILE 22 19.359 2.909 -1.394 -0.4157 1.5500 N

ATOM 369 H ILE 22 20.174 2.601 -1.906 0.2719 1.3000 H

ATOM 370 CA ILE 22 19.122 2.409 -0.038 -0.0597 1.7000 C

ATOM 371 HA ILE 22 18.223 1.794 -0.103 0.0869 1.2000 H

ATOM 372 CB ILE 22 20.238 1.478 0.505 0.1303 1.7000 C

ATOM 373 HB ILE 22 21.205 1.938 0.331 0.0187 1.2000 H

ATOM 374 CG2 ILE 22 20.064 1.326 1.973 -0.3204 1.7000 C

ATOM 375 HG21 ILE 22 20.770 0.599 2.364 0.0882 1.2000 H

ATOM 376 HG22 ILE 22 20.284 2.241 2.519 0.0882 1.2000 H

ATOM 377 HG23 ILE 22 19.054 0.996 2.204 0.0882 1.2000 H

ATOM 378 CG1 ILE 22 20.253 0.142 -0.213 -0.0430 1.7000 C

ATOM 379 HG12 ILE 22 19.347 -0.417 0.001 0.0236 1.2000 H

ATOM 380 HG13 ILE 22 20.259 0.303 -1.277 0.0236 1.2000 H

ATOM 381 CD1 ILE 22 21.478 -0.696 0.125 -0.0660 1.7000 C

ATOM 382 HD11 ILE 22 21.502 -1.572 -0.520 0.0186 1.2000 H

ATOM 383 HD12 ILE 22 22.390 -0.122 -0.032 0.0186 1.2000 H

ATOM 384 HD13 ILE 22 21.433 -1.043 1.155 0.0186 1.2000 H

ATOM 385 C ILE 22 18.812 3.473 1.027 0.5973 1.7000 C

ATOM 386 O ILE 22 17.865 3.275 1.780 -0.5679 1.5000 O

ATOM 387 N PRO 23 19.549 4.599 1.145 -0.2548 1.5500 N

ATOM 388 CD PRO 23 20.764 4.826 0.340 0.0192 1.7000 C

ATOM 389 HD2 PRO 23 20.502 5.213 -0.644 0.0391 1.2000 H

ATOM 390 HD3 PRO 23 21.393 3.948 0.284 0.0391 1.2000 H

ATOM 391 CG PRO 23 21.484 5.870 1.130 0.0189 1.7000 C

ATOM 392 HG2 PRO 23 22.127 6.479 0.493 0.0213 1.2000 H

ATOM 393 HG3 PRO 23 22.076 5.390 1.912 0.0213 1.2000 H

ATOM 394 CB PRO 23 20.394 6.686 1.759 -0.0070 1.7000 C

ATOM 395 HB2 PRO 23 20.031 7.412 1.029 0.0253 1.2000 H

ATOM 396 HB3 PRO 23 20.746 7.200 2.654 0.0253 1.2000 H

ATOM 397 CA PRO 23 19.290 5.675 2.092 -0.0266 1.7000 C

ATOM 398 HA PRO 23 19.423 5.297 3.107 0.0641 1.2000 H

ATOM 399 C PRO 23 17.910 6.336 1.952 0.5896 1.7000 C

ATOM 400 O PRO 23 17.486 7.058 2.857 -0.5748 1.5000 O

ATOM 401 N LYS 24 17.250 6.172 0.797 -0.3479 1.5500 N

ATOM 402 H LYS 24 17.637 5.582 0.074 0.2747 1.3000 H

ATOM 403 CA LYS 24 15.963 6.815 0.561 -0.2400 1.7000 C

ATOM 404 HA LYS 24 15.732 7.512 1.364 0.1426 1.2000 H

ATOM 405 CB LYS 24 16.030 7.605 -0.745 -0.0094 1.7000 C

ATOM 406 HB2 LYS 24 16.235 6.912 -1.563 0.0362 1.2000 H

ATOM 407 HB3 LYS 24 15.056 8.060 -0.935 0.0362 1.2000 H

ATOM 408 CG LYS 24 17.097 8.711 -0.774 0.0187 1.7000 C

ATOM 409 HG2 LYS 24 18.086 8.286 -0.599 0.0103 1.2000 H

ATOM 410 HG3 LYS 24 17.095 9.159 -1.769 0.0103 1.2000 H

ATOM 411 CD LYS 24 16.817 9.806 0.255 -0.0479 1.7000 C

ATOM 412 HD2 LYS 24 15.816 10.208 0.091 0.0621 1.2000 H

ATOM 413 HD3 LYS 24 16.870 9.387 1.260 0.0621 1.2000 H

ATOM 414 CE LYS 24 17.836 10.942 0.155 -0.0143 1.7000 C

ATOM 415 HE2 LYS 24 18.837 10.531 0.301 0.1135 1.2000 H

ATOM 416 HE3 LYS 24 17.781 11.377 -0.844 0.1135 1.2000 H

ATOM 417 NZ LYS 24 17.588 12.007 1.172 -0.3854 1.5500 N

ATOM 418 HZ1 LYS 24 18.265 12.749 1.063 0.3400 1.3000 H

ATOM 419 HZ2 LYS 24 16.657 12.382 1.052 0.3400 1.3000 H

ATOM 420 HZ3 LYS 24 17.672 11.615 2.100 0.3400 1.3000 H

ATOM 421 C LYS 24 14.773 5.851 0.498 0.7341 1.7000 C

ATOM 422 O LYS 24 13.636 6.252 0.763 -0.5894 1.5000 O

ATOM 423 N GLY 25 15.015 4.601 0.104 -0.4157 1.5500 N

ATOM 424 H GLY 25 15.958 4.303 -0.099 0.2719 1.3000 H

ATOM 425 CA GLY 25 13.917 3.663 -0.103 -0.0252 1.7000 C

ATOM 426 HA2 GLY 25 13.091 4.166 -0.608 0.0698 1.2000 H

ATOM 427 HA3 GLY 25 14.268 2.870 -0.763 0.0698 1.2000 H

ATOM 428 C GLY 25 13.397 3.021 1.182 0.5973 1.7000 C

ATOM 429 O GLY 25 13.863 3.315 2.282 -0.5679 1.5000 O

ATOM 430 N ALA 26 12.402 2.142 1.023 -0.4157 1.5500 N

ATOM 431 H ALA 26 12.057 1.948 0.094 0.2719 1.3000 H

ATOM 432 CA ALA 26 11.748 1.461 2.143 0.0337 1.7000 C

ATOM 433 HA ALA 26 12.411 1.469 3.010 0.0823 1.2000 H

ATOM 434 CB ALA 26 10.485 2.184 2.532 -0.1825 1.7000 C

ATOM 435 HB1 ALA 26 10.040 1.681 3.386 0.0603 1.2000 H

ATOM 436 HB2 ALA 26 10.727 3.207 2.823 0.0603 1.2000 H

ATOM 437 HB3 ALA 26 9.780 2.214 1.701 0.0603 1.2000 H

ATOM 438 C ALA 26 11.445 0.001 1.812 0.5973 1.7000 C

ATOM 439 O ALA 26 10.504 -0.183 1.043 -0.5679 1.5000 O

ATOM 440 N LEU 27 12.564 -0.694 1.659 -0.4157 1.5500 N

ATOM 441 H LEU 27 13.440 -0.243 1.881 0.2719 1.3000 H

ATOM 442 CA LEU 27 12.494 -2.119 1.965 -0.0518 1.7000 C

ATOM 443 HA LEU 27 12.062 -2.596 1.086 0.0922 1.2000 H

ATOM 444 CB LEU 27 13.889 -2.681 2.182 -0.1102 1.7000 C

ATOM 445 HB2 LEU 27 14.486 -2.488 1.288 0.0457 1.2000 H

ATOM 446 HB3 LEU 27 14.339 -2.132 3.007 0.0457 1.2000 H

ATOM 447 CG LEU 27 13.941 -4.151 2.513 0.3531 1.7000 C

ATOM 448 HG LEU 27 13.378 -4.364 3.422 -0.0361 1.2000 H

ATOM 449 CD1 LEU 27 13.365 -4.948 1.367 -0.4121 1.7000 C

ATOM 450 HD11 LEU 27 13.468 -6.013 1.574 0.1000 1.2000 H

ATOM 451 HD12 LEU 27 12.306 -4.742 1.231 0.1000 1.2000 H

ATOM 452 HD13 LEU 27 13.893 -4.720 0.440 0.1000 1.2000 H

ATOM 453 CD2 LEU 27 15.356 -4.534 2.759 -0.4121 1.7000 C

ATOM 454 HD21 LEU 27 15.397 -5.562 3.110 0.1000 1.2000 H

ATOM 455 HD22 LEU 27 15.945 -4.436 1.846 0.1000 1.2000 H

ATOM 456 HD23 LEU 27 15.787 -3.896 3.526 0.1000 1.2000 H

ATOM 457 C LEU 27 11.627 -2.478 3.166 0.5973 1.7000 C

ATOM 458 O LEU 27 10.832 -3.402 3.053 -0.5679 1.5000 O

ATOM 459 N ALA 28 11.706 -1.750 4.287 -0.4157 1.5500 N

ATOM 460 H ALA 28 12.366 -0.992 4.385 0.2719 1.3000 H

ATOM 461 CA ALA 28 10.851 -2.125 5.416 0.0337 1.7000 C

ATOM 462 HA ALA 28 11.125 -3.124 5.760 0.0823 1.2000 H

ATOM 463 CB ALA 28 11.003 -1.150 6.562 -0.1825 1.7000 C

ATOM 464 HB1 ALA 28 10.394 -1.477 7.405 0.0603 1.2000 H

ATOM 465 HB2 ALA 28 12.035 -1.076 6.878 0.0603 1.2000 H

ATOM 466 HB3 ALA 28 10.673 -0.156 6.257 0.0603 1.2000 H

ATOM 467 C ALA 28 9.391 -2.120 4.993 0.5973 1.7000 C

ATOM 468 O ALA 28 8.623 -3.003 5.370 -0.5679 1.5000 O

ATOM 469 N VAL 29 9.017 -1.135 4.174 -0.4157 1.5500 N

ATOM 470 H VAL 29 9.685 -0.435 3.888 0.2719 1.3000 H

ATOM 471 CA VAL 29 7.656 -1.036 3.688 -0.0875 1.7000 C

ATOM 472 HA VAL 29 6.981 -1.198 4.529 0.0969 1.2000 H

ATOM 473 CB VAL 29 7.354 0.338 3.111 0.2985 1.7000 C

ATOM 474 HB VAL 29 8.102 0.605 2.366 -0.0297 1.2000 H

ATOM 475 CG1 VAL 29 5.995 0.319 2.452 -0.3192 1.7000 C

ATOM 476 HG11 VAL 29 5.702 1.339 2.199 0.0791 1.2000 H

ATOM 477 HG12 VAL 29 6.021 -0.249 1.522 0.0791 1.2000 H

ATOM 478 HG13 VAL 29 5.244 -0.100 3.124 0.0791 1.2000 H

ATOM 479 CG2 VAL 29 7.364 1.356 4.247 -0.3192 1.7000 C

ATOM 480 HG21 VAL 29 7.208 2.357 3.842 0.0791 1.2000 H

ATOM 481 HG22 VAL 29 6.565 1.136 4.957 0.0791 1.2000 H

ATOM 482 HG23 VAL 29 8.313 1.345 4.782 0.0791 1.2000 H

ATOM 483 C VAL 29 7.366 -2.095 2.659 0.5973 1.7000 C

ATOM 484 O VAL 29 6.346 -2.770 2.747 -0.5679 1.5000 O

ATOM 485 N ALA 30 8.270 -2.310 1.706 -0.4157 1.5500 N

ATOM 486 H ALA 30 9.117 -1.764 1.639 0.2719 1.3000 H

ATOM 487 CA ALA 30 7.986 -3.344 0.730 0.0337 1.7000 C

ATOM 488 HA ALA 30 7.101 -3.062 0.157 0.0823 1.2000 H

ATOM 489 CB ALA 30 9.156 -3.490 -0.221 -0.1825 1.7000 C

ATOM 490 HB1 ALA 30 8.928 -4.253 -0.966 0.0603 1.2000 H

ATOM 491 HB2 ALA 30 9.337 -2.542 -0.730 0.0603 1.2000 H

ATOM 492 HB3 ALA 30 10.054 -3.781 0.324 0.0603 1.2000 H

ATOM 493 C ALA 30 7.724 -4.678 1.428 0.5973 1.7000 C

ATOM 494 O ALA 30 6.780 -5.391 1.080 -0.5679 1.5000 O

ATOM 495 N VAL 31 8.501 -4.988 2.464 -0.4157 1.5500 N

ATOM 496 H VAL 31 9.248 -4.374 2.753 0.2719 1.3000 H

ATOM 497 CA VAL 31 8.288 -6.232 3.178 -0.0875 1.7000 C

ATOM 498 HA VAL 31 8.178 -7.035 2.447 0.0969 1.2000 H

ATOM 499 CB VAL 31 9.467 -6.588 4.080 0.2985 1.7000 C

ATOM 500 HB VAL 31 9.682 -5.755 4.743 -0.0297 1.2000 H

ATOM 501 CG1 VAL 31 9.102 -7.831 4.895 -0.3192 1.7000 C

ATOM 502 HG11 VAL 31 9.969 -8.145 5.477 0.0791 1.2000 H

ATOM 503 HG12 VAL 31 8.290 -7.641 5.592 0.0791 1.2000 H

ATOM 504 HG13 VAL 31 8.818 -8.648 4.230 0.0791 1.2000 H

ATOM 505 CG2 VAL 31 10.697 -6.833 3.249 -0.3192 1.7000 C

ATOM 506 HG21 VAL 31 11.539 -7.060 3.903 0.0791 1.2000 H

ATOM 507 HG22 VAL 31 10.534 -7.677 2.577 0.0791 1.2000 H

ATOM 508 HG23 VAL 31 10.947 -5.956 2.659 0.0791 1.2000 H

ATOM 509 C VAL 31 6.989 -6.158 3.981 0.5973 1.7000 C

ATOM 510 O VAL 31 6.216 -7.112 3.986 -0.5679 1.5000 O

ATOM 511 N ALA 32 6.722 -5.024 4.635 -0.4157 1.5500 N

ATOM 512 H ALA 32 7.375 -4.254 4.602 0.2719 1.3000 H

ATOM 513 CA ALA 32 5.492 -4.864 5.403 0.0337 1.7000 C

ATOM 514 HA ALA 32 5.482 -5.616 6.192 0.0823 1.2000 H

ATOM 515 CB ALA 32 5.441 -3.490 6.041 -0.1825 1.7000 C

ATOM 516 HB1 ALA 32 4.522 -3.386 6.619 0.0603 1.2000 H

ATOM 517 HB2 ALA 32 6.291 -3.361 6.712 0.0603 1.2000 H

ATOM 518 HB3 ALA 32 5.459 -2.716 5.276 0.0603 1.2000 H

ATOM 519 C ALA 32 4.266 -5.075 4.506 0.5973 1.7000 C

ATOM 520 O ALA 32 3.216 -5.536 4.967 -0.5679 1.5000 O

ATOM 521 N GLN 33 4.373 -4.680 3.232 -0.4157 1.5500 N

ATOM 522 H GLN 33 5.244 -4.289 2.902 0.2719 1.3000 H

ATOM 523 CA GLN 33 3.306 -4.901 2.265 -0.0031 1.7000 C

ATOM 524 HA GLN 33 2.360 -4.573 2.698 0.0850 1.2000 H

ATOM 525 CB GLN 33 3.562 -4.084 0.997 -0.0036 1.7000 C

ATOM 526 HB2 GLN 33 4.559 -4.312 0.625 0.0171 1.2000 H

ATOM 527 HB3 GLN 33 2.842 -4.385 0.234 0.0171 1.2000 H

ATOM 528 CG GLN 33 3.427 -2.579 1.214 -0.0645 1.7000 C

ATOM 529 HG2 GLN 33 2.381 -2.360 1.436 0.0352 1.2000 H

ATOM 530 HG3 GLN 33 4.010 -2.258 2.074 0.0352 1.2000 H

ATOM 531 CD GLN 33 3.854 -1.759 0.011 0.6951 1.7000 C

ATOM 532 OE1 GLN 33 4.552 -2.238 -0.889 -0.6086 1.5000 O

ATOM 533 NE2 GLN 33 3.437 -0.496 -0.010 -0.9407 1.5500 N

ATOM 534 HE21 GLN 33 2.884 -0.140 0.754 0.4251 1.3000 H

ATOM 535 HE22 GLN 33 3.691 0.098 -0.782 0.4251 1.3000 H

ATOM 536 C GLN 33 3.196 -6.402 1.964 0.5973 1.7000 C

ATOM 537 O GLN 33 2.089 -6.950 1.890 -0.5679 1.5000 O

ATOM 538 N VAL 34 4.346 -7.091 1.870 -0.4157 1.5500 N

ATOM 539 H VAL 34 5.234 -6.614 1.956 0.2719 1.3000 H

ATOM 540 CA VAL 34 4.356 -8.536 1.640 -0.0875 1.7000 C

ATOM 541 HA VAL 34 3.810 -8.745 0.719 0.0969 1.2000 H

ATOM 542 CB VAL 34 5.770 -9.134 1.518 0.2985 1.7000 C

ATOM 543 HB VAL 34 6.392 -8.853 2.361 -0.0297 1.2000 H

ATOM 544 CG1 VAL 34 5.660 -10.652 1.512 -0.3192 1.7000 C

ATOM 545 HG11 VAL 34 6.622 -11.081 1.230 0.0791 1.2000 H

ATOM 546 HG12 VAL 34 5.421 -11.039 2.503 0.0791 1.2000 H

ATOM 547 HG13 VAL 34 4.911 -10.983 0.790 0.0791 1.2000 H

ATOM 548 CG2 VAL 34 6.434 -8.652 0.250 -0.3192 1.7000 C

ATOM 549 HG21 VAL 34 7.477 -8.971 0.237 0.0791 1.2000 H

ATOM 550 HG22 VAL 34 5.925 -9.071 -0.619 0.0791 1.2000 H

ATOM 551 HG23 VAL 34 6.399 -7.573 0.173 0.0791 1.2000 H

ATOM 552 C VAL 34 3.652 -9.238 2.775 0.5973 1.7000 C

ATOM 553 O VAL 34 2.856 -10.141 2.546 -0.5679 1.5000 O

ATOM 554 N CYX 35 3.868 -8.771 4.005 -0.4157 1.5500 N

ATOM 555 H CYX 35 4.517 -8.007 4.131 0.2719 1.3000 H

ATOM 556 CA CYX 35 3.292 -9.371 5.207 0.0429 1.7000 C

ATOM 557 HA CYX 35 3.731 -10.361 5.335 0.0766 1.2000 H

ATOM 558 CB CYX 35 3.618 -8.533 6.440 -0.0790 1.7000 C

ATOM 559 HB2 CYX 35 3.274 -7.515 6.286 0.0910 1.2000 H

ATOM 560 HB3 CYX 35 3.069 -8.934 7.292 0.0910 1.2000 H

ATOM 561 SG CYX 35 5.369 -8.505 6.882 -0.1081 1.8000 S

ATOM 562 C CYX 35 1.773 -9.544 5.130 0.5973 1.7000 C

ATOM 563 O CYX 35 1.188 -10.269 5.925 -0.5679 1.5000 O

ATOM 564 N ARG 36 1.095 -8.939 4.165 -0.3479 1.5500 N

ATOM 565 H ARG 36 1.576 -8.366 3.486 0.2747 1.3000 H

ATOM 566 CA ARG 36 -0.344 -9.124 4.047 -0.2637 1.7000 C

ATOM 567 HA ARG 36 -0.816 -8.869 4.997 0.1560 1.2000 H

ATOM 568 CB ARG 36 -0.899 -8.213 2.972 -0.0007 1.7000 C

ATOM 569 HB2 ARG 36 -0.350 -8.400 2.047 0.0327 1.2000 H

ATOM 570 HB3 ARG 36 -1.944 -8.473 2.796 0.0327 1.2000 H

ATOM 571 CG ARG 36 -0.842 -6.730 3.325 0.0390 1.7000 C

ATOM 572 HG2 ARG 36 -1.497 -6.552 4.179 0.0285 1.2000 H

ATOM 573 HG3 ARG 36 0.170 -6.445 3.615 0.0285 1.2000 H

ATOM 574 CD ARG 36 -1.287 -5.864 2.199 0.0486 1.7000 C

ATOM 575 HD2 ARG 36 -2.283 -6.177 1.883 0.0687 1.2000 H

ATOM 576 HD3 ARG 36 -1.342 -4.834 2.555 0.0687 1.2000 H

ATOM 577 NE ARG 36 -0.369 -5.921 1.065 -0.5295 1.5500 N

ATOM 578 HE ARG 36 0.469 -6.472 1.176 0.3456 1.3000 H

ATOM 579 CZ ARG 36 -0.589 -5.310 -0.122 0.8076 1.7000 C

ATOM 580 NH1 ARG 36 -1.688 -4.604 -0.297 -0.8627 1.5500 N

ATOM 581 HH11 ARG 36 -1.868 -4.157 -1.183 0.4478 1.3000 H

ATOM 582 HH12 ARG 36 -2.357 -4.527 0.454 0.4478 1.3000 H

ATOM 583 NH2 ARG 36 0.285 -5.411 -1.112 -0.8627 1.5500 N

ATOM 584 HH21 ARG 36 0.114 -4.947 -1.991 0.4478 1.3000 H

ATOM 585 HH22 ARG 36 1.126 -5.955 -0.984 0.4478 1.3000 H

ATOM 586 C ARG 36 -0.698 -10.594 3.729 0.7341 1.7000 C

ATOM 587 O ARG 36 -1.862 -10.987 3.818 -0.5894 1.5000 O

ATOM 588 N VAL 37 0.312 -11.402 3.357 -0.4157 1.5500 N

ATOM 589 H VAL 37 1.244 -11.019 3.285 0.2719 1.3000 H

ATOM 590 CA VAL 37 0.139 -12.812 3.019 -0.0875 1.7000 C

ATOM 591 HA VAL 37 -0.858 -12.964 2.603 0.0969 1.2000 H

ATOM 592 CB VAL 37 1.180 -13.270 1.980 0.2985 1.7000 C

ATOM 593 HB VAL 37 0.927 -14.278 1.647 -0.0297 1.2000 H

ATOM 594 CG1 VAL 37 1.101 -12.378 0.749 -0.3192 1.7000 C

ATOM 595 HG11 VAL 37 1.779 -12.760 -0.015 0.0791 1.2000 H

ATOM 596 HG12 VAL 37 0.087 -12.396 0.349 0.0791 1.2000 H

ATOM 597 HG13 VAL 37 1.376 -11.350 0.969 0.0791 1.2000 H

ATOM 598 CG2 VAL 37 2.572 -13.329 2.629 -0.3192 1.7000 C

ATOM 599 HG21 VAL 37 3.334 -13.340 1.850 0.0791 1.2000 H

ATOM 600 HG22 VAL 37 2.756 -12.484 3.288 0.0791 1.2000 H

ATOM 601 HG23 VAL 37 2.684 -14.248 3.205 0.0791 1.2000 H

ATOM 602 C VAL 37 0.310 -13.751 4.223 0.5973 1.7000 C

ATOM 603 O VAL 37 0.247 -14.970 4.051 -0.5679 1.5000 O

ATOM 604 N VAL 38 0.525 -13.221 5.435 -0.4157 1.5500 N

ATOM 605 H VAL 38 0.556 -12.221 5.576 0.2719 1.3000 H

ATOM 606 CA VAL 38 0.709 -14.125 6.572 -0.0875 1.7000 C

ATOM 607 HA VAL 38 0.860 -15.158 6.265 0.0969 1.2000 H

ATOM 608 CB VAL 38 1.908 -13.700 7.469 0.2985 1.7000 C

ATOM 609 HB VAL 38 2.100 -14.495 8.187 -0.0297 1.2000 H

ATOM 610 CG1 VAL 38 3.167 -13.597 6.623 -0.3192 1.7000 C

ATOM 611 HG11 VAL 38 4.032 -13.451 7.271 0.0791 1.2000 H

ATOM 612 HG12 VAL 38 3.309 -14.516 6.053 0.0791 1.2000 H

ATOM 613 HG13 VAL 38 3.101 -12.752 5.937 0.0791 1.2000 H

ATOM 614 CG2 VAL 38 1.591 -12.474 8.251 -0.3192 1.7000 C

ATOM 615 HG21 VAL 38 2.512 -11.978 8.555 0.0791 1.2000 H

ATOM 616 HG22 VAL 38 0.972 -11.779 7.700 0.0791 1.2000 H

ATOM 617 HG23 VAL 38 1.048 -12.761 9.152 0.0791 1.2000 H

ATOM 618 C VAL 38 -0.603 -13.984 7.346 0.5973 1.7000 C

ATOM 619 O VAL 38 -1.372 -13.084 7.032 -0.5679 1.5000 O

ATOM 620 N PRO 39 -0.966 -14.875 8.282 -0.2548 1.5500 N

ATOM 621 CD PRO 39 -0.142 -16.053 8.620 0.0192 1.7000 C

ATOM 622 HD2 PRO 39 0.686 -15.762 9.266 0.0391 1.2000 H

ATOM 623 HD3 PRO 39 0.202 -16.594 7.738 0.0391 1.2000 H

ATOM 624 CG PRO 39 -1.132 -16.893 9.406 0.0189 1.7000 C

ATOM 625 HG2 PRO 39 -0.628 -17.521 10.141 0.0213 1.2000 H

ATOM 626 HG3 PRO 39 -1.713 -17.510 8.718 0.0213 1.2000 H

ATOM 627 CB PRO 39 -2.049 -15.875 10.072 -0.0070 1.7000 C

ATOM 628 HB2 PRO 39 -1.554 -15.477 10.960 0.0253 1.2000 H

ATOM 629 HB3 PRO 39 -3.008 -16.317 10.343 0.0253 1.2000 H

ATOM 630 CA PRO 39 -2.217 -14.782 9.014 -0.0266 1.7000 C

ATOM 631 HA PRO 39 -3.051 -15.017 8.350 0.0641 1.2000 H

ATOM 632 C PRO 39 -2.369 -13.406 9.632 0.5896 1.7000 C

ATOM 633 O PRO 39 -1.444 -12.893 10.272 -0.5748 1.5000 O

ATOM 634 N LEU 40 -3.547 -12.816 9.488 -0.4157 1.5500 N

ATOM 635 H LEU 40 -4.296 -13.256 8.974 0.2719 1.3000 H

ATOM 636 CA LEU 40 -3.718 -11.472 9.996 -0.0518 1.7000 C

ATOM 637 HA LEU 40 -2.735 -11.097 10.220 0.0922 1.2000 H

ATOM 638 CB LEU 40 -4.275 -10.577 8.884 -0.1102 1.7000 C

ATOM 639 HB2 LEU 40 -5.238 -10.979 8.565 0.0457 1.2000 H

ATOM 640 HB3 LEU 40 -4.453 -9.578 9.277 0.0457 1.2000 H

ATOM 641 CG LEU 40 -3.345 -10.471 7.658 0.3531 1.7000 C

ATOM 642 HG LEU 40 -3.221 -11.455 7.214 -0.0361 1.2000 H

ATOM 643 CD1 LEU 40 -3.979 -9.593 6.607 -0.4121 1.7000 C

ATOM 644 HD11 LEU 40 -3.330 -9.543 5.732 0.1000 1.2000 H

ATOM 645 HD12 LEU 40 -4.939 -10.012 6.304 0.1000 1.2000 H

ATOM 646 HD13 LEU 40 -4.131 -8.587 6.998 0.1000 1.2000 H

ATOM 647 CD2 LEU 40 -1.963 -9.926 8.106 -0.4121 1.7000 C

ATOM 648 HD21 LEU 40 -1.369 -9.692 7.223 0.1000 1.2000 H

ATOM 649 HD22 LEU 40 -2.082 -9.018 8.697 0.1000 1.2000 H

ATOM 650 HD23 LEU 40 -1.409 -10.675 8.668 0.1000 1.2000 H

ATOM 651 C LEU 40 -4.547 -11.377 11.286 0.5973 1.7000 C

ATOM 652 O LEU 40 -5.443 -12.191 11.498 -0.5679 1.5000 O

ATOM 653 N VAL 41 -4.237 -10.398 12.163 -0.4157 1.5500 N

ATOM 654 H VAL 41 -4.770 -10.337 13.018 0.2719 1.3000 H

ATOM 655 CA VAL 41 -3.169 -9.408 11.957 -0.0875 1.7000 C

ATOM 656 HA VAL 41 -2.927 -9.292 10.905 0.0969 1.2000 H

ATOM 657 CB VAL 41 -3.645 -8.019 12.387 0.2985 1.7000 C

ATOM 658 HB VAL 41 -3.879 -8.008 13.453 -0.0297 1.2000 H

ATOM 659 CG1 VAL 41 -2.558 -6.998 12.096 -0.3192 1.7000 C

ATOM 660 HG11 VAL 41 -3.018 -6.027 11.908 0.0791 1.2000 H

ATOM 661 HG12 VAL 41 -1.896 -6.820 12.941 0.0791 1.2000 H

ATOM 662 HG13 VAL 41 -1.990 -7.257 11.203 0.0791 1.2000 H

ATOM 663 CG2 VAL 41 -4.911 -7.667 11.626 -0.3192 1.7000 C

ATOM 664 HG21 VAL 41 -5.241 -6.668 11.914 0.0791 1.2000 H

ATOM 665 HG22 VAL 41 -4.724 -7.671 10.552 0.0791 1.2000 H

ATOM 666 HG23 VAL 41 -5.724 -8.354 11.860 0.0791 1.2000 H

ATOM 667 C VAL 41 -1.872 -9.735 12.697 0.5973 1.7000 C

ATOM 668 O VAL 41 -1.461 -8.993 13.589 -0.5679 1.5000 O

ATOM 669 N ALA 42 -1.117 -10.723 12.194 -0.4157 1.5500 N

ATOM 670 H ALA 42 -1.460 -11.286 11.428 0.2719 1.3000 H

ATOM 671 CA ALA 42 0.212 -11.006 12.707 0.0337 1.7000 C

ATOM 672 HA ALA 42 0.501 -10.262 13.449 0.0823 1.2000 H

ATOM 673 CB ALA 42 0.209 -12.351 13.401 -0.1825 1.7000 C

ATOM 674 HB1 ALA 42 1.178 -12.532 13.866 0.0603 1.2000 H

ATOM 675 HB2 ALA 42 -0.557 -12.365 14.177 0.0603 1.2000 H

ATOM 676 HB3 ALA 42 0.004 -13.145 12.682 0.0603 1.2000 H

ATOM 677 C ALA 42 1.312 -11.022 11.623 0.5973 1.7000 C

ATOM 678 O ALA 42 2.443 -10.936 12.100 -0.5679 1.5000 O

ATOM 679 N GLY 43 1.084 -10.296 10.509 -0.4157 1.5500 N

ATOM 680 H GLY 43 1.683 -10.488 9.720 0.2719 1.3000 H

ATOM 681 CA GLY 43 0.624 -8.908 10.570 -0.0252 1.7000 C

ATOM 682 HA2 GLY 43 0.601 -8.515 9.553 0.0698 1.2000 H

ATOM 683 HA3 GLY 43 -0.372 -8.790 10.969 0.0698 1.2000 H

ATOM 684 C GLY 43 1.624 -8.106 11.353 0.5973 1.7000 C

ATOM 685 O GLY 43 2.741 -7.912 10.891 -0.5679 1.5000 O

ATOM 686 N GLY 44 1.229 -7.646 12.538 -0.4157 1.5500 N

ATOM 687 H GLY 44 0.297 -7.840 12.878 0.2719 1.3000 H

ATOM 688 CA GLY 44 2.109 -6.832 13.365 -0.0252 1.7000 C

ATOM 689 HA2 GLY 44 2.290 -5.883 12.858 0.0698 1.2000 H

ATOM 690 HA3 GLY 44 1.596 -6.627 14.304 0.0698 1.2000 H

ATOM 691 C GLY 44 3.467 -7.491 13.692 0.5973 1.7000 C

ATOM 692 O GLY 44 4.466 -6.790 13.876 -0.5679 1.5000 O

ATOM 693 N ILE 45 3.534 -8.826 13.784 -0.4157 1.5500 N

ATOM 694 H ILE 45 2.727 -9.409 13.613 0.2719 1.3000 H

ATOM 695 CA ILE 45 4.809 -9.447 14.094 -0.0597 1.7000 C

ATOM 696 HA ILE 45 5.309 -8.885 14.884 0.0869 1.2000 H

ATOM 697 CB ILE 45 4.655 -10.895 14.564 0.1303 1.7000 C

ATOM 698 HB ILE 45 4.108 -11.471 13.821 0.0187 1.2000 H

ATOM 699 CG2 ILE 45 6.039 -11.517 14.710 -0.3204 1.7000 C

ATOM 700 HG21 ILE 45 5.966 -12.505 15.162 0.0882 1.2000 H

ATOM 701 HG22 ILE 45 6.511 -11.653 13.737 0.0882 1.2000 H

ATOM 702 HG23 ILE 45 6.674 -10.891 15.339 0.0882 1.2000 H

ATOM 703 CG1 ILE 45 3.889 -10.933 15.881 -0.0430 1.7000 C

ATOM 704 HG12 ILE 45 4.489 -10.495 16.680 0.0236 1.2000 H

ATOM 705 HG13 ILE 45 2.979 -10.341 15.785 0.0236 1.2000 H

ATOM 706 CD1 ILE 45 3.453 -12.318 16.279 -0.0660 1.7000 C

ATOM 707 HD11 ILE 45 2.672 -12.246 17.037 0.0186 1.2000 H

ATOM 708 HD12 ILE 45 3.057 -12.858 15.419 0.0186 1.2000 H

ATOM 709 HD13 ILE 45 4.292 -12.871 16.700 0.0186 1.2000 H

ATOM 710 C ILE 45 5.664 -9.408 12.850 0.5973 1.7000 C

ATOM 711 O ILE 45 6.796 -8.935 12.876 -0.5679 1.5000 O

ATOM 712 N CYX 46 5.095 -9.841 11.734 -0.4157 1.5500 N

ATOM 713 H CYX 46 4.148 -10.191 11.749 0.2719 1.3000 H

ATOM 714 CA CYX 46 5.814 -9.819 10.476 0.0429 1.7000 C

ATOM 715 HA CYX 46 6.668 -10.494 10.545 0.0766 1.2000 H

ATOM 716 CB CYX 46 4.909 -10.294 9.343 -0.0790 1.7000 C

ATOM 717 HB2 CYX 46 4.581 -11.305 9.586 0.0910 1.2000 H

ATOM 718 HB3 CYX 46 4.024 -9.661 9.292 0.0910 1.2000 H

ATOM 719 SG CYX 46 5.689 -10.352 7.690 -0.1081 1.8000 S

ATOM 720 C CYX 46 6.321 -8.413 10.194 0.5973 1.7000 C

ATOM 721 O CYX 46 7.471 -8.224 9.805 -0.5679 1.5000 O

ATOM 722 N GLN 47 5.462 -7.422 10.372 -0.4157 1.5500 N

ATOM 723 H GLN 47 4.529 -7.620 10.699 0.2719 1.3000 H

ATOM 724 CA GLN 47 5.813 -6.047 10.102 -0.0031 1.7000 C

ATOM 725 HA GLN 47 6.222 -5.998 9.092 0.0850 1.2000 H

ATOM 726 CB GLN 47 4.542 -5.217 10.115 -0.0036 1.7000 C

ATOM 727 HB2 GLN 47 4.005 -5.387 11.050 0.0171 1.2000 H

ATOM 728 HB3 GLN 47 4.799 -4.159 10.046 0.0171 1.2000 H

ATOM 729 CG GLN 47 3.666 -5.587 8.919 -0.0645 1.7000 C

ATOM 730 HG2 GLN 47 4.209 -5.307 8.019 0.0352 1.2000 H

ATOM 731 HG3 GLN 47 3.514 -6.662 8.862 0.0352 1.2000 H

ATOM 732 CD GLN 47 2.327 -4.950 8.879 0.6951 1.7000 C

ATOM 733 OE1 GLN 47 1.750 -4.564 9.906 -0.6086 1.5000 O

ATOM 734 NE2 GLN 47 1.786 -4.831 7.662 -0.9407 1.5500 N

ATOM 735 HE21 GLN 47 2.294 -5.137 6.844 0.4251 1.3000 H

ATOM 736 HE22 GLN 47 0.872 -4.416 7.574 0.4251 1.3000 H

ATOM 737 C GLN 47 6.902 -5.514 11.045 0.5973 1.7000 C

ATOM 738 O GLN 47 7.792 -4.778 10.604 -0.5679 1.5000 O

ATOM 739 N CYS 48 6.908 -5.889 12.337 -0.4157 1.5500 N

ATOM 740 H CYS 48 6.202 -6.493 12.735 0.2719 1.3000 H

ATOM 741 CA CYS 48 8.020 -5.385 13.145 0.0213 1.7000 C

ATOM 742 HA CYS 48 8.114 -4.306 13.010 0.1124 1.2000 H

ATOM 743 CB CYS 48 7.836 -5.664 14.641 -0.1231 1.7000 C

ATOM 744 HB2 CYS 48 8.562 -5.059 15.185 0.1112 1.2000 H

ATOM 745 HB3 CYS 48 6.839 -5.342 14.943 0.1112 1.2000 H

ATOM 746 SG CYS 48 8.079 -7.396 15.138 -0.3119 1.8000 S

ATOM 747 HG CYS 48 7.001 -7.872 14.506 0.1933 1.2000 H

ATOM 748 C CYS 48 9.309 -6.071 12.682 0.5973 1.7000 C

ATOM 749 O CYS 48 10.398 -5.486 12.744 -0.5679 1.5000 O

ATOM 750 N LEU 49 9.173 -7.285 12.135 -0.4157 1.5500 N

ATOM 751 H LEU 49 8.262 -7.721 12.083 0.2719 1.3000 H

ATOM 752 CA LEU 49 10.320 -8.009 11.636 -0.0518 1.7000 C

ATOM 753 HA LEU 49 11.154 -7.878 12.323 0.0922 1.2000 H

ATOM 754 CB LEU 49 10.012 -9.503 11.505 -0.1102 1.7000 C

ATOM 755 HB2 LEU 49 9.148 -9.639 10.861 0.0457 1.2000 H

ATOM 756 HB3 LEU 49 10.856 -9.981 11.004 0.0457 1.2000 H

ATOM 757 CG LEU 49 9.755 -10.235 12.835 0.3531 1.7000 C

ATOM 758 HG LEU 49 8.970 -9.746 13.398 -0.0361 1.2000 H

ATOM 759 CD1 LEU 49 9.321 -11.651 12.546 -0.4121 1.7000 C

ATOM 760 HD11 LEU 49 9.123 -12.173 13.482 0.1000 1.2000 H

ATOM 761 HD12 LEU 49 8.406 -11.645 11.953 0.1000 1.2000 H

ATOM 762 HD13 LEU 49 10.101 -12.182 11.999 0.1000 1.2000 H

ATOM 763 CD2 LEU 49 10.998 -10.191 13.680 -0.4121 1.7000 C

ATOM 764 HD21 LEU 49 10.863 -10.824 14.558 0.1000 1.2000 H

ATOM 765 HD22 LEU 49 11.858 -10.554 13.116 0.1000 1.2000 H

ATOM 766 HD23 LEU 49 11.185 -9.178 14.028 0.1000 1.2000 H

ATOM 767 C LEU 49 10.741 -7.407 10.307 0.5973 1.7000 C

ATOM 768 O LEU 49 11.924 -7.385 9.993 -0.5679 1.5000 O

ATOM 769 N ALA 50 9.781 -6.881 9.535 -0.4157 1.5500 N

ATOM 770 H ALA 50 8.821 -6.908 9.846 0.2719 1.3000 H

ATOM 771 CA ALA 50 10.066 -6.239 8.255 0.0337 1.7000 C

ATOM 772 HA ALA 50 10.528 -6.964 7.585 0.0823 1.2000 H

ATOM 773 CB ALA 50 8.775 -5.729 7.629 -0.1825 1.7000 C

ATOM 774 HB1 ALA 50 8.982 -5.300 6.657 0.0603 1.2000 H

ATOM 775 HB2 ALA 50 8.056 -6.541 7.532 0.0603 1.2000 H

ATOM 776 HB3 ALA 50 8.336 -4.940 8.233 0.0603 1.2000 H

ATOM 777 C ALA 50 11.009 -5.068 8.457 0.5973 1.7000 C

ATOM 778 O ALA 50 11.971 -4.874 7.703 -0.5679 1.5000 O

ATOM 779 N GLU 51 10.767 -4.311 9.526 -0.5163 1.5500 N

ATOM 780 H GLU 51 9.970 -4.491 10.121 0.2936 1.3000 H

ATOM 781 CA GLU 51 11.635 -3.186 9.814 0.0397 1.7000 C

ATOM 782 HA GLU 51 11.775 -2.608 8.901 0.1105 1.2000 H

ATOM 783 CB GLU 51 11.020 -2.255 10.859 0.0560 1.7000 C

ATOM 784 HB2 GLU 51 10.050 -1.924 10.486 -0.0173 1.2000 H

ATOM 785 HB3 GLU 51 10.860 -2.807 11.786 -0.0173 1.2000 H

ATOM 786 CG GLU 51 11.877 -1.012 11.158 0.0136 1.7000 C

ATOM 787 HG2 GLU 51 12.832 -1.329 11.581 -0.0425 1.2000 H

ATOM 788 HG3 GLU 51 12.077 -0.493 10.219 -0.0425 1.2000 H

ATOM 789 CD GLU 51 11.230 -0.033 12.126 0.8054 1.7000 C

ATOM 790 OE1 GLU 51 10.136 -0.288 12.570 -0.8188 1.5000 O

ATOM 791 OE2 GLU 51 11.838 0.973 12.413 -0.8188 1.5000 O

ATOM 792 C GLU 51 12.997 -3.675 10.273 0.5366 1.7000 C

ATOM 793 O GLU 51 14.018 -3.255 9.721 -0.5819 1.5000 O

ATOM 794 N ARG 52 13.024 -4.609 11.232 -0.3479 1.5500 N

ATOM 795 H ARG 52 12.159 -4.943 11.635 0.2747 1.3000 H

ATOM 796 CA ARG 52 14.295 -5.090 11.773 -0.2637 1.7000 C

ATOM 797 HA ARG 52 14.847 -4.252 12.203 0.1560 1.2000 H

ATOM 798 CB ARG 52 14.009 -6.115 12.856 -0.0007 1.7000 C

ATOM 799 HB2 ARG 52 13.336 -6.875 12.455 0.0327 1.2000 H

ATOM 800 HB3 ARG 52 14.944 -6.608 13.127 0.0327 1.2000 H

ATOM 801 CG ARG 52 13.402 -5.520 14.129 0.0390 1.7000 C

ATOM 802 HG2 ARG 52 14.155 -4.901 14.619 0.0285 1.2000 H

ATOM 803 HG3 ARG 52 12.560 -4.879 13.875 0.0285 1.2000 H

ATOM 804 CD ARG 52 12.942 -6.570 15.077 0.0486 1.7000 C

ATOM 805 HD2 ARG 52 12.227 -7.206 14.555 0.0687 1.2000 H

ATOM 806 HD3 ARG 52 13.795 -7.175 15.386 0.0687 1.2000 H

ATOM 807 NE ARG 52 12.303 -6.003 16.253 -0.5295 1.5500 N

ATOM 808 HE ARG 52 12.430 -5.014 16.411 0.3456 1.3000 H

ATOM 809 CZ ARG 52 11.539 -6.700 17.119 0.8076 1.7000 C

ATOM 810 NH1 ARG 52 11.334 -7.985 16.934 -0.8627 1.5500 N

ATOM 811 HH11 ARG 52 10.786 -8.509 17.599 0.4478 1.3000 H

ATOM 812 HH12 ARG 52 11.734 -8.447 16.132 0.4478 1.3000 H

ATOM 813 NH2 ARG 52 10.993 -6.092 18.157 -0.8627 1.5500 N

ATOM 814 HH21 ARG 52 11.137 -5.103 18.295 0.4478 1.3000 H

ATOM 815 HH22 ARG 52 10.413 -6.612 18.797 0.4478 1.3000 H

ATOM 816 C ARG 52 15.146 -5.707 10.665 0.7341 1.7000 C

ATOM 817 O ARG 52 16.358 -5.480 10.590 -0.5894 1.5000 O

ATOM 818 N TYR 53 14.488 -6.440 9.769 -0.4157 1.5500 N

ATOM 819 H TYR 53 13.492 -6.541 9.868 0.2719 1.3000 H

ATOM 820 CA TYR 53 15.114 -7.050 8.617 -0.0014 1.7000 C

ATOM 821 HA TYR 53 15.874 -7.755 8.957 0.0876 1.2000 H

ATOM 822 CB TYR 53 14.073 -7.810 7.790 -0.0152 1.7000 C

ATOM 823 HB2 TYR 53 13.723 -8.660 8.378 0.0295 1.2000 H

ATOM 824 HB3 TYR 53 13.212 -7.166 7.618 0.0295 1.2000 H

ATOM 825 CG TYR 53 14.555 -8.331 6.444 -0.0011 1.7000 C

ATOM 826 CD1 TYR 53 15.324 -9.479 6.360 -0.1906 1.7000 C

ATOM 827 HD1 TYR 53 15.603 -10.015 7.256 0.1699 1.2000 H

ATOM 828 CE1 TYR 53 15.731 -9.947 5.121 -0.2341 1.7000 C

ATOM 829 HE1 TYR 53 16.331 -10.842 5.051 0.1656 1.2000 H

ATOM 830 CZ TYR 53 15.361 -9.265 3.965 0.3226 1.7000 C

ATOM 831 OH TYR 53 15.759 -9.745 2.736 -0.5579 1.5000 O

ATOM 832 HH TYR 53 15.385 -9.247 2.003 0.3992 1.2000 H

ATOM 833 CE2 TYR 53 14.597 -8.124 4.048 -0.2341 1.7000 C

ATOM 834 HE2 TYR 53 14.299 -7.608 3.148 0.1656 1.2000 H

ATOM 835 CD2 TYR 53 14.191 -7.652 5.279 -0.1906 1.7000 C

ATOM 836 HD2 TYR 53 13.589 -6.756 5.338 0.1699 1.2000 H

ATOM 837 C TYR 53 15.775 -6.008 7.760 0.5973 1.7000 C

ATOM 838 O TYR 53 16.954 -6.143 7.418 -0.5679 1.5000 O

ATOM 839 N SER 54 15.025 -4.959 7.397 -0.4157 1.5500 N

ATOM 840 H SER 54 14.063 -4.873 7.696 0.2719 1.3000 H

ATOM 841 CA SER 54 15.608 -3.952 6.546 -0.0249 1.7000 C

ATOM 842 HA SER 54 16.006 -4.438 5.656 0.0843 1.2000 H

ATOM 843 CB SER 54 14.562 -2.949 6.124 0.2117 1.7000 C

ATOM 844 HB2 SER 54 14.969 -2.319 5.334 0.0352 1.2000 H

ATOM 845 HB3 SER 54 13.686 -3.473 5.740 0.0352 1.2000 H

ATOM 846 OG SER 54 14.187 -2.132 7.196 -0.6546 1.5000 O

ATOM 847 HG SER 54 13.934 -2.683 7.944 0.4275 1.2000 H

ATOM 848 C SER 54 16.731 -3.203 7.235 0.5973 1.7000 C

ATOM 849 O SER 54 17.718 -2.856 6.605 -0.5679 1.5000 O

ATOM 850 N VAL 55 16.675 -3.049 8.553 -0.4157 1.5500 N

ATOM 851 H VAL 55 15.882 -3.362 9.095 0.2719 1.3000 H

ATOM 852 CA VAL 55 17.774 -2.351 9.196 -0.0875 1.7000 C

ATOM 853 HA VAL 55 17.933 -1.393 8.697 0.0969 1.2000 H

ATOM 854 CB VAL 55 17.479 -2.078 10.681 0.2985 1.7000 C

ATOM 855 HB VAL 55 17.179 -3.000 11.180 -0.0297 1.2000 H

ATOM 856 CG1 VAL 55 18.733 -1.535 11.361 -0.3192 1.7000 C

ATOM 857 HG11 VAL 55 18.484 -1.196 12.367 0.0791 1.2000 H

ATOM 858 HG12 VAL 55 19.496 -2.308 11.455 0.0791 1.2000 H

ATOM 859 HG13 VAL 55 19.138 -0.692 10.799 0.0791 1.2000 H

ATOM 860 CG2 VAL 55 16.345 -1.060 10.800 -0.3192 1.7000 C

ATOM 861 HG21 VAL 55 16.080 -0.924 11.849 0.0791 1.2000 H

ATOM 862 HG22 VAL 55 16.664 -0.101 10.390 0.0791 1.2000 H

ATOM 863 HG23 VAL 55 15.462 -1.382 10.260 0.0791 1.2000 H

ATOM 864 C VAL 55 19.042 -3.166 9.069 0.5973 1.7000 C

ATOM 865 O VAL 55 20.081 -2.667 8.636 -0.5679 1.5000 O

ATOM 866 N ILE 56 18.951 -4.452 9.371 -0.4157 1.5500 N

ATOM 867 H ILE 56 18.078 -4.845 9.694 0.2719 1.3000 H

ATOM 868 CA ILE 56 20.126 -5.288 9.296 -0.0597 1.7000 C

ATOM 869 HA ILE 56 20.915 -4.847 9.909 0.0869 1.2000 H

ATOM 870 CB ILE 56 19.806 -6.687 9.837 0.1303 1.7000 C

ATOM 871 HB ILE 56 18.920 -7.068 9.324 0.0187 1.2000 H

ATOM 872 CG2 ILE 56 20.968 -7.637 9.561 -0.3204 1.7000 C

ATOM 873 HG21 ILE 56 20.795 -8.595 10.050 0.0882 1.2000 H

ATOM 874 HG22 ILE 56 21.067 -7.839 8.494 0.0882 1.2000 H

ATOM 875 HG23 ILE 56 21.900 -7.213 9.940 0.0882 1.2000 H

ATOM 876 CG1 ILE 56 19.512 -6.600 11.341 -0.0430 1.7000 C

ATOM 877 HG12 ILE 56 20.427 -6.369 11.889 0.0236 1.2000 H

ATOM 878 HG13 ILE 56 18.808 -5.792 11.532 0.0236 1.2000 H

ATOM 879 CD1 ILE 56 18.893 -7.852 11.904 -0.0660 1.7000 C

ATOM 880 HD11 ILE 56 18.366 -7.611 12.828 0.0186 1.2000 H

ATOM 881 HD12 ILE 56 18.181 -8.280 11.197 0.0186 1.2000 H

ATOM 882 HD13 ILE 56 19.669 -8.585 12.127 0.0186 1.2000 H

ATOM 883 C ILE 56 20.636 -5.390 7.865 0.5973 1.7000 C

ATOM 884 O ILE 56 21.825 -5.172 7.597 -0.5679 1.5000 O

ATOM 885 N LEU 57 19.737 -5.694 6.928 -0.4157 1.5500 N

ATOM 886 H LEU 57 18.769 -5.833 7.176 0.2719 1.3000 H

ATOM 887 CA LEU 57 20.151 -5.863 5.550 -0.0518 1.7000 C

ATOM 888 HA LEU 57 20.968 -6.584 5.557 0.0922 1.2000 H

ATOM 889 CB LEU 57 19.022 -6.416 4.686 -0.1102 1.7000 C

ATOM 890 HB2 LEU 57 18.719 -7.386 5.084 0.0457 1.2000 H

ATOM 891 HB3 LEU 57 18.164 -5.745 4.758 0.0457 1.2000 H

ATOM 892 CG LEU 57 19.408 -6.563 3.212 0.3531 1.7000 C

ATOM 893 HG LEU 57 19.678 -5.599 2.784 -0.0361 1.2000 H

ATOM 894 CD1 LEU 57 20.592 -7.518 3.099 -0.4121 1.7000 C

ATOM 895 HD11 LEU 57 20.794 -7.719 2.047 0.1000 1.2000 H

ATOM 896 HD12 LEU 57 21.496 -7.090 3.527 0.1000 1.2000 H

ATOM 897 HD13 LEU 57 20.367 -8.461 3.598 0.1000 1.2000 H

ATOM 898 CD2 LEU 57 18.239 -7.073 2.428 -0.4121 1.7000 C

ATOM 899 HD21 LEU 57 18.511 -7.161 1.376 0.1000 1.2000 H

ATOM 900 HD22 LEU 57 17.932 -8.050 2.801 0.1000 1.2000 H

ATOM 901 HD23 LEU 57 17.410 -6.378 2.512 0.1000 1.2000 H

ATOM 902 C LEU 57 20.649 -4.593 4.902 0.5973 1.7000 C

ATOM 903 O LEU 57 21.701 -4.597 4.269 -0.5679 1.5000 O

ATOM 904 N LEU 58 19.908 -3.502 5.024 -0.4157 1.5500 N

ATOM 905 H LEU 58 19.062 -3.510 5.572 0.2719 1.3000 H

ATOM 906 CA LEU 58 20.304 -2.303 4.322 -0.0518 1.7000 C

ATOM 907 HA LEU 58 20.456 -2.533 3.268 0.0922 1.2000 H

ATOM 908 CB LEU 58 19.217 -1.248 4.453 -0.1102 1.7000 C

ATOM 909 HB2 LEU 58 19.052 -1.041 5.511 0.0457 1.2000 H

ATOM 910 HB3 LEU 58 19.590 -0.323 4.027 0.0457 1.2000 H

ATOM 911 CG LEU 58 17.859 -1.604 3.776 0.3531 1.7000 C

ATOM 912 HG LEU 58 17.439 -2.492 4.238 -0.0361 1.2000 H

ATOM 913 CD1 LEU 58 16.884 -0.453 4.028 -0.4121 1.7000 C

ATOM 914 HD11 LEU 58 15.925 -0.663 3.562 0.1000 1.2000 H

ATOM 915 HD12 LEU 58 16.733 -0.328 5.101 0.1000 1.2000 H

ATOM 916 HD13 LEU 58 17.277 0.476 3.615 0.1000 1.2000 H

ATOM 917 CD2 LEU 58 18.043 -1.939 2.325 -0.4121 1.7000 C

ATOM 918 HD21 LEU 58 17.074 -1.980 1.827 0.1000 1.2000 H

ATOM 919 HD22 LEU 58 18.656 -1.184 1.852 0.1000 1.2000 H

ATOM 920 HD23 LEU 58 18.522 -2.912 2.221 0.1000 1.2000 H

ATOM 921 C LEU 58 21.625 -1.774 4.869 0.5973 1.7000 C

ATOM 922 O LEU 58 22.497 -1.350 4.100 -0.5679 1.5000 O

ATOM 923 N ASP 59 21.827 -1.859 6.197 -0.5163 1.5500 N

ATOM 924 H ASP 59 21.105 -2.196 6.820 0.2936 1.3000 H

ATOM 925 CA ASP 59 23.096 -1.412 6.751 0.0381 1.7000 C

ATOM 926 HA ASP 59 23.306 -0.403 6.392 0.0880 1.2000 H

ATOM 927 CB ASP 59 23.074 -1.381 8.281 -0.0303 1.7000 C

ATOM 928 HB2 ASP 59 22.717 -2.339 8.662 -0.0122 1.2000 H

ATOM 929 HB3 ASP 59 24.096 -1.242 8.638 -0.0122 1.2000 H

ATOM 930 CG ASP 59 22.224 -0.236 8.861 0.7994 1.7000 C

ATOM 931 OD1 ASP 59 21.845 0.642 8.113 -0.8014 1.5000 O

ATOM 932 OD2 ASP 59 22.001 -0.227 10.054 -0.8014 1.5000 O

ATOM 933 C ASP 59 24.218 -2.319 6.265 0.5366 1.7000 C

ATOM 934 O ASP 59 25.313 -1.839 5.951 -0.5819 1.5000 O

ATOM 935 N THR 60 23.938 -3.627 6.163 -0.4157 1.5500 N

ATOM 936 H THR 60 23.024 -3.968 6.428 0.2719 1.3000 H

ATOM 937 CA THR 60 24.925 -4.575 5.684 -0.0389 1.7000 C

ATOM 938 HA THR 60 25.817 -4.495 6.308 0.1007 1.2000 H

ATOM 939 CB THR 60 24.411 -6.027 5.749 0.3654 1.7000 C

ATOM 940 HB THR 60 23.482 -6.115 5.189 0.0043 1.2000 H

ATOM 941 CG2 THR 60 25.435 -6.978 5.124 -0.2438 1.7000 C

ATOM 942 HG21 THR 60 25.151 -8.006 5.348 0.0642 1.2000 H

ATOM 943 HG22 THR 60 25.458 -6.866 4.040 0.0642 1.2000 H

ATOM 944 HG23 THR 60 26.427 -6.789 5.536 0.0642 1.2000 H

ATOM 945 OG1 THR 60 24.146 -6.390 7.114 -0.6761 1.5000 O

ATOM 946 HG1 THR 60 23.408 -5.855 7.431 0.4102 1.2000 H

ATOM 947 C THR 60 25.310 -4.275 4.255 0.5973 1.7000 C

ATOM 948 O THR 60 26.493 -4.248 3.925 -0.5679 1.5000 O

ATOM 949 N LEU 61 24.329 -4.015 3.401 -0.4157 1.5500 N

ATOM 950 H LEU 61 23.369 -3.990 3.713 0.2719 1.3000 H

ATOM 951 CA LEU 61 24.633 -3.771 2.007 -0.0518 1.7000 C

ATOM 952 HA LEU 61 25.190 -4.618 1.610 0.0922 1.2000 H

ATOM 953 CB LEU 61 23.321 -3.589 1.232 -0.1102 1.7000 C

ATOM 954 HB2 LEU 61 22.739 -2.826 1.748 0.0457 1.2000 H

ATOM 955 HB3 LEU 61 23.548 -3.205 0.236 0.0457 1.2000 H

ATOM 956 CG LEU 61 22.448 -4.846 1.089 0.3531 1.7000 C

ATOM 957 HG LEU 61 22.268 -5.291 2.059 -0.0361 1.2000 H

ATOM 958 CD1 LEU 61 21.109 -4.462 0.474 -0.4121 1.7000 C

ATOM 959 HD11 LEU 61 20.476 -5.345 0.387 0.1000 1.2000 H

ATOM 960 HD12 LEU 61 20.606 -3.734 1.110 0.1000 1.2000 H

ATOM 961 HD13 LEU 61 21.257 -4.032 -0.517 0.1000 1.2000 H

ATOM 962 CD2 LEU 61 23.154 -5.868 0.257 -0.4121 1.7000 C

ATOM 963 HD21 LEU 61 24.071 -6.194 0.742 0.1000 1.2000 H

ATOM 964 HD22 LEU 61 22.511 -6.740 0.137 0.1000 1.2000 H

ATOM 965 HD23 LEU 61 23.379 -5.464 -0.730 0.1000 1.2000 H

ATOM 966 C LEU 61 25.503 -2.529 1.837 0.5973 1.7000 C

ATOM 967 O LEU 61 26.486 -2.550 1.083 -0.5679 1.5000 O

ATOM 968 N LEU 62 25.196 -1.470 2.591 -0.4157 1.5500 N

ATOM 969 H LEU 62 24.403 -1.494 3.219 0.2719 1.3000 H

ATOM 970 CA LEU 62 25.979 -0.247 2.484 -0.0518 1.7000 C

ATOM 971 HA LEU 62 26.145 -0.030 1.430 0.0922 1.2000 H

ATOM 972 CB LEU 62 25.216 0.926 3.114 -0.1102 1.7000 C

ATOM 973 HB2 LEU 62 24.939 0.643 4.131 0.0457 1.2000 H

ATOM 974 HB3 LEU 62 25.896 1.776 3.192 0.0457 1.2000 H

ATOM 975 CG LEU 62 23.945 1.390 2.368 0.3531 1.7000 C

ATOM 976 HG LEU 62 23.269 0.547 2.228 -0.0361 1.2000 H

ATOM 977 CD1 LEU 62 23.233 2.453 3.191 -0.4121 1.7000 C

ATOM 978 HD11 LEU 62 22.413 2.882 2.620 0.1000 1.2000 H

ATOM 979 HD12 LEU 62 22.839 2.007 4.106 0.1000 1.2000 H

ATOM 980 HD13 LEU 62 23.926 3.253 3.452 0.1000 1.2000 H

ATOM 981 CD2 LEU 62 24.339 1.940 1.005 -0.4121 1.7000 C

ATOM 982 HD21 LEU 62 23.470 2.371 0.511 0.1000 1.2000 H

ATOM 983 HD22 LEU 62 25.098 2.716 1.114 0.1000 1.2000 H

ATOM 984 HD23 LEU 62 24.723 1.145 0.374 0.1000 1.2000 H

ATOM 985 C LEU 62 27.364 -0.363 3.125 0.5973 1.7000 C

ATOM 986 O LEU 62 28.340 0.175 2.597 -0.5679 1.5000 O

ATOM 987 N GLY 63 27.464 -1.064 4.257 -0.4157 1.5500 N

ATOM 988 H GLY 63 26.642 -1.481 4.670 0.2719 1.3000 H

ATOM 989 CA GLY 63 28.740 -1.194 4.952 -0.0252 1.7000 C

ATOM 990 HA2 GLY 63 29.252 -0.231 4.970 0.0698 1.2000 H

ATOM 991 HA3 GLY 63 28.532 -1.488 5.981 0.0698 1.2000 H

ATOM 992 C GLY 63 29.662 -2.245 4.333 0.5973 1.7000 C

ATOM 993 O GLY 63 30.886 -2.119 4.393 -0.5679 1.5000 O

ATOM 994 N ARG 64 29.079 -3.271 3.720 -0.3479 1.5500 N

ATOM 995 H ARG 64 28.072 -3.311 3.677 0.2747 1.3000 H

ATOM 996 CA ARG 64 29.836 -4.372 3.146 -0.2637 1.7000 C

ATOM 997 HA ARG 64 30.674 -4.601 3.807 0.1560 1.2000 H

ATOM 998 CB ARG 64 28.962 -5.620 3.045 -0.0007 1.7000 C

ATOM 999 HB2 ARG 64 28.597 -5.857 4.046 0.0327 1.2000 H

ATOM 1000 HB3 ARG 64 28.107 -5.390 2.409 0.0327 1.2000 H

ATOM 1001 CG ARG 64 29.635 -6.850 2.486 0.0390 1.7000 C

ATOM 1002 HG2 ARG 64 29.901 -6.682 1.442 0.0285 1.2000 H

ATOM 1003 HG3 ARG 64 30.547 -7.043 3.052 0.0285 1.2000 H

ATOM 1004 CD ARG 64 28.762 -8.063 2.565 0.0486 1.7000 C

ATOM 1005 HD2 ARG 64 29.334 -8.924 2.216 0.0687 1.2000 H

ATOM 1006 HD3 ARG 64 28.492 -8.229 3.609 0.0687 1.2000 H

ATOM 1007 NE ARG 64 27.558 -7.946 1.761 -0.5295 1.5500 N

ATOM 1008 HE ARG 64 27.501 -7.168 1.121 0.3456 1.3000 H

ATOM 1009 CZ ARG 64 26.518 -8.802 1.825 0.8076 1.7000 C

ATOM 1010 NH1 ARG 64 26.558 -9.823 2.652 -0.8627 1.5500 N

ATOM 1011 HH11 ARG 64 27.370 -9.967 3.233 0.4478 1.3000 H

ATOM 1012 HH12 ARG 64 25.775 -10.455 2.715 0.4478 1.3000 H

ATOM 1013 NH2 ARG 64 25.460 -8.625 1.060 -0.8627 1.5500 N

ATOM 1014 HH21 ARG 64 25.422 -7.843 0.424 0.4478 1.3000 H

ATOM 1015 HH22 ARG 64 24.689 -9.273 1.110 0.4478 1.3000 H

ATOM 1016 C ARG 64 30.398 -4.052 1.775 0.7341 1.7000 C

ATOM 1017 O ARG 64 31.569 -4.312 1.503 -0.5894 1.5000 O

ATOM 1018 N MET 65 29.578 -3.502 0.889 -0.4157 1.5500 N

ATOM 1019 H MET 65 28.627 -3.263 1.133 0.2719 1.3000 H

ATOM 1020 CA MET 65 30.054 -3.280 -0.463 -0.0237 1.7000 C

ATOM 1021 HA MET 65 30.724 -4.097 -0.714 0.0880 1.2000 H

ATOM 1022 CB MET 65 28.852 -3.384 -1.391 0.0342 1.7000 C

ATOM 1023 HB2 MET 65 28.083 -2.712 -1.030 0.0241 1.2000 H

ATOM 1024 HB3 MET 65 29.125 -3.092 -2.403 0.0241 1.2000 H

ATOM 1025 CG MET 65 28.290 -4.725 -1.429 0.0018 1.7000 C

ATOM 1026 HG2 MET 65 29.068 -5.395 -1.792 0.0440 1.2000 H

ATOM 1027 HG3 MET 65 27.999 -5.040 -0.427 0.0440 1.2000 H

ATOM 1028 SD MET 65 26.881 -4.898 -2.496 -0.2737 1.8000 S

ATOM 1029 CE MET 65 25.665 -3.952 -1.663 -0.0536 1.7000 C

ATOM 1030 HE1 MET 65 24.714 -4.042 -2.188 0.0684 1.2000 H

ATOM 1031 HE2 MET 65 25.556 -4.326 -0.647 0.0684 1.2000 H

ATOM 1032 HE3 MET 65 25.955 -2.902 -1.642 0.0684 1.2000 H

ATOM 1033 C MET 65 30.796 -1.938 -0.631 0.5973 1.7000 C

ATOM 1034 O MET 65 30.124 -1.037 -1.139 -0.5679 1.5000 O

ATOM 1035 N LEU 66 32.097 -2.018 -1.000 -0.4157 1.5500 N

ATOM 1036 H LEU 66 32.615 -1.164 -0.852 0.2719 1.3000 H

ATOM 1037 CA LEU 66 32.732 -2.893 -2.021 -0.0518 1.7000 C

ATOM 1038 HA LEU 66 33.366 -2.258 -2.635 0.0922 1.2000 H

ATOM 1039 CB LEU 66 33.618 -3.923 -1.319 -0.1102 1.7000 C

ATOM 1040 HB2 LEU 66 33.003 -4.499 -0.628 0.0457 1.2000 H

ATOM 1041 HB3 LEU 66 34.014 -4.613 -2.067 0.0457 1.2000 H

ATOM 1042 CG LEU 66 34.795 -3.382 -0.565 0.3531 1.7000 C

ATOM 1043 HG LEU 66 34.470 -2.625 0.150 -0.0361 1.2000 H

ATOM 1044 CD1 LEU 66 35.453 -4.517 0.200 -0.4121 1.7000 C

ATOM 1045 HD11 LEU 66 36.310 -4.135 0.756 0.1000 1.2000 H

ATOM 1046 HD12 LEU 66 34.746 -4.940 0.914 0.1000 1.2000 H

ATOM 1047 HD13 LEU 66 35.791 -5.297 -0.483 0.1000 1.2000 H

ATOM 1048 CD2 LEU 66 35.754 -2.765 -1.544 -0.4121 1.7000 C

ATOM 1049 HD21 LEU 66 36.650 -2.446 -1.010 0.1000 1.2000 H

ATOM 1050 HD22 LEU 66 36.047 -3.495 -2.298 0.1000 1.2000 H

ATOM 1051 HD23 LEU 66 35.329 -1.881 -2.016 0.1000 1.2000 H

ATOM 1052 C LEU 66 31.844 -3.670 -2.991 0.5973 1.7000 C

ATOM 1053 O LEU 66 31.674 -4.876 -2.795 -0.5679 1.5000 O

ATOM 1054 N PRO 67 31.271 -3.063 -4.033 -0.2548 1.5500 N

ATOM 1055 CD PRO 67 31.402 -1.614 -4.251 0.0192 1.7000 C

ATOM 1056 HD2 PRO 67 32.395 -1.376 -4.630 0.0391 1.2000 H

ATOM 1057 HD3 PRO 67 31.170 -1.026 -3.367 0.0391 1.2000 H

ATOM 1058 CG PRO 67 30.355 -1.381 -5.329 0.0189 1.7000 C

ATOM 1059 HG2 PRO 67 30.617 -0.536 -5.966 0.0213 1.2000 H

ATOM 1060 HG3 PRO 67 29.384 -1.214 -4.861 0.0213 1.2000 H

ATOM 1061 CB PRO 67 30.331 -2.679 -6.105 -0.0070 1.7000 C

ATOM 1062 HB2 PRO 67 31.185 -2.697 -6.783 0.0253 1.2000 H

ATOM 1063 HB3 PRO 67 29.406 -2.795 -6.666 0.0253 1.2000 H

ATOM 1064 CA PRO 67 30.479 -3.757 -5.027 -0.0266 1.7000 C

ATOM 1065 HA PRO 67 29.512 -4.021 -4.603 0.0641 1.2000 H

ATOM 1066 C PRO 67 31.198 -4.998 -5.552 0.5896 1.7000 C

ATOM 1067 O PRO 67 30.576 -6.040 -5.775 -0.5748 1.5000 O

ATOM 1068 N GLN 68 32.526 -4.903 -5.705 -0.4157 1.5500 N

ATOM 1069 H GLN 68 32.978 -4.057 -5.418 0.2719 1.3000 H

ATOM 1070 CA GLN 68 33.324 -6.030 -6.172 -0.0031 1.7000 C

ATOM 1071 HA GLN 68 32.864 -6.406 -7.087 0.0850 1.2000 H

ATOM 1072 CB GLN 68 34.741 -5.570 -6.534 -0.0036 1.7000 C

ATOM 1073 HB2 GLN 68 35.288 -6.449 -6.878 0.0171 1.2000 H

ATOM 1074 HB3 GLN 68 34.700 -4.883 -7.378 0.0171 1.2000 H

ATOM 1075 CG GLN 68 35.547 -4.938 -5.395 -0.0645 1.7000 C

ATOM 1076 HG2 GLN 68 35.329 -5.405 -4.436 0.0352 1.2000 H

ATOM 1077 HG3 GLN 68 36.605 -5.106 -5.606 0.0352 1.2000 H

ATOM 1078 CD GLN 68 35.309 -3.437 -5.307 0.6951 1.7000 C

ATOM 1079 OE1 GLN 68 34.225 -2.934 -5.616 -0.6086 1.5000 O

ATOM 1080 NE2 GLN 68 36.337 -2.707 -4.891 -0.9407 1.5500 N

ATOM 1081 HE21 GLN 68 37.205 -3.152 -4.632 0.4251 1.3000 H

ATOM 1082 HE22 GLN 68 36.231 -1.708 -4.792 0.4251 1.3000 H

ATOM 1083 C GLN 68 33.378 -7.192 -5.186 0.5973 1.7000 C

ATOM 1084 O GLN 68 33.589 -8.336 -5.584 -0.5679 1.5000 O

ATOM 1085 N LEU 69 33.172 -6.923 -3.898 -0.4157 1.5500 N

ATOM 1086 H LEU 69 32.986 -5.981 -3.587 0.2719 1.3000 H

ATOM 1087 CA LEU 69 33.204 -7.995 -2.927 -0.0518 1.7000 C

ATOM 1088 HA LEU 69 34.112 -8.582 -3.075 0.0922 1.2000 H

ATOM 1089 CB LEU 69 33.172 -7.460 -1.497 -0.1102 1.7000 C

ATOM 1090 HB2 LEU 69 34.070 -6.862 -1.342 0.0457 1.2000 H

ATOM 1091 HB3 LEU 69 32.311 -6.804 -1.383 0.0457 1.2000 H

ATOM 1092 CG LEU 69 33.094 -8.504 -0.413 0.3531 1.7000 C

ATOM 1093 HG LEU 69 32.192 -9.100 -0.540 -0.0361 1.2000 H

ATOM 1094 CD1 LEU 69 34.301 -9.411 -0.481 -0.4121 1.7000 C

ATOM 1095 HD11 LEU 69 34.290 -10.093 0.370 0.1000 1.2000 H

ATOM 1096 HD12 LEU 69 34.278 -10.016 -1.387 0.1000 1.2000 H

ATOM 1097 HD13 LEU 69 35.222 -8.827 -0.451 0.1000 1.2000 H

ATOM 1098 CD2 LEU 69 32.993 -7.804 0.924 -0.4121 1.7000 C

ATOM 1099 HD21 LEU 69 32.840 -8.540 1.714 0.1000 1.2000 H

ATOM 1100 HD22 LEU 69 33.910 -7.251 1.131 0.1000 1.2000 H

ATOM 1101 HD23 LEU 69 32.154 -7.110 0.926 0.1000 1.2000 H

ATOM 1102 C LEU 69 32.012 -8.857 -3.147 0.5973 1.7000 C

ATOM 1103 O LEU 69 32.126 -10.065 -3.352 -0.5679 1.5000 O

ATOM 1104 N VAL 70 30.855 -8.222 -3.159 -0.4157 1.5500 N

ATOM 1105 H VAL 70 30.800 -7.224 -3.008 0.2719 1.3000 H

ATOM 1106 CA VAL 70 29.652 -8.999 -3.334 -0.0875 1.7000 C

ATOM 1107 HA VAL 70 29.694 -9.818 -2.615 0.0969 1.2000 H

ATOM 1108 CB VAL 70 28.405 -8.211 -3.019 0.2985 1.7000 C

ATOM 1109 HB VAL 70 28.432 -7.238 -3.510 -0.0297 1.2000 H

ATOM 1110 CG1 VAL 70 27.174 -8.977 -3.454 -0.3192 1.7000 C

ATOM 1111 HG11 VAL 70 26.281 -8.469 -3.087 0.0791 1.2000 H

ATOM 1112 HG12 VAL 70 27.098 -9.008 -4.541 0.0791 1.2000 H

ATOM 1113 HG13 VAL 70 27.188 -9.993 -3.055 0.0791 1.2000 H

ATOM 1114 CG2 VAL 70 28.361 -8.054 -1.502 -0.3192 1.7000 C

ATOM 1115 HG21 VAL 70 27.473 -7.491 -1.217 0.0791 1.2000 H

ATOM 1116 HG22 VAL 70 28.316 -9.031 -1.018 0.0791 1.2000 H

ATOM 1117 HG23 VAL 70 29.243 -7.526 -1.139 0.0791 1.2000 H

ATOM 1118 C VAL 70 29.591 -9.656 -4.678 0.5973 1.7000 C

ATOM 1119 O VAL 70 29.242 -10.830 -4.744 -0.5679 1.5000 O

ATOM 1120 N CYX 71 29.963 -8.958 -5.750 -0.4157 1.5500 N

ATOM 1121 H CYX 71 30.252 -7.992 -5.689 0.2719 1.3000 H

ATOM 1122 CA CYX 71 29.918 -9.635 -7.027 0.0429 1.7000 C

ATOM 1123 HA CYX 71 28.896 -9.961 -7.222 0.0766 1.2000 H

ATOM 1124 CB CYX 71 30.349 -8.753 -8.164 -0.0790 1.7000 C

ATOM 1125 HB2 CYX 71 31.326 -8.322 -7.944 0.0910 1.2000 H

ATOM 1126 HB3 CYX 71 30.451 -9.398 -9.035 0.0910 1.2000 H

ATOM 1127 SG CYX 71 29.253 -7.468 -8.619 -0.1081 1.8000 S

ATOM 1128 C CYX 71 30.825 -10.861 -7.022 0.5973 1.7000 C

ATOM 1129 O CYX 71 30.462 -11.904 -7.565 -0.5679 1.5000 O

ATOM 1130 N ARG 72 32.007 -10.781 -6.408 -0.3479 1.5500 N

ATOM 1131 H ARG 72 32.306 -9.936 -5.940 0.2747 1.3000 H

ATOM 1132 CA ARG 72 32.849 -11.967 -6.380 -0.2637 1.7000 C

ATOM 1133 HA ARG 72 32.946 -12.358 -7.391 0.1560 1.2000 H

ATOM 1134 CB ARG 72 34.240 -11.612 -5.880 -0.0007 1.7000 C

ATOM 1135 HB2 ARG 72 34.156 -11.063 -4.941 0.0327 1.2000 H

ATOM 1136 HB3 ARG 72 34.786 -12.536 -5.679 0.0327 1.2000 H

ATOM 1137 CG ARG 72 35.055 -10.790 -6.885 0.0390 1.7000 C

ATOM 1138 HG2 ARG 72 35.260 -11.419 -7.747 0.0285 1.2000 H

ATOM 1139 HG3 ARG 72 34.482 -9.932 -7.231 0.0285 1.2000 H

ATOM 1140 CD ARG 72 36.349 -10.339 -6.361 0.0486 1.7000 C

ATOM 1141 HD2 ARG 72 36.176 -9.720 -5.479 0.0687 1.2000 H

ATOM 1142 HD3 ARG 72 36.942 -11.209 -6.075 0.0687 1.2000 H

ATOM 1143 NE ARG 72 37.078 -9.563 -7.360 -0.5295 1.5500 N

ATOM 1144 HE ARG 72 36.676 -9.514 -8.285 0.3456 1.3000 H

ATOM 1145 CZ ARG 72 38.248 -8.939 -7.143 0.8076 1.7000 C

ATOM 1146 NH1 ARG 72 38.819 -9.006 -5.959 -0.8627 1.5500 N

ATOM 1147 HH11 ARG 72 39.705 -8.552 -5.799 0.4478 1.3000 H

ATOM 1148 HH12 ARG 72 38.389 -9.551 -5.228 0.4478 1.3000 H

ATOM 1149 NH2 ARG 72 38.827 -8.257 -8.115 -0.8627 1.5500 N

ATOM 1150 HH21 ARG 72 39.727 -7.826 -7.965 0.4478 1.3000 H

ATOM 1151 HH22 ARG 72 38.411 -8.236 -9.035 0.4478 1.3000 H

ATOM 1152 C ARG 72 32.215 -13.082 -5.535 0.7341 1.7000 C

ATOM 1153 O ARG 72 32.220 -14.248 -5.934 -0.5894 1.5000 O

ATOM 1154 N LEU 73 31.576 -12.718 -4.419 -0.4157 1.5500 N

ATOM 1155 H LEU 73 31.555 -11.745 -4.151 0.2719 1.3000 H

ATOM 1156 CA LEU 73 30.948 -13.694 -3.524 -0.0518 1.7000 C

ATOM 1157 HA LEU 73 31.697 -14.432 -3.238 0.0922 1.2000 H

ATOM 1158 CB LEU 73 30.413 -12.995 -2.260 -0.1102 1.7000 C

ATOM 1159 HB2 LEU 73 29.761 -12.179 -2.568 0.0457 1.2000 H

ATOM 1160 HB3 LEU 73 29.790 -13.706 -1.713 0.0457 1.2000 H

ATOM 1161 CG LEU 73 31.485 -12.435 -1.274 0.3531 1.7000 C

ATOM 1162 HG LEU 73 32.196 -11.805 -1.790 -0.0361 1.2000 H

ATOM 1163 CD1 LEU 73 30.796 -11.610 -0.174 -0.4121 1.7000 C

ATOM 1164 HD11 LEU 73 31.543 -11.239 0.529 0.1000 1.2000 H

ATOM 1165 HD12 LEU 73 30.278 -10.761 -0.617 0.1000 1.2000 H

ATOM 1166 HD13 LEU 73 30.077 -12.229 0.364 0.1000 1.2000 H

ATOM 1167 CD2 LEU 73 32.264 -13.581 -0.685 -0.4121 1.7000 C

ATOM 1168 HD21 LEU 73 32.955 -13.202 0.069 0.1000 1.2000 H

ATOM 1169 HD22 LEU 73 31.591 -14.299 -0.215 0.1000 1.2000 H

ATOM 1170 HD23 LEU 73 32.852 -14.082 -1.453 0.1000 1.2000 H

ATOM 1171 C LEU 73 29.809 -14.460 -4.202 0.5973 1.7000 C

ATOM 1172 O LEU 73 29.588 -15.638 -3.911 -0.5679 1.5000 O

ATOM 1173 N VAL 74 29.106 -13.801 -5.123 -0.4157 1.5500 N

ATOM 1174 H VAL 74 29.333 -12.839 -5.329 0.2719 1.3000 H

ATOM 1175 CA VAL 74 27.981 -14.420 -5.824 -0.0875 1.7000 C

ATOM 1176 HA VAL 74 27.633 -15.296 -5.275 0.0969 1.2000 H

ATOM 1177 CB VAL 74 26.805 -13.445 -5.957 0.2985 1.7000 C

ATOM 1178 HB VAL 74 25.970 -13.962 -6.433 -0.0297 1.2000 H

ATOM 1179 CG1 VAL 74 26.334 -12.984 -4.588 -0.3192 1.7000 C

ATOM 1180 HG11 VAL 74 25.460 -12.342 -4.700 0.0791 1.2000 H

ATOM 1181 HG12 VAL 74 26.057 -13.850 -3.985 0.0791 1.2000 H

ATOM 1182 HG13 VAL 74 27.113 -12.427 -4.067 0.0791 1.2000 H

ATOM 1183 CG2 VAL 74 27.218 -12.322 -6.803 -0.3192 1.7000 C

ATOM 1184 HG21 VAL 74 26.381 -12.090 -7.456 0.0791 1.2000 H

ATOM 1185 HG22 VAL 74 27.396 -11.431 -6.208 0.0791 1.2000 H

ATOM 1186 HG23 VAL 74 28.077 -12.494 -7.439 0.0791 1.2000 H

ATOM 1187 C VAL 74 28.362 -14.874 -7.239 0.5973 1.7000 C

ATOM 1188 O VAL 74 27.484 -15.183 -8.047 -0.5679 1.5000 O

ATOM 1189 N LEU 75 29.665 -14.886 -7.545 -0.4157 1.5500 N

ATOM 1190 H LEU 75 30.329 -14.621 -6.831 0.2719 1.3000 H

ATOM 1191 CA LEU 75 30.214 -15.312 -8.834 -0.0518 1.7000 C

ATOM 1192 HA LEU 75 31.297 -15.218 -8.768 0.0922 1.2000 H

ATOM 1193 CB LEU 75 29.854 -16.783 -9.079 -0.1102 1.7000 C

ATOM 1194 HB2 LEU 75 28.785 -16.888 -9.260 0.0457 1.2000 H

ATOM 1195 HB3 LEU 75 30.361 -17.113 -9.988 0.0457 1.2000 H

ATOM 1196 CG LEU 75 30.270 -17.744 -7.950 0.3531 1.7000 C

ATOM 1197 HG LEU 75 29.751 -17.481 -7.028 -0.0361 1.2000 H

ATOM 1198 CD1 LEU 75 29.859 -19.155 -8.317 -0.4121 1.7000 C

ATOM 1199 HD11 LEU 75 30.112 -19.834 -7.502 0.1000 1.2000 H

ATOM 1200 HD12 LEU 75 28.781 -19.195 -8.480 0.1000 1.2000 H

ATOM 1201 HD13 LEU 75 30.373 -19.477 -9.223 0.1000 1.2000 H

ATOM 1202 CD2 LEU 75 31.762 -17.627 -7.717 -0.4121 1.7000 C

ATOM 1203 HD21 LEU 75 32.080 -18.400 -7.017 0.1000 1.2000 H

ATOM 1204 HD22 LEU 75 32.307 -17.754 -8.653 0.1000 1.2000 H

ATOM 1205 HD23 LEU 75 32.010 -16.663 -7.274 0.1000 1.2000 H

ATOM 1206 C LEU 75 29.793 -14.479 -10.059 0.5973 1.7000 C

ATOM 1207 O LEU 75 29.614 -15.021 -11.152 -0.5679 1.5000 O

ATOM 1208 N ARG 76 29.673 -13.158 -9.889 -0.3479 1.5500 N

ATOM 1209 H ARG 76 29.797 -12.775 -8.966 0.2747 1.3000 H

ATOM 1210 CA ARG 76 29.368 -12.238 -10.987 -0.2637 1.7000 C

ATOM 1211 HA ARG 76 29.151 -12.802 -11.894 0.1560 1.2000 H

ATOM 1212 CB ARG 76 28.166 -11.355 -10.679 -0.0007 1.7000 C

ATOM 1213 HB2 ARG 76 28.356 -10.825 -9.744 0.0327 1.2000 H

ATOM 1214 HB3 ARG 76 28.071 -10.609 -11.468 0.0327 1.2000 H

ATOM 1215 CG ARG 76 26.815 -12.064 -10.592 0.0390 1.7000 C

ATOM 1216 HG2 ARG 76 26.670 -12.676 -11.482 0.0285 1.2000 H

ATOM 1217 HG3 ARG 76 26.802 -12.726 -9.726 0.0285 1.2000 H

ATOM 1218 CD ARG 76 25.681 -11.087 -10.501 0.0486 1.7000 C

ATOM 1219 HD2 ARG 76 25.704 -10.440 -11.380 0.0687 1.2000 H

ATOM 1220 HD3 ARG 76 24.745 -11.647 -10.511 0.0687 1.2000 H

ATOM 1221 NE ARG 76 25.727 -10.268 -9.288 -0.5295 1.5500 N

ATOM 1222 HE ARG 76 26.449 -10.488 -8.618 0.3456 1.3000 H

ATOM 1223 CZ ARG 76 24.891 -9.238 -9.029 0.8076 1.7000 C

ATOM 1224 NH1 ARG 76 23.943 -8.938 -9.890 -0.8627 1.5500 N

ATOM 1225 HH11 ARG 76 23.853 -9.480 -10.737 0.4478 1.3000 H

ATOM 1226 HH12 ARG 76 23.292 -8.194 -9.692 0.4478 1.3000 H

ATOM 1227 NH2 ARG 76 25.016 -8.526 -7.920 -0.8627 1.5500 N

ATOM 1228 HH21 ARG 76 25.743 -8.748 -7.257 0.4478 1.3000 H

ATOM 1229 HH22 ARG 76 24.378 -7.766 -7.738 0.4478 1.3000 H

ATOM 1230 C ARG 76 30.559 -11.334 -11.305 0.7341 1.7000 C

ATOM 1231 O ARG 76 30.443 -10.383 -12.073 -0.5894 1.5000 O

ATOM 1232 N CYX 77 31.706 -11.650 -10.718 -0.4157 1.5500 N

ATOM 1233 H CYX 77 31.737 -12.453 -10.106 0.2719 1.3000 H

ATOM 1234 CA CYX 77 32.949 -10.901 -10.899 0.0429 1.7000 C

ATOM 1235 HA CYX 77 33.056 -10.664 -11.958 0.0766 1.2000 H

ATOM 1236 CB CYX 77 32.962 -9.599 -10.119 -0.0790 1.7000 C

ATOM 1237 HB2 CYX 77 32.086 -9.015 -10.397 0.0910 1.2000 H

ATOM 1238 HB3 CYX 77 32.905 -9.829 -9.055 0.0910 1.2000 H

ATOM 1239 SG CYX 77 34.405 -8.591 -10.406 -0.1081 1.8000 S

ATOM 1240 C CYX 77 34.142 -11.728 -10.472 0.5973 1.7000 C

ATOM 1241 O CYX 77 34.039 -12.547 -9.561 -0.5679 1.5000 O

ATOM 1242 N SER 78 35.269 -11.542 -11.141 -0.4157 1.5500 N

ATOM 1243 H SER 78 35.317 -10.882 -11.905 0.2719 1.3000 H

ATOM 1244 CA SER 78 36.491 -12.210 -10.732 -0.0249 1.7000 C

ATOM 1245 HA SER 78 36.487 -12.376 -9.654 0.0843 1.2000 H

ATOM 1246 CB SER 78 36.625 -13.553 -11.431 0.2117 1.7000 C

ATOM 1247 HB2 SER 78 37.474 -14.094 -11.013 0.0352 1.2000 H

ATOM 1248 HB3 SER 78 35.722 -14.143 -11.271 0.0352 1.2000 H

ATOM 1249 OG SER 78 36.833 -13.377 -12.811 -0.6546 1.5000 O

ATOM 1250 HG SER 78 37.668 -12.912 -12.944 0.4275 1.2000 H

ATOM 1251 C SER 78 37.693 -11.354 -11.084 0.5973 1.7000 C

ATOM 1252 O SER 78 37.609 -10.494 -11.959 -0.5679 1.5000 O

ATOM 1253 N MET 79 38.777 -11.598 -10.361 -0.3821 1.5500 N

ATOM 1254 H MET 79 38.720 -12.367 -9.717 0.2681 1.3000 H

ATOM 1255 CA MET 79 40.101 -11.032 -10.571 -0.2597 1.7000 C

ATOM 1256 HA MET 79 40.153 -10.087 -10.032 0.1277 1.2000 H

ATOM 1257 CB MET 79 41.114 -11.979 -9.959 -0.0236 1.7000 C

ATOM 1258 HB2 MET 79 40.995 -12.983 -10.369 0.0480 1.2000 H

ATOM 1259 HB3 MET 79 42.115 -11.624 -10.209 0.0480 1.2000 H

ATOM 1260 CG MET 79 40.992 -12.016 -8.441 0.0492 1.7000 C

ATOM 1261 HG2 MET 79 41.834 -12.579 -8.038 0.0317 1.2000 H

ATOM 1262 HG3 MET 79 41.031 -11.001 -8.045 0.0317 1.2000 H

ATOM 1263 SD MET 79 39.450 -12.820 -7.903 -0.2692 1.8000 S

ATOM 1264 CE MET 79 39.633 -12.787 -6.127 -0.0376 1.7000 C

ATOM 1265 HE1 MET 79 38.750 -13.226 -5.663 0.0625 1.2000 H

ATOM 1266 HE2 MET 79 40.514 -13.361 -5.841 0.0625 1.2000 H

ATOM 1267 HE3 MET 79 39.746 -11.757 -5.790 0.0625 1.2000 H

ATOM 1268 C MET 79 40.395 -10.713 -12.032 0.8013 1.7000 C

ATOM 1269 O MET 79 41.008 -9.686 -12.324 -0.8105 1.5000 O

ATOM 1270 OXT MET 79 40.395 -11.660 -12.820 -0.8105 1.5000 O

TER 1271 MET 79

END
